# Unveiling vertebrate development dynamics in frog *Xenopus laevis* using micro-CT imaging

**DOI:** 10.1093/gigascience/giae037

**Published:** 2024-07-17

**Authors:** Jakub Laznovsky, Michaela Kavkova, Alice Helena Reis, Pavla Robovska-Havelkova, Lorena Agostini Maia, Jan Krivanek, Tomas Zikmund, Jozef Kaiser, Marcela Buchtova, Jakub Harnos

**Affiliations:** Central European Institute of Technology, Brno University of Technology, 612 00 Brno, Czech Republic; Central European Institute of Technology, Brno University of Technology, 612 00 Brno, Czech Republic; Department of Chemical Engineering, Columbia University, New York, NY 10025, USA, and Department of Genetics and Development, Columbia Stem Cell Initiative, Columbia University Irving Medical Center, New York, NY 10032, USA; Department of Zoology, Faculty of Science, University of South Bohemia, 370 05 Ceske Budejovice, Czech Republic; Department of Experimental Biology, Faculty of Science, Masaryk University, 625 00 Brno, Czech Republic; Department of Histology and Embryology, Faculty of Medicine, Masaryk University, 625 00 Brno, Czech Republic; Central European Institute of Technology, Brno University of Technology, 612 00 Brno, Czech Republic; Central European Institute of Technology, Brno University of Technology, 612 00 Brno, Czech Republic; Institute of Physical Engineering, Faculty of Mechanical Engineering, Brno University of Technology, 616 69 Brno, Czech Republic; Department of Experimental Biology, Faculty of Science, Masaryk University, 625 00 Brno, Czech Republic; Laboratory of Molecular Morphogenesis, Institute of Animal Physiology and Genetics, v.v.i., Czech Academy of Sciences, 602 00 Brno, Czech Republic; Department of Experimental Biology, Faculty of Science, Masaryk University, 625 00 Brno, Czech Republic

**Keywords:** *Xenopus laevis*, development, vertebrates, micro–computed tomography, morphological changes

## Abstract

**Background:**

*Xenopus laevis*, the African clawed frog, is a versatile vertebrate model organism in various biological disciplines, prominently in developmental biology to study body plan reorganization during metamorphosis. However, a notable gap exists in the availability of comprehensive datasets encompassing *Xenopus*’ late developmental stages.

**Findings:**

This study utilized micro–computed tomography (micro-CT), a noninvasive 3-dimensional (3D) imaging technique with micrometer-scale resolution, to explore the developmental dynamics and morphological changes in *Xenopus laevis*. Our approach involved generating high-resolution images and computed 3D models of developing *Xenopus* specimens, spanning from premetamorphosis tadpoles to fully mature adults. This dataset enhances our understanding of vertebrate development and supports various analyses. We conducted a careful examination, analyzing body size, shape, and morphological features, focusing on skeletogenesis, teeth, and organs like the brain and gut at different stages. Our analysis yielded valuable insights into 3D morphological changes during *Xenopus*’ development, documenting details previously unrecorded. These datasets hold the solid potential for further morphological and morphometric analyses, including segmentation of hard and soft tissues.

**Conclusions:**

Our repository of micro-CT scans represents a significant resource that can enhance our understanding of *Xenopus*’ development and the associated morphological changes in the future. The widespread utility of this amphibian species, coupled with the exceptional quality of our scans, which encompass a comprehensive series of developmental stages, opens up extensive opportunities for their broader research application. Moreover, these scans can be used in virtual reality, 3D printing, and educational contexts, further expanding their value and impact.

## Graphical Abstract

**Graphical Abstract:**
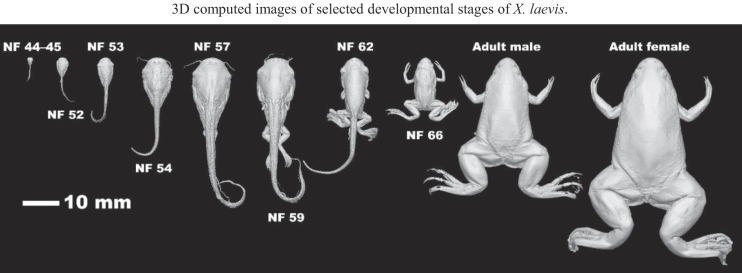
**Summary**: X-ray tomography was used to examine the African clawed frog (*Xenopus laevis*). This extensive dataset of specimens from tadpoles to adult frogs opens avenues to novel insights into the changes and developmental dynamics of selected structures, leading eventually to an improved understanding of this crucial animal model.

## Background

*Xenopus laevis* (NCBI:txid8355), commonly known as the African clawed frog, serves as a fundamental model organism in the field of life sciences. Its widespread adoption, particularly in biological research, can be attributed to the ease of breeding and housing, as well as the substantial size and manipulability of its eggs and embryos [1, 2]. Over the years, this species has been extensively investigated across various disciplines, including genetics, embryology, and developmental, cell, and regenerative biology [3–5]. While initial attempts at anatomical descriptions of *X. laevis* date back a century [6], subsequent studies have, to varying degrees, concentrated only on specific anatomical regions [7–25]. Despite these valuable contributions, many past approaches to describing frog anatomy failed to preserve the intricate morphological details, making them unsuitable for detailed and comparative studies. In response to these limitations, a detailed description of an adult male frog’s anatomy as a complete organism using a nondestructive micro–computed tomography (micro-CT) method was recently provided [7]. Micro-CT, to be candid, has already been employed to some extent as a valuable tool in selected stages of *X. laevis*’ embryos, tadpoles, and frogs to explore localized or specific occurrences, such as gastrulation [26], craniofacial [27] and urostyle [28] development, skeletal morphology [29, 30], limb (skeletal) regeneration [31–33], brain regeneration [34], and the anatomy of cranial and anterior spinal nerves [35], the head [36, 37], and several internal organs [38]. Besides *X. laevis* itself, micro-CT has also been utilized in closely related frogs to investigate, for instance, chondrocranium organization in *Alytes obstetricans* [39], skeleton [40] and digit [41] morphology in *Xenopus tropicalis*, cortical bone morphology in various anuran amphibians [42], and reevolution of lost mandibular teeth in *Gastrotheca guentheri* [43, 44], together with cranial musculoskeletal structures in *Pelobates fuscus* [45], lung rearrangements during metamorphosis in *Microhyla fissipes* [46], gill formation during metamorphosis in *Bufo bufo* [47], and metabolic reorganization in metamorphic *Rana omeimontis* tadpoles [48]. Nevertheless, the spatiotemporal dynamics of *X. laevis*’ late development at the level of a whole organism, along with the comparison between adult male and female frogs, remain largely unexplored in sufficient detail.

To fill this knowledge gap and gain a more comprehensive understanding of *X. laevis*’ late development at the level of a whole organism, we employed micro-CT, a powerful imaging technology for examining small objects at the micrometer scale [49, 50]. Micro-CT utilizes X-rays to generate high-resolution 3-dimensional (3D) images, making it an ideal tool for investigating developmental and morphological changes, particularly in embryos, tadpoles, and small animals [49, 50]. In principle, micro-CT imaging is based on capturing a series of 2-dimensional (2D) X-ray radiographs from various angles and then mathematically processing them through tomographic reconstruction, resulting in a 3D matrix representing volume density. An advantage of micro-CT lies in its ability to image bones and, when combined with various contrast methods, also soft tissues and blood vessels within the same sample [49, 50].

Through micro-CT, we present high-resolution images and computed 3D models of developing *X. laevis* tadpoles, froglets, and adult frogs, which offer great potential for research fields like developmental and comparative biology. Additionally, we conducted preliminary analyses of this dataset to illustrate its versatility. Specifically, our preliminary examination covered morphological changes in body size, shape, and skeletogenesis from selected premetamorphosis, prometamorphosis, and climax metamorphosis stages through the froglet stage up to adulthood. Our findings unveil promising and opening insights into the intricate morphological dynamics of late *X. laevis*’ development. To sum up, this research contributes a unique dataset concerning the developmental and morphological changes in *X. laevis*, including the adult stages, shedding light on the dynamics of vertebrate development and bearing broader implications for future analyses of *Xenopus*.

## Data Description/Sampling Strategy

In our micro-CT study of *Xenopus*, we employed a careful sampling strategy to encompass the entire developmental spectrum of this amphibian species. The selection of developmental stages was based on specific morphological features and markers that serve as critical indicators of *X. laevis*’ development (see below). By strategically choosing 9 distinct developmental stages, we aimed to capture the comprehensive process of morphogenesis, spanning from premetamorphosis tadpoles to fully mature adult frogs. These stages were carefully chosen with reference to specific criteria, such as the presence and dimensions of legs and the tail, or the rearrangements of the head, as outlined in the tables of Nieuwkoop and Faber [51] and Zahn and colleagues [52].

Our sampling included the following crucial stages of *X. laevis*’ development:

**Premetamorphosis** (stages 44–45, 52, and 53, according to Nieuwkoop and Faber, NF): These early stages provide insight into the initial stages of development on metamorphosis, as *Xenopus* transforms from tadpole to froglets.

**Prometamorphosis** (NF stages 54 and 57): This phase represents an intermediate stage where significant changes in the head, limb buds, and tail are occurring, leading slowly toward the climax metamorphosis.

**Climax metamorphosis** (NF stages 59 and 62): These stages are characterized by the most profound changes, marking the peak of metamorphosis.

**Froglets** (NF stage 66): Froglets represent a transitional stage, bridging the gap between tadpoles and fully mature adult frogs.

**Adult male and female frogs**: These adult stages represent the endpoint of *Xenopus* development, allowing us to observe fully mature individuals and to compare differences between sexes. We chose organisms that were approximately 1 year old for this micro-CT study.

Our selection of these key developmental stages was performed with regard to their significance in the overall developmental process of *X. laevis*. By examining these specific stages, we aimed to gain a solid dataset, enabling a comprehensive understanding of the morphological transformations and developmental milestones that occur during *X. laevis*’ development, from its primary aquatic tadpole phase to its secondary aquatic adult frog stage [53]. This sampling strategy provided a detailed and holistic view of *X. laevis*’ late development, enabling us to perform valuable analyses leading to preliminary insights and conclusions about this species’ morphological changes throughout its life cycle.

## Methods/Source of Samples

*Xenopus* embryos were generated and cultivated following standard protocols. Briefly, testes were surgically removed from anesthetized males (20% MS-222, Sigma-Aldrich, A5040) and transferred to cold 1× Marc’s Modified Ringers (MMR; 100 mM NaCl, 2 mM KCl, 1 mM MgSO_4_, 2 mM CaCl_2_, 5 mM HEPES, buffered to pH 7.4), supplemented with 50 µg/mL gentamycin (Sigma-Aldrich, G3632). To induce egg laying, fully mature *Xenopus* females were injected with 260 U of human chorionic gonadotropin (Merck, Ovitrelle 250 G) into the dorsal lymph sac approximately 12 to 16 hours before use and were kept overnight at 18°C. For fertilization, eggs were extracted from induced females directly into a Petri dish and mixed with a piece of testes in 0.1× MMR at 18–21°C. The subsequent development of *Xenopus* tadpoles, froglets, and adult frogs adhered to the approved cultivation protocols (see above). The *Xenopus* froglets and adult frogs were reared in XenopLus (catalog number RE18001301, Tecniplast), which is an advanced, fully automated system tailored for housing amphibians, guaranteeing excellent animal care and housing conditions. At a designated time point, *Xenopus* specimens were anesthetized and fixed in a buffered 4% paraformaldehyde solution (1004965000, Merck) for 3 hours (tadpoles) or overnight (froglets and adult frogs) and staged according to the tables of Nieuwkoop and Faber [51] and Zahn and colleagues [52]. Photographs of selected *Xenopus* specimens used for micro-CT analysis are presented in Supplementary Fig. S1.

## Methods/Micro-CT Scanning

Prior to scanning, samples were placed in either a 2-mL Eppendorf tube, a 15-mL/50-mL Falcon tube (depending on sample size), or, in the case of the adult frogs, a 500-mL plastic container. To prevent the motion and drying of the sample during the micro-CT scan, all samples were mounted in a 1% agarose gel (Top-BIO, P045). Micro-CT measurements were conducted using the GE Phoenix v|tome|x L 240 laboratory system, which is equipped with a 180 kV/15 W nanofocus X-ray tube and a 4,000-pixel × 4,000-pixel flat panel detector with a 100-µm pixel size. Scan conditions for each stage are summarized in Supplementary Table S1. It is important to note that the X-ray source target utilized in this study was tungsten. The subsequent tomographic reconstruction was performed using the GE Phoenix datos|x 2.0 software. The reconstructed data were imported into VG Studio MAX 2023.4 software (Volume Graphics GmbH), in which the measured data were segmented, analyzed, and visualized using VG Studio MAX (RRID:SCR_017997). The segmentation of *Xenopus* guts was done manually by an operator using the software Avizo 2020.2 (Thermo Fisher ScientificA) (Avizo 3D Software, RRID:SCR_014431).

All *Xenopus* samples were initially scanned in their native state to visualize bone structures. Subsequently, specimens were stained with 1% iodine (Penta, 21210–11000) in a 90% methanol (Penta, 17570–30250) solution to enhance the contrast and visualize soft tissues. As for teeth (Fig. 3), the separated jaws were scanned and stained. The dehydration and staining times for each *Xenopus* developmental stage are detailed in Supplementary Table S2.

## Data Quality Control and Limitations

**Figure 1:**
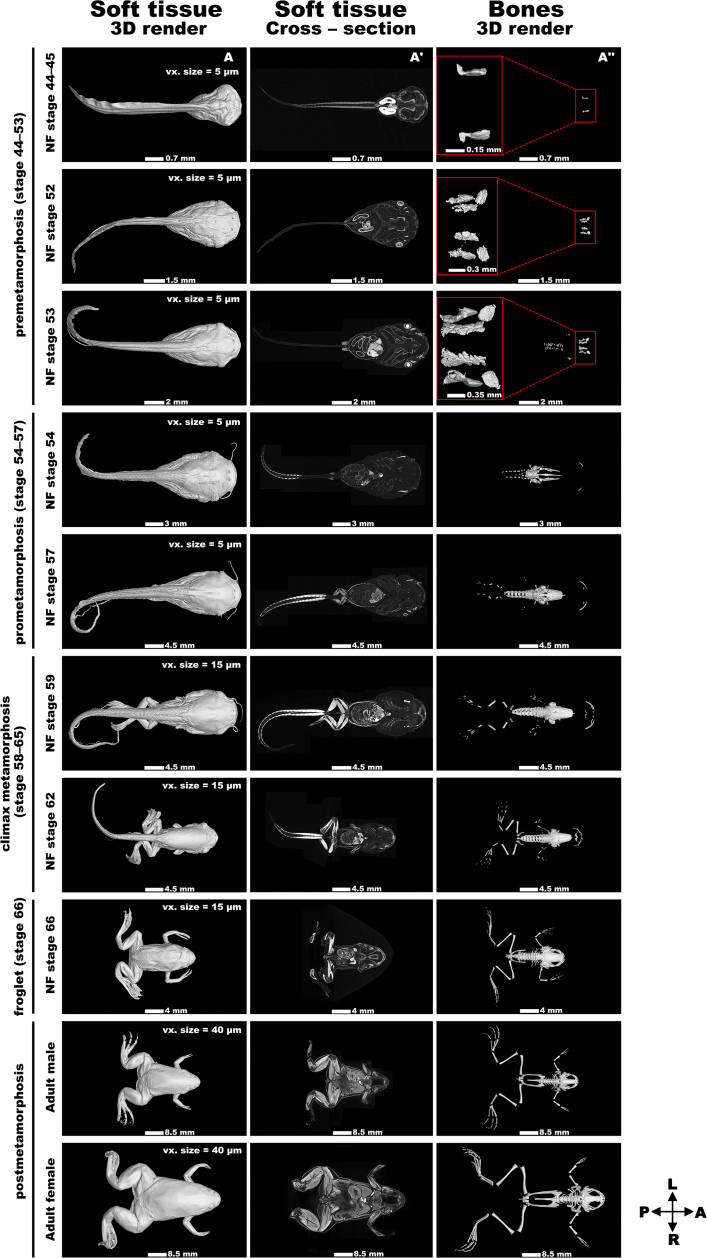
The atlas of *Xenopus laevis*’ late development. (A) 3D renders of soft tissues of selected *Xenopus* developmental NF stages. (A′) Cross sections of soft tissues. The section level was selected based on capturing the brain area of *Xenopus* specimens. (A′′) 3D renders of the skeleton. In the first 3 conditions (NF 44–53), the bones are zoomed in and shown in the red rectangle on the left. The relevant scale bar and voxel size are depicted on bottom or top of each picture. All views are from dorsal view with the cranial side pointing to the right. A: anterior; L: left; P: posterior; R: right.

All analyzed samples, comprising *X. laevis* tadpoles, froglets, and adult frogs, were uniformly preserved and handled before scanning. The primary variable affecting data quality was the varied voxel size of each dataset, arising from different sample sizes. The smallest sample, a tadpole stage (NF 44–45), featured a voxel size of 5 µm, while the largest sample, an adult female frog, was scanned with a voxel size of 40 µm (for a voxel size of all scanned NF stages, see the appropriate column in Supplementary Table S1 or Fig. 1).

The voxel size difference was directly correlated with sample dimensions. The GE L240 X-ray source’s cone beam geometry and the detector’s field of view allowed smaller samples to be placed closer to the X-ray source, resulting in a smaller voxel size (higher resolution) due to cone beam magnification. Although samples exhibited variable voxel sizes, this variability does not necessarily limit data analysis. Larger samples, despite having larger voxel sizes, still enabled the recognition of structures analyzed, as these structures were proportionately larger in larger animals scanned with larger voxel sizes.

All *Xenopus* specimens underwent 2 sequential scans. Initially, native scans were performed, following the principle of micro-CT imaging of samples in their native form without staining, enabling the visualization of dense mineralized structures like bones and teeth. Subsequently, all scans were stained as described in Supplementary Table S2. To prevent movement and drying of samples during data acquisition, all samples were fixed in polypropylene conical tubes with 1% agarose gel. Throughout data acquisition, no observable sample shrinkage occurred. If any negligible shrinkage or drying did occur, we anticipate the volume reduction to be proportionally consistent across all *Xenopus* samples.

Image data inherently exhibit limitations in terms of useful resolution and contrast among components. These constraints are integral to the imaging process and are not indicative of weaknesses in our study but rather intrinsic characteristics of the imaging modality employed. It is essential to acknowledge that, in any imaging technique, there are practical boundaries to the level of detail and contrast achievable.

## Atlas of *X. laevis*’ Late Development

In order to provide a more comprehensive understanding of *X. laevis*’ development during metamorphosis and organogenesis, we first collected specimens representing the key frog stages, including premetamorphosis, prometamorphosis, climax metamorphosis, froglets, and adult male and female frogs (see Sampling Strategy). Initially, we performed native sample scanning for hard tissue visualization and subsequently stained and scanned the samples for soft tissue visualization (see Micro-CT Scanning). As a result, we were able to collect and analyze all major stages of *X. laevis*’ late development. The entire collection of the *Xenopus* atlas is presented in Fig. 1, and all raw data can be found in the GigaDB repository (see the Data Availability section) [54]. Subsequently, with this atlas, we provide here several examples of specific analyses of *X. laevis*’ late development, including an analysis of head development, teeth, long bone growth dynamics, ossification, brain development with its associated nerves, and gut morphology.

## Head Development Analysis

**Figure 2:**
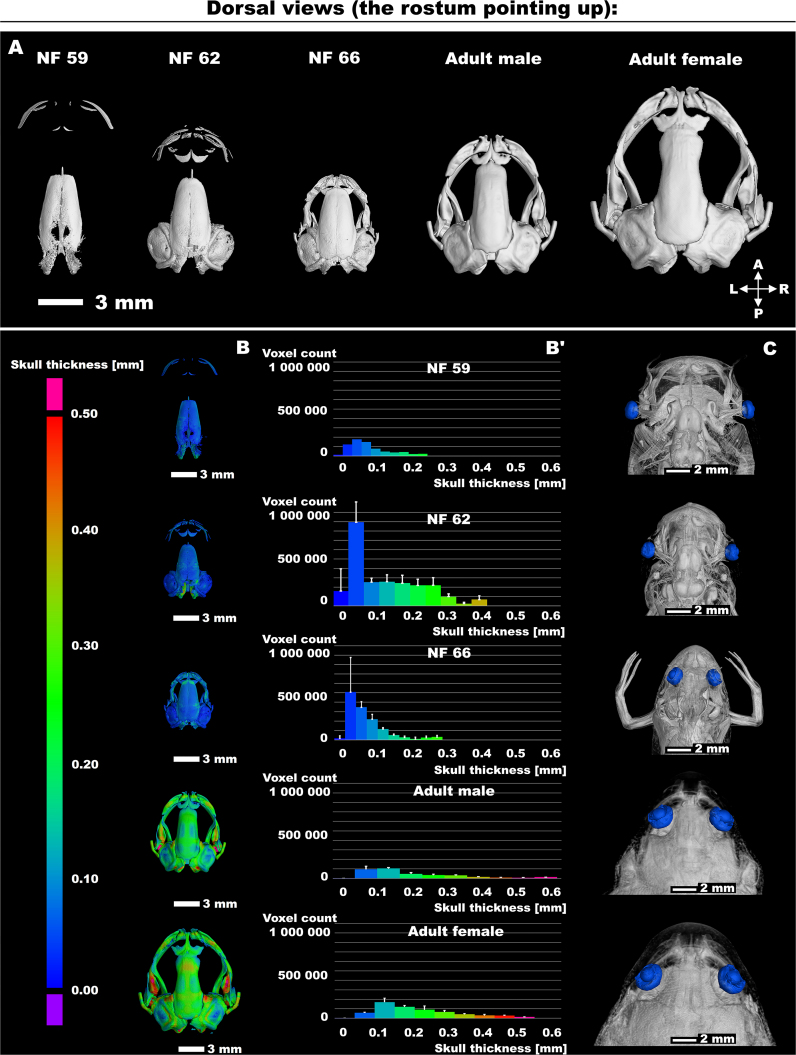
The analyses of the head development. (A) The series of *Xenopus* skulls of selected stages. The scale bar with values in mm is shown on the left bottom. (B) The skull thickness of selected stages is displayed. The scale bar with values in mm is shown on the left side. (B′) The relative distribution of bone thickness for each skull is shown. The experiment involved conducting 3 repetitions for each developmental stage, with the results depicted as means accompanied by standard deviations (± SD). (C) The overview of developing heads with highlighted eyes is shown.

First, we delved into the intriguing process of head development and skull metamorphosis in climax metamorphosis tadpoles, froglets, and adult frogs (refer to Fig. 2A; Supplementary Video S1). Our initial investigation employed morphometric analysis to shed light on the developmental changes in the skull. All skulls were oriented in the same direction (the anterior skull part heading to the top of a page; A: anterior; P: posterior; R: right; and L: left) in a top-bottom view. Such images can be further used, for example, to evaluate how the individual calvarian bones are being rearranged during metamorphosis or, for example, to shed light on facial region development.

Next, we performed the examination of skull thickness (Fig. 2B–B′; Supplementary Video S2). This analysis revealed a nonlinear relationship, with adult frogs possessing significantly thicker skulls compared to their younger counterparts. This investigation also suggests a positive correlation between skull mass and skull thickness.

Moving on, we made an introductory analysis regarding the eye distance during development, as illustrated in Fig. 2C. Our morphometric analysis (for an example of the measurement, see Supplementary Fig. S2A) revealed a progressive decrease in eye distance during developmental stages (roughly estimated in Supplementary Fig. S2B–B′). These changes actually occurred in *Xenopus* in 2 phases: a gradual increase in the premetamorphic stages (data not shown), followed by a peak at the onset of climax metamorphosis (NF stages 59 and 62), and finally, a decrease to a compact head configuration in froglets (NF stage 66) and adult frogs (Fig. 2C and very roughly estimated in Supplementary Fig. S2B–B′). This adaptation aligns well with the frog’s life strategy, transitioning from a water-dwelling tadpole with lateral eyes to an adult with eyes positioned on top of the head for a submerged lifestyle [55], reminiscent of crocodilians [56].

Based on the previous paragraphs, one can also explore the conservation analysis of the eye/head ratio with respect to frog sex. Findings of ours (and others [57]) indicate that *Xenopus* female heads exhibit an increase in volume compared to males (Fig. 3A, B). However, although our effort was in no way directive, the eye distance in females does not remain at 100% of the head volume in males, but it is relatively less (for a preliminary estimate, see Supplementary Table S3). This observation, which can be even assessed also from the macroscopic scale, may be linked to visual perception requirements, as the proximity of the eyes is still crucial for certain aspects of vision [58]. However, our intention here was just to demonstrate that similar and precise analyses are feasible with micro-CT data. In summary, our micro-CT dataset can be further employed for the study of the head and its associated organs such as the eyes in *X. laevis*. For more information about the *X. laevis*’ head morphology in general, we also refer to the handmade drawings by Zahn and colleagues [52].

Figure 3:Demonstrative interactive SketchFab visualizations of 3D reconstructions highlighting that *Xenopus* female heads exhibit an increase in volume compared to males. (A) Skull of *Xenopus laevis* adult female (link: https://sketchfab.com/3d-models/skull-of-xenopus-laevis-female-321240df5a5741d39937f65d457c1594). (B) Skull of *Xenopus laevis* adult male (link: https://sketchfab.com/3d-models/skull-of-xenopus-laevis-male-192dbbceb71f4b73a1097a7cdf67e5ae).
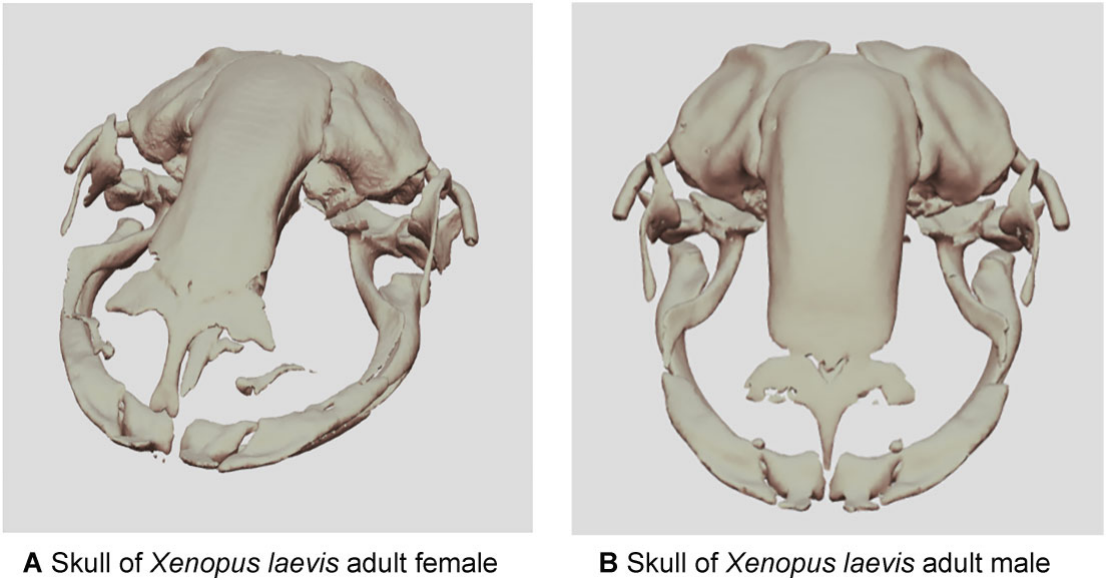


## Tooth Analysis

**Figure 4:**
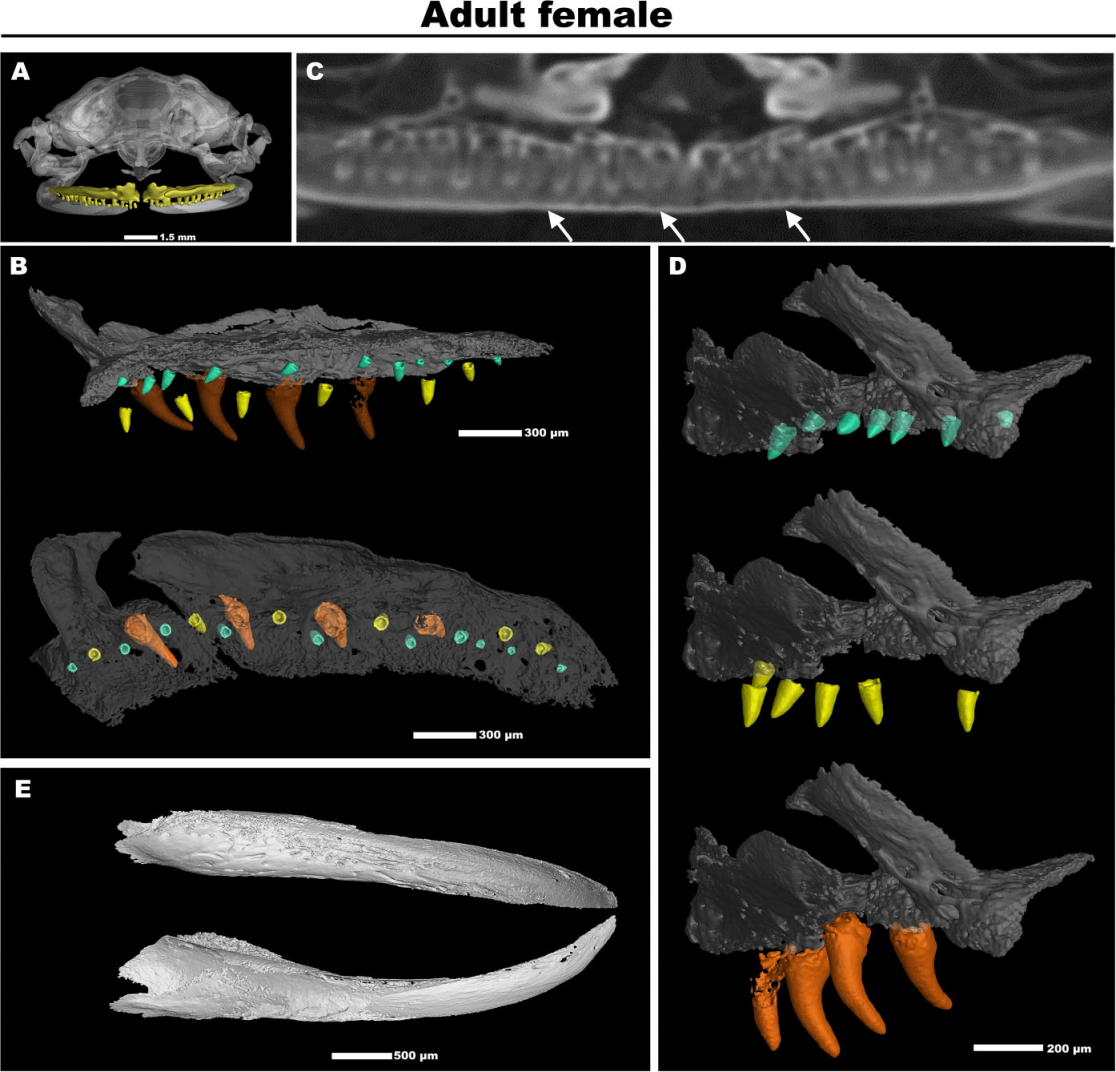
The analysis of teeth in an adult frog. (A) The frontal view of an adult female frog skull is depicted with an upper maxillary arch with maxillary teeth visualized in yellow. (B) The lateral and top view on the right half of the maxillary arch with visualized teeth from an adult female frog. Three different developmental stages of teeth are highlighted by different colors (yellow, cyan, orange). (C) The stained micro-CT scan shows that only a small portion of the tooth extends into the oral cavity. The arrows point to teeth rows that do not penetrate the oral cavity. (D) The lateral view of the mandible of an adult female frog confirms the absence of teeth in this area. (E) The lateral view of the rostral part of the maxilla displays different stages of teeth during the replacement of tooth rows in detail.

To enhance the depth of our analysis and increase its practical applicability, we conducted a comprehensive examination of the frog teeth, with an individual dataset of an adult female jaw. Upon initial observation, the presence of teeth in *X. laevis* may not be readily apparent. This is due to the fact that teeth were exclusively found in the maxilla, referred to as maxillary teeth (Fig. 4A, B) and behind the maxillary arch on the vomeral bone—so-called vomeral teeth (not shown). Additionally, only a small portion of the maxillary tooth protruded into the oral cavity [59] (Fig. 4C). In contrast, the mandible contains no teeth (Fig. 4D). The shape of maxillary teeth was generally uniform (i.e., homodont dentition), typically resembling simple conical structures (see Fig. 4B). *Xenopus*’ teeth exhibit an acrodont type of attachment, where they form an ankylotic attachment with the adjacent bone and are characteristic by regular renewal, a condition known as polyphyodont dentition. Notably, the phenomenon of tooth renewal in *Xenopus* is akin to what has been previously documented in other species, such as geckos [60, 61]. In agreement with this, we also noticed the evidence of the replacement in the maxillary teeth (Fig. 4E). These analyses revealed the progression of ankylosis, from the early development of teeth located closer to the gingiva to their full fusion with the adjacent bone (see Fig. 4E). Our *Xenopus* micro-CT dataset thus unveils the previously concealed dental structures of *X. laevis*, provides a high-resolution revelation of their “hidden” teeth patterning, and offers new perspectives for studying teeth and their growth in *Xenopus*.

## Micro-CT Data Analysis of Long Bone Growth Dynamics

**Figure 5:**
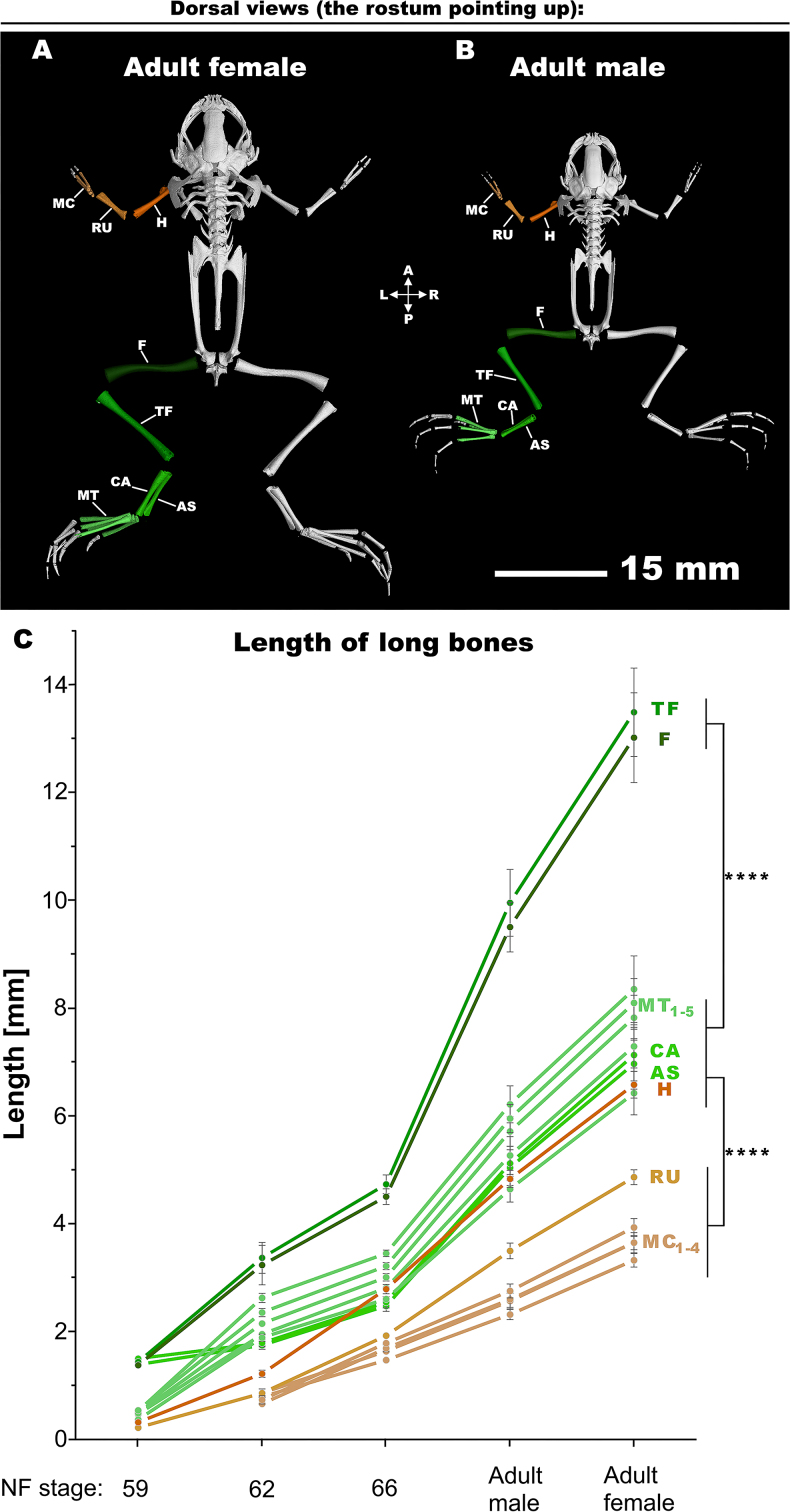
The analysis of frog skeletogenesis. (A) The adult male frog with analyzed bones is depicted. (B) The adult female frog with analyzed bones is displayed. (C) The graph demonstrating the analyzed bones throughout the *Xenopus laevis*’ late development. The experiment involved conducting 3 repetitions for each long bone measurement, with the results depicted as means accompanied by standard deviations (± SD). However, error bars are depicted only for cases demonstrating statistically significant differences, while they are not displayed for insignificant variations. Statistical analysis was performed using a 2-way analysis of variance (ANOVA) followed by Tukey post hoc test for multiple comparisons; ^****^P < 0.0001. A: anterior; AS: astragalus; CA: calcaneum; F: femur; H: humerus; L: left; MC: metacarpals; MT: metatarsals; P: posterior; R: right; RU: radioulna; TF: tibiofibular.

Next, we assessed the growth dynamics of several long bones (shown in adult frogs in Fig. 5A, B and Supplementary Video S3; refer to Supplementary Fig. S3 to see how the analysis was performed). Based on our micro-CT analysis, in terms of sex, we found that adult females can be viewed as essentially enlarged males, indicating a proportional growth pattern (Fig. 5C). We also investigated whether the development of the left and right sides of *X. laevis* is uniform or divergent (Supplementary Fig. S4), and consistently, we observed L-R length symmetry in the length of all long bones. Therefore, we next focused our detailed examination only on one side of the frog, specifically the left side (L).

Our findings revealed that certain limb bones, particularly those in the hindlegs, such as the femur and tibiofibular, exhibited relatively rapid growth, while other bones such as forelimb ones grew at a slower rate (Fig. 5C). Importantly, this difference was not attributed to absolute bone measurements but rather in relation to the animal’s overall length (data not shown). This phenomenon may be linked to the frogs’ utilization of their hindlimbs for swimming, whereas even their forelimbs are primarily employed for food handling [62, 63]. Moreover, the length of the tarsal bone, such as astragalus and calcaneum, appears to be less critical for escape or startle responses, such as swim kicking, compared to the lengths of the femur and tibiofibular, which are correlated with the muscle mass of the thigh and the calf [7]. It is also intriguing to note that most bones initiated their growth from roughly the same size but terminated it at different dimensions. For example, metatarsals develop at a faster rate than metacarpals (Fig. 5C), which aligns with functional considerations, as outlined elsewhere [37]. Together, based on our micro-CT data, one can make new predictions for further experimental testing of (long) bone growth.

## Ossification Analysis

Besides bone length, it is also feasible to analyze the bone mass using our *Xenopus* dataset. Within the frog skeleton, 2 distinct types of bones can be generally distinguished in terms of their developmental origin. Dermal ossification, originating in the dermis, is evident in certain skull elements and in 2 bones of the pectoral girdle, namely, the cleithrum and clavicle. Conversely, the remaining components of the postcranial skeleton consist of either cartilage or bones replacing the cartilage, achieved through endochondral ossification [19].

**Figure 6:**
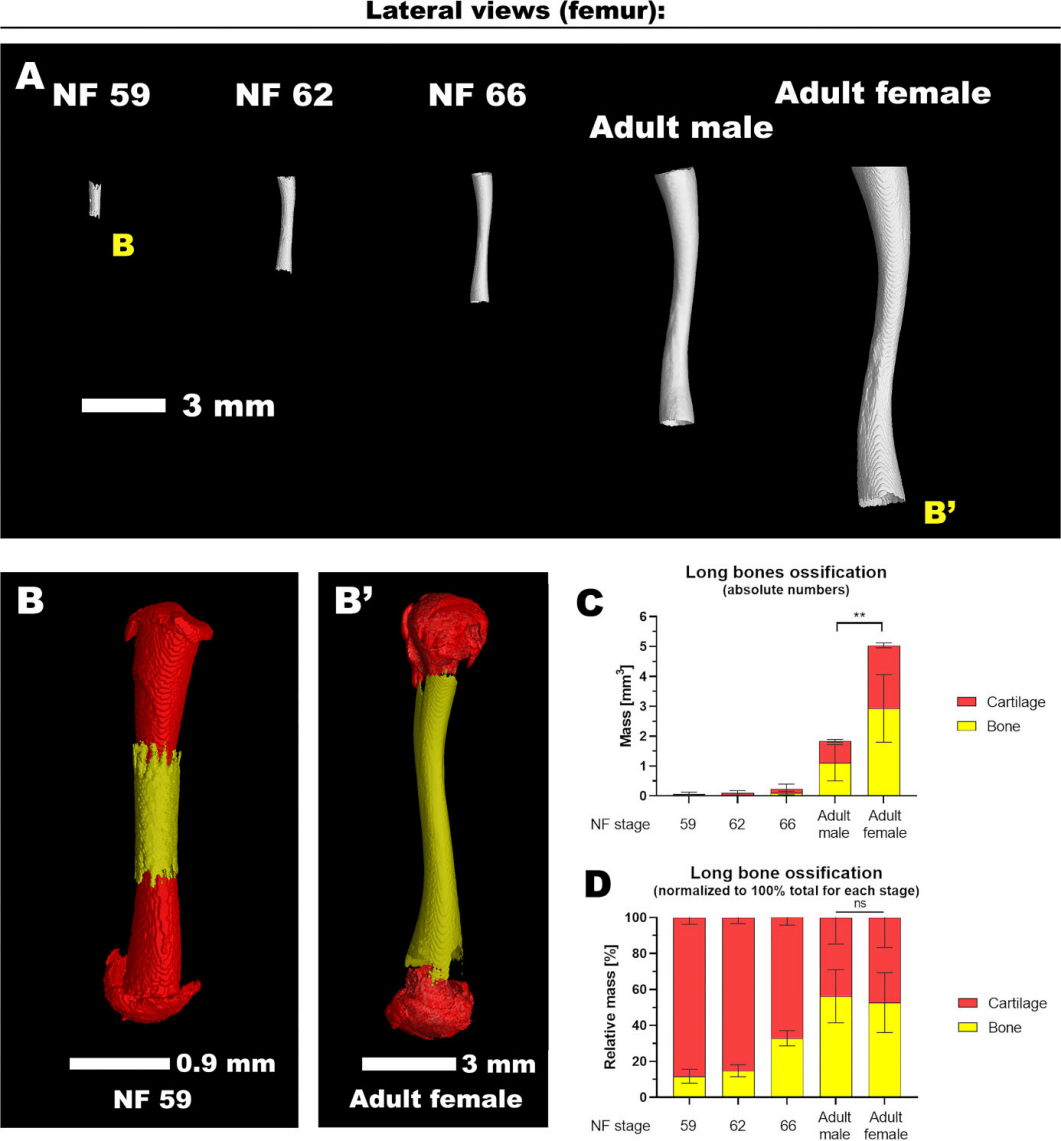
The analysis of long bones, including their cartilage and ossification. (A) The collection of femur bone throughout the late development. (B–B′) The femur from different developmental stages such as NF 59 (B) and an adult female (B′) with a focus on cartilage (in red) and bone (yellow). The view of bones is maximized and not in real proportions. For size proportions, see Fig. 5A. The absolute (in C) and relative (in D) quantification of a bone and cartilage mass for selected long bones such as the femur, humerus, and radioulna for each developmental stage, with the results depicted as means accompanied by standard deviations (± SD). Statistical analysis was performed using a 2-way analysis of variance (ANOVA) followed by Tukey post hoc test for multiple comparisons; **P < 0.01; ns, *P* > 0.05. Additionally, it should be noted that in (A), the cartilage is not visible, while in (B), the stained data allow for a clear separation of cartilage from bones. The merged data in Fig. 5B emphasize the isolated cartilage.

As the bones of the limbs undergo development in both tadpoles and adults, they typically comprise an ossified section alongside cartilage. Notably, the ossification process always commences at the central regions of long bones, as shown on the example of the femur (Fig. 6A). By assessment of the mass ratio between cartilage and bone (Fig. 6B–B′; Supplementary Fig. S5), we observed that, with a few exceptions, all long bones share relatively similar proportions (data not shown). Furthermore, in both adult males and females, there is a lack of dynamic growth akin to the developmental stages, with only a noticeable increase in mass (Fig. 6C). After climax metamorphosis, no proliferative ossification occurs, and the process is limited to the calcification of cartilage, irrespective of the frog’s sex (Fig. 6C). Moreover, the relative ratio of cartilage to an ossified bone diminishes as development progresses, exemplified by the femur, humerus, and radioulna (Fig. 6C, D). Thus, researchers can further take advantage of these indications to compare the growth of different bones in various aged animals.

## Segmentation of Selected Internal Soft Organs

Our dataset offers significant potential not only for the evaluation of hard bones but also for soft tissue such as internal organs. Precise segmentation of structures is essential because once the structure in focus is clearly distinguished, further assessment of its morphology and intraspecies differences becomes considerably easier and more accurate. This tool also provides the flexibility to describe each internal structure in detail either separately or in the context of its individual surrounding elements. In addition to studying differences during *X. laevis*’ development, segmented structures can be utilized (e.g., for investigating intersex differences). For an illustration of the possible application of our micro-CT data, here we further selected 2 key vertebrate organs, such as the brain and gut.

### The brain

**Figure 7:**
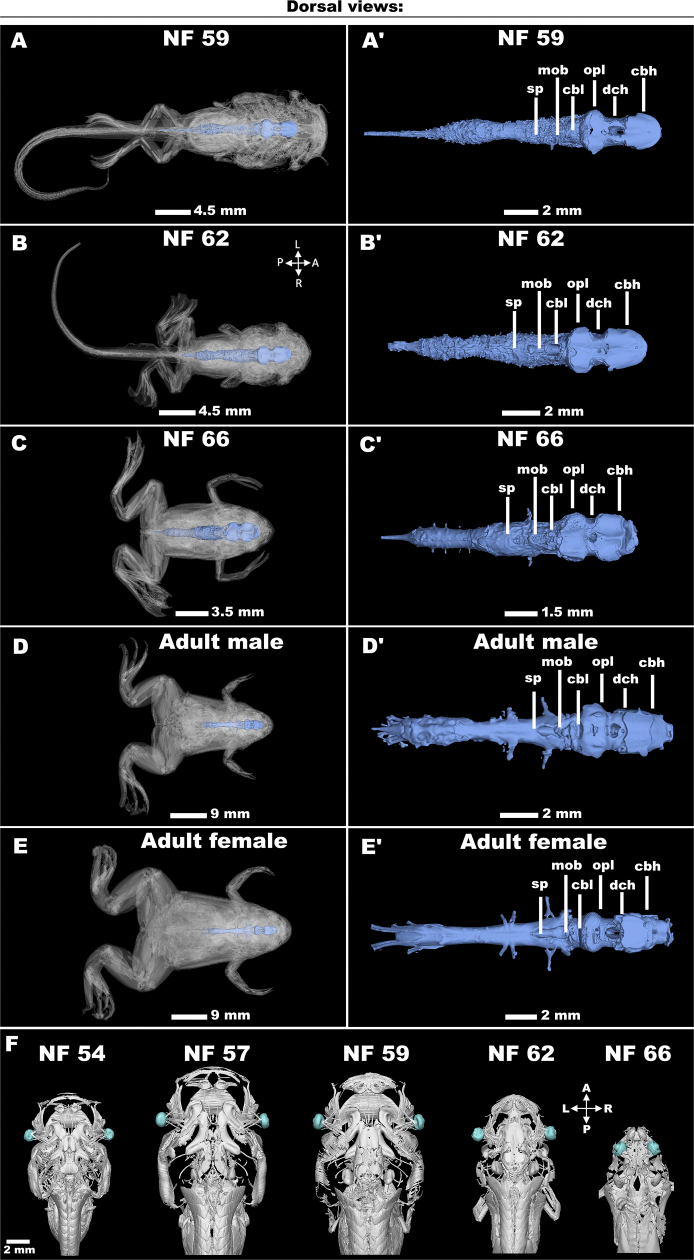
The analysis of brain development. (A–E) Individual stages of *Xenopus* brain and their details in (A′–E′), the rostrum pointing to the right, and (F) nervus opticus attached to eyes (in cyan), the rostrum is pointing up. cbh: cerebral hemispheres; cbl: cerebellum; dch: diencephalon; mob: medulla oblongata; opl: optic lobes; sp: spinal cord.

It is noteworthy that our micro-CT data allow for detailed observation of the developmental segmentation of various regions of the brain, which is very hard to dissect, especially from *Xenopus* adults (personal observation). Specifically, Fig. 7A–E, A′–E′, and Supplementary Video S4 provide a clear morphological visualization of the individual brain areas such as cerebral hemispheres (cbh), cerebellum (cbl), diencephalon (dch), medulla oblongata (mob), optic lobes (opl), and spinal cord (sp), in the tadpole and adult frog brains, as well as to follow how these structures are developing in time course. For more information about the *X. laevis*’ brain and its detailed description, including its regeneration in developing tadpoles, we refer readers to the recent publication [34]. Subsequently, we also asked whether we could analyze not only the brain but also the brain-associated nerves like *nervus opticus* using the micro-CT dataset (Fig. 7F). Indeed, we could see that the nerves attached to eyes slowly elongated and then modified, as a specimen size changed during development (Fig. 7F). Together, the quality of our micro-CT allows us to investigate not only the individual brain parts but also its associated structures.

### The gut

During the metamorphosis of *X. laevis*, the gut undergoes significant remodeling, with the intestine shortening by approximately 75% over an 8-day period. The coiling pattern changes, with the outer loops initially coiling counterclockwise and the inner loops coiling clockwise, reversing at the ileum’s switchback point [64, 65]. This remodeling is characterized by a stage-dependent sequential organization of nascent smooth muscle cells, which plays a crucial role in gut coiling morphogenesis [66].

**Figure 8:**
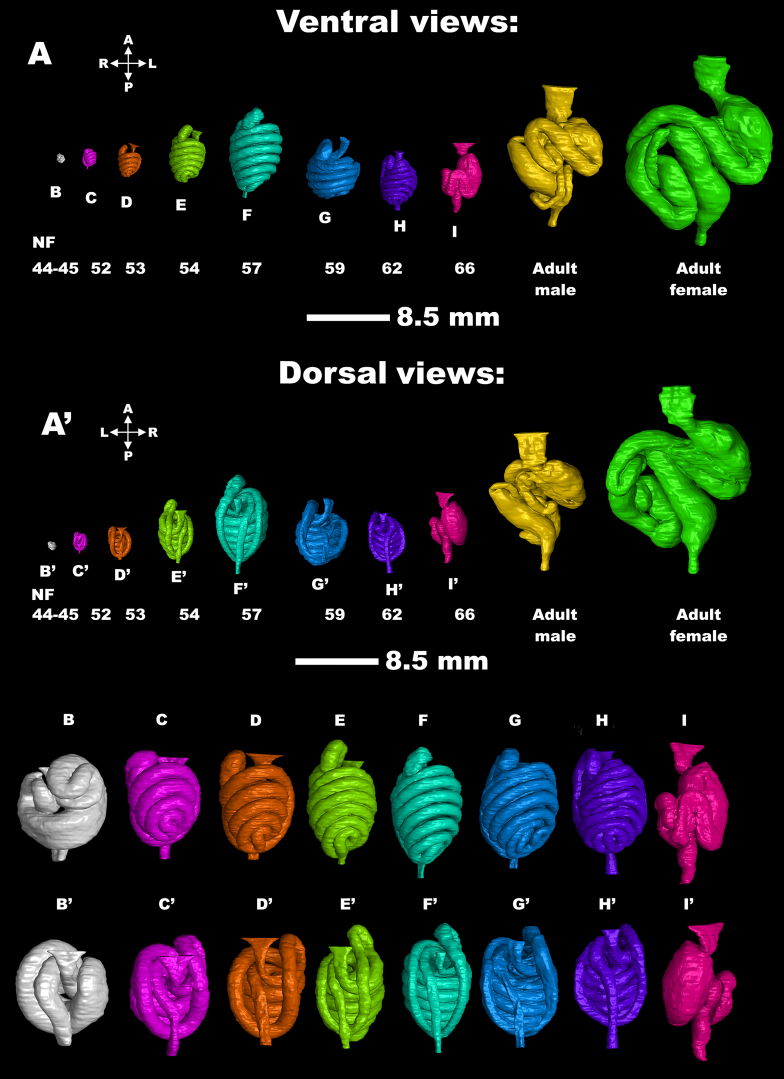
The analysis of gut development. (A–A′) Ventral and dorsal views of individual stages of *Xenopus* gut and their details for each developmental stage (B–I, B′–I′), except guts from frog adults.

To our best knowledge, this process has not been studied using micro-CT so far. Thus, we selected the gut as a second internal organ to dissect by the micro-CT technique, with a focus on the whole *X. laevis* atlas (Fig. 8). It is evident from the micro-CT images that it successfully depicted the gut structure with high quality and detail (Fig. 8 and Supplementary Video S5). For more information about the *X. laevis* gut and its anatomy in general, we refer a reader to the *Xenopus* illustrations by Zahn and colleagues [52] and supporting literature [64, 65].

In sum, researchers will be able to use our micro-CT dataset to segment and explore several internal organs, including the brain or gut, as well as associated structures such as nerves, for further analytical and practical purposes, as mentioned in Discussion below.

## Discussion

This study used micro-CT to create high-resolution 3D models of *X. laevis* from tadpoles to adults, revealing detailed developmental and morphological changes. The micro-CT scan repository enhances understanding of *X. laevis*’ development and has applications in research, virtual reality, 3D printing, and education.

The *X. laevis*’ development dataset not only offers valuable insights into this amphibian species but also presents a wealth of biological potential as it serves as a starting point for comparative, regenerative, and evolutionary biology as well as morphological study. The dataset’s diverse specimen representation and the high quality of the scans open up a realm of possibilities for advanced subsequent analyses. One intriguing avenue of study lies in the examination of hard tissue growth. The dataset provides an ideal platform to investigate especially the long bones of *Xenopus* but also vertebrae, or a skull. Furthermore, the dataset facilitates the analysis of teeth or cartilage, which we have addressed using dedicated tools in VG Studio Max.

Next, this dataset lends itself to in-depth exploration by researchers interested in the anatomy of *Xenopus*, including internal organs. The dataset’s segmentation capabilities enable the precise segmentation of individual structures, which can then be further processed to obtain detailed information regarding their shape, volume, and relationships with surrounding structures in 3 dimensions.

To enrich our understanding of adult *Xenopus* anatomy, morphology, and development, the dataset also includes scans of multiple *Xenopus* specimens. By combining these various analyses, the dataset offers comprehensive and detailed information on both hard and soft tissue morphology, making it a valuable resource for researchers studying this amphibian in the context of development.

Acknowledging the significance of our micro-CT data, it is also necessary to address the common limitations linked to the absence of 3D structural information about organs, such as the brain. The potential application of micro-CT, particularly in the study of mutants or diseased frogs, can offer valuable insights into the physical characteristics associated with developmental or neurological disorders. This not only is valid for *Xenopus* but also represents potential implications for humans. Notably, similar micro-CT–based studies involving, for instance, rat brains further underscore the relevance of our assumption [67]. Alternatively, the integration of the micro-CT technique with the CRISPR/Cas9 approach can be even employed for disease modeling, as demonstrated in the recent study involving *Xenopus* tadpoles [68].

In conclusion, this extensive dataset serves as a cornerstone for advancing our knowledge of amphibian morphological structures and evolutionary adaptations.

## Potential Implications

Our *Xenopus* micro-CT dataset can be processed to create 3D representations such as views and videos, showcasing various developmental stages, including frog adulthood. This presents significant educational and popularization potential, offering an innovative approach to understanding the complex the intricate process of *Xenopus* development.

In addition to providing 3D views and videos, the micro-CT dataset opens up exciting possibilities for 3D printing applications. Through micro-CT utilization, one can generate precise digital models of *Xenopus* tadpoles, froglets, adult frogs, and/or selected structures (see Supplementary Fig. S6 for an example of 3D print). These 3D-printed replicas offer interactive learning experiences, enabling both students and the general public to physically engage with the developmental stages of *X. laevis*. By handling a 3D-printed animal sample or examining the detailed structures of its internal organs, learners of all ages can deepen their understanding in a tangible and captivating manner.

Moreover, these 3D models can serve as valuable resources for engaging the general public, particularly in museum settings, without requiring original specimens or samples (or their organs) preserved in fixatives. This immersive approach not only enhances understanding of *Xenopus*’ development but also makes it accessible to a wider audience, fostering an appreciation for the intricacies of this fascinating organism.

## Supplementary Material

giae037_GIGA-D-23-00370_Original_Submission

giae037_GIGA-D-23-00370_Revision_1

giae037_Response_to_Reviewer_Comments_Original_Submission

giae037_Reviewer_1_Report_Original_Submission Brian Metscher -- 12/29/2023 Reviewed

giae037_Reviewer_2_Report_Original_Submission Virgilio Gail Ponferrada, Ph.D. -- 1/5/2024 Reviewed

giae037_Reviewer_2_Report_Revision_1 Virgilio Gail Ponferrada, Ph.D. -- 4/29/2024 Reviewed

giae037_Reviewer_3_Report_Original_Submission John Wallingford -- 1/10/2024 Reviewed

giae037_Supplementary_Video_1 The skull of an adult *Xenopus* female in 3D view.

giae037_Supplementary_Video_2 The skull of an adult *Xenopus* female in 3D view while using wall thickness analyses.

giae037_Supplementary_Video_3 The skeleton of an adult *Xenopus* female in 3D view.

giae037_Supplementary_Video_4 The brain of an adult *Xenopus* female in 3D view.

giae037_Supplementary_Video_5 The gut of an adult *Xenopus* female in 3D view.

## Data Availability

All volumetric data of the scanned *Xenopus* specimens can be accessed in the *GigaScience* repository, GigaDB [54]. These datasets are presented as already reconstructed 8-bit TIFF stacks. To enhance accessibility, the data were converted from their original 16-bit format to 8 bits, making them more compact for downloading and easier to open and analyze. This reduction in data size is particularly beneficial given the computational demands associated with working with large datasets. The provided videos showcase individual animals and highlight analyses conducted using the scanned data, as detailed in the supplementary files. Additionally, .STL files accompany the image stacks, enabling users to visually inspect the scanned animals in 3 dimensions. These .STL models are also accessible via the GigaDB repository [54]. While the deposited .TIFF format data can be viewed in any basic image viewer, for a comprehensive exploration of the 3D nature of the images and the analyses performed, it is recommended to use a dedicated 3D data viewer. VG Studio MAX, a commercial software package from Volume Graphics GmbH, offers a wide range of tools for visualizing, manipulating, and analyzing 3D micro-CT image data. Its freeware version, MyVGL, is a suitable alternative for visualizing all datasets and their corresponding analyses. Another free software option is the Fiji ImageJ distribution from the National Institutes of Health or Avizo software (ThermoFisher), both capable of opening individual image stacks and creating 3D renders of all scanned *Xenopus* specimens. In summary, the volumetric data of scanned animals, available on the GigaDB repository [54], provide efficient accessibility through 8-bit TIFF stacks, complemented by .STL models, with recommended 3D exploration using software like VG Studio MAX, MyVGL, Avizo, or Fiji ImageJ. Printable 3D models are additionally available from SketchFab and Thingiverse. The SketchFab collections are as follows: *Xenopus* development: https://sketchfab.com/GigaDB/collections/xenopus-laevis-development-3922d82fde27407ea1e7cc4622376178. *Xenopus* adult male: https://sketchfab.com/GigaDB/collections/xenopus-laevis-male-7eb069e65d2f49749ce4b4144dd5fc81. *Xenopus* adult female: https://sketchfab.com/GigaDB/collections/xenopus-laevis-female-b33299ca19604663a0cbdeb915f683e9. The Thingiverse links for the collection of printable models is as follows: https://www.thingiverse.com/thing:6620040.

## Abbreviations

A: anterior; AS: astragalus; CA: calcaneum; cbh: cerebral hemispheres; cbl: cerebellum; dch: diencephalon; F: femur; H: humerus; L: left; MC: metacarpals; micro-CT: micro–computed tomography; MMR: Marc’s Modified Ringers; MT: metatarsals; mob: medulla oblongata; NF stage: Nieuwkoop and Faber stage; opl: optic lobes; P: posterior; R: right; RU: radioulna; sp: spinal cord; SS: suprascapular; TF: tibiofibular.
