# Supplementary material for: Unveiling vertebrate development dynamics in frog Xenopus laevis using micro-CT imaging
Source: Gigascience. 2024 Jul 17;13:giae037. doi: 10.1093/gigascience/giae037 (PMC12558079; doi:10.1093/gigascience/giae037)
Supplement: giae037_GIGA-D-23-00370_Revision_1 [file giae037_GIGA-D-23-00370_Revision_1.pdf]

GigaScience

Unveiling Vertebrate Development Dynamics in Frog Xenopus laevis using Micro-CT Imaging

--Manuscript Draft--

**Manuscript Number:**	GIGA-D-23-00370R1
**Full Title:**	Unveiling Vertebrate Development Dynamics in Frog Xenopus laevis using Micro-CT Imaging
**Article Type:**	Research
**Funding Information:**	CzechNanoLab Research Infrastructure (LM2023051) — Not applicable<br>Brno University of Technology (FSI-S-23-8389) — Dr. Jozef Kaiser<br>Grantová Agentura České Republiky (GA22-02794S) — Dr. Jan Krivanek, Dr. Marcela Buchtova<br>Grantová Agentura České Republiky (GA22-06405S) — Dr. Jakub Harnos<br>Grant Agency of Masaryk University (MUNI/J/0004/2021) — Dr. Jakub Harnos<br>Ministerstvo Školství, Mládeže a Tělovýchovy (CZ.02.01.01/00/22_008/0004634) — Dr. Jozef Kaiser

Funding Information:	
CzechNanoLab Research Infrastructure (LM2023051)	Not applicable
Brno University of Technology (FSI-S-23-8389)	Dr. Jozef Kaiser
Grantová Agentura České Republiky (GA22-02794S)	Dr. Jan Krivanek<br>Dr. Marcela Buchtova
Grantová Agentura České Republiky (GA22-06405S)	Dr. Jakub Harnos
Grant Agency of Masaryk University (MUNI/J/0004/2021)	Dr. Jakub Harnos
Ministerstvo Školství, Mládeže a Tělovýchovy (CZ.02.01.01/00/22_008/0004634)	Dr. Jozef Kaiser

Abstract:

Abstract

Background:

Xenopus laevis, the African clawed frog, is a versatile vertebrate model organism employed across various biological disciplines, prominently in developmental biology to elucidate the intricate processes underpinning body plan reorganization during metamorphosis. Despite its widespread utility, a notable gap exists in the availability of comprehensive datasets encompassing Xenopus' late developmental stages.

Findings:

In the present study, we harnessed micro-computed tomography (micro-CT), a non-invasive 3D imaging technique utilizing X-rays to examine structures at a micrometer scale, to investigate the developmental dynamics and morphological changes of this crucial vertebrate model. Our approach involved generating high-resolution images and computed 3D models of developing Xenopus specimens, spanning from premetamorphosis tadpoles to fully mature adult frogs. This extensive dataset enhances our understanding of vertebrate development and is adaptable for various analyses. For instance, we conducted a careful examination, analyzing body size, shape, and morphological features, with a specific emphasis on skeletogenesis, teeth, and organs like the brain and gut at different stages. Our analysis yielded valuable insights into the morphological changes and structure dynamics in 3D space during Xenopus' development, some of which were not previously documented in such meticulous detail. This implies that our datasets effectively capture and thoroughly examine Xenopus specimens. Thus, these datasets hold the solid potential for additional morphological and morphometric analyses, including individual segmentation of both hard and soft tissue elements within Xenopus.

Conclusions:

Our repository of micro-CT scans represents a significant resource that can enhance our understanding of Xenopus' development and the associated morphological changes in the future. The widespread utility of this amphibian species, coupled with the exceptional quality of our scans, which encompass a comprehensive series of developmental stages, opens up extensive opportunities for their broader research application. Moreover, these scans have the potential for usage in virtual reality, 3D printing, and educational contexts, further expanding their value and impact.

Corresponding Author:

Jakub Harnos
Masaryk University: Masarykova Univerzita
Brno, CZECH REPUBLIC

Powered by Editorial Manager® and ProduXion Manager® from Aries Systems Corporation

**Corresponding Author Secondary Information:**	
**Corresponding Author's Institution:**	Masaryk University: Masarykova Univerzita
**Corresponding Author's Secondary Institution:**	
**First Author:**	Jakub Laznovsky
**First Author Secondary Information:**	
**Order of Authors:**	Jakub Laznovsky
	Michaela Kavkova
	Alice Reis
	Pavla Robovska-Havelkova
	Lorena Agostini Maia
	Jan Krivanek
	Tomas Zikmund
	Jozef Kaiser
	Marcela Buchtova
	Jakub Harnos
**Order of Authors Secondary Information:**	
**Response to Reviewers:**	Our responses have been upload as a separate document. We are enclosing the text here:<br><br><br><br>Reviewer #1:<br>The authors present a set of 3D images of selected developmental stages of the widely-used laboratory model Xenopus laevis along with some examples of how the data might be used in developmental analyses. The dataset covers stages from mid-larva through metamorphosis to adult, which should provide a starting point for various studies of morphological development. Some studies will undoubtedly require other stages or more detailed images, but the presented data were collected with straightforward methods that will allow compatibility with future work.<br><br>The data appear to be sound in the collection and curation. Data availability is made clear in the article, and the complete set will be publicly available in standard formats on the Zenodo repository. This should ensure full accessibility to anyone interested.<br><br>The article is well-organized and clearly written.<br>We'd like to express our gratitude to Reviewer #1 for dedicating the time and effort to reviewing our manuscript.<br><br>A few points about the methods could be clarified:<br>Was only one specimen per stage scanned?<br>Initially, we conducted a single repetition merely to demonstrate the possible analyses without intending to infer any significant biological relevance from it. However, based on the feedback from other reviewers and the editor, we increased the sample size to n=3, thereby reinforcing the validity of our initial suggestions. We trust that this addresses the concerns raised by Reviewer #1.<br><br>Specimens were dehydrated through an ethanol series and then stained with free iodine in 90% methanol, and then rehydrated back through ethanol. Why was methanol used for staining and not dehydration? It seems odd to switch alcohols back and forth without intermediate steps. This could have some effect on tissue shrinkage.<br><br>We appreciate the feedback regarding our choice of ethanol for dehydration and

methanol as a solvent for iodine in our protocol. The decision to use ethanol was based on its non-toxic nature, allowing for safer handling on the bench and also, we have adapted this dehydration from commonly used histologic methods used for the processing of animal tissue samples. The reason behind using methanol as a solvent for the iodine staining solution is that methanol has a smaller molecule than ethanol and thus the staining solution based on methanol better permeates the tissue. This method is commonly used in our collaborating research groups with great results (see the DOIs below this comment). We hope this clarification addresses any concerns and is satisfactory for the review process.
doi.org/10.1038/s41598-021-84579-x
doi.org/10.1088/1748-0221/13/02/C02039
doi.org/10.26508/lsa.202302073
doi.org/10.1038/s41467-023-39373-w

It should be indicated that the X-ray source target is tungsten (even though it is unlikely to be anything else in this machine).

Yes, we added this information into the manuscript, as requested (p. 8):
It is important to note that the X-ray source target utilized in this study was tungsten.

The "real images" (p. 7) in Suppl. Fig. 1 should simply be called photographs - microCT images are real too.
Yes, we changed this information in the manuscript, as suggested (p. 7).

For the measurements of bone mass, is the cartilage itself actually visible in the microCT images?

We only observed the cartilage in stained data. It's worth noting that Fig. 5A is derived from non-stained data, while Fig. 5B is from stained data. Furthermore, in Fig. 5B, we separated the cartilage from bones, and this represents the merged data. We have included this description in the figure legend:
Additionally, it should be noted that in A), the cartilage is not visible, while in B), the stained data allows for clear separation of cartilage from bones. The merged data in Fig. 5B) emphasizes the isolated cartilage.
Also, we put some additional image regarding this matter into the Supplementary material, specifically Suppl. Fig. 5.

p. 13: "The dataset's diverse species representation…" What does this mean? It is only one species.

We thank the reviewer for this note. We apologize for this typo and correct the sentence to: "The dataset's diverse specimens' representation…"(p. 14); ("diverse" in this context means tadpoles, froglet, adult frogs)

The limitations on the image data are not discussed. All images have limits to their useful resolution and contrast among components; this is not a weakness, just a reality of imaging.

We acknowledge the reviewer's insightful comment regarding the limitations on the image data. In order to answer this point, we added the following paragraph to our manuscript (p. 9):
Image data inherently exhibits limitations in terms of useful resolution and contrast among components. These constraints are integral to the imaging process and are not indicative of weaknesses in our study, but rather intrinsic characteristics of the imaging modality employed. It is essential to acknowledge that, in any imaging technique, there are practical boundaries to the level of detail and contrast achievable.

The different reconstructed voxel sizes for different size specimens are mentioned, but it might be helpful to indicate the voxel sizes in Figure 1 as well as in the relevant table.
The table containing voxel size details for each NF stage is provided in the manuscript as Supplementary Table 1. Furthermore, we acknowledge the omission of the link in the text. To address this, we have added the link to Suppl. Table 1 into the text (p. 7). Additionally, we have included the voxel size values in Figure 1 for further clarity.

Powered by Editorial Manager® and ProduXion Manager® from Aries Systems Corporation

And if the middle column of Figure 1 could be published with full resolution of the snapshots it would help show the actual quality of the images.
As part of the revision process, all figures have been uploaded as individual high-quality files. Consequently, all figures now have sufficient resolution.

Reviewer #2:
The manuscript is well written and easy to understand. It will be a good contribution to the Xenopus research community as well as a useful reference for the field of developmental and amphibian biology.
We want to extend our appreciation to Reviewer #2 for the time and effort invested in reviewing our manuscript.

I suggest the following revisions:
- For the graphical abstract try alternating NF stage numbers above and below samples for a cleaner look, adult male and adult female can both remain at the top.
We appreciate the reviewer's suggestion and have incorporated this comment into the graphical abstract.

- Appreciate the rationale for providing the microCT analysis presented in this manuscript and choices of late-stage tadpoles, pre- and prometamorphosis, through metamporphosis to the adult male and female frog.
Thank you, we're grateful for it.

- For the head development section authors can make reference to the Xenhead drawings, Zahn et al. Development 2017.
In the mentioned section, we included the following statement (p. 11):
For more information about the X. laevis' head morphology, we also refer to the handmade drawings by Zahn and colleagues (Zahn, James-Zorn et al. 2022).

- Head Development section paragraph 4, change word from "gender" to "sex."
Certainly, we've updated this information in the manuscript as advised (page 9).

- Supplementary Table 3. Change "gender-related" to "sex-related."
Yes, we've made the suggested change in the manuscript (p. 28).
- Micro-CT Data Analysis of Long Bone Growth Dynamics section paragraph 1 change "in terms of gender" to "in terms of sex."
We have made the necessary adjustment in the manuscript as advised (p. 10).

- Figure 4 panels A and B don't reflect the observation that adult females are enlarged males. While the authors state that the view of the male and female skeletons are maximized and not proportional as stated in the caption, suggest that scale bars be employed and the images adjusted to show the size relationship difference between the sexes as in Figure 1. On first glance and perhaps to those not as familiar with the difference in sex size in Xenopus that in this particular example of the adult male image being more spread out compared to the image of the female, it feels misleading.

We acknowledge the concern regarding the representation of adult male and female skeletons in Figure 4, panels A and B. To address this, we incorporated one scale bar and adjusted the images to better reflect the actual size contrast.

- Ossification Analysis section paragraph 2 change "frog's gender" to "frog's sex."
Yes, we changed this information in the manuscript, as suggested (p. 11).

- Figure 5 panel A, the label is overlapping "NF 59." For panels B and B' scale bars on these panels would help the reader understand the proportions. Yes, there is the 3mm scale bar from panel A and as stated in the caption, but including them in the B panels could help even if panel B had a scale bar labeled at 0.25 mm and panel B' was 3 mm.
We have implemented all the necessary adjustments in Fig. 5A and B, as per your request.

- Segmentation of Selected Internal Soft Organ section, perhaps more commentary on the ability to observe the development of the segmentation of the brain regions: cbh:

Powered by Editorial Manager® and ProduXion Manager® from Aries Systems Corporation

cerebral hemispheres; cbl: cerebellum; dch: diencephalon; mob: medulla oblongata; opl: optic lobes; sp: spinal cord while clearly shown in Figure 6, some accompanying description in the text would help readers in general or give the implication that microCT analysis of mutant or diseased frogs could help identify physical characteristics of frogs with developmental or neurological disorders. This would help transition from the analysis of a specific organ to the next section Further Biological Potential of Xenopus's Data.

We implemented these paragraphs into the main text:
Page 13: It is noteworthy that our micro-CT data allows for detailed observation of the developmental segmentation of various regions of the brain, which is very hard to dissect, especially from Xenopus adults (personal observation). Specifically, Fig. 6A-E, A'-E', and Suppl. Video 4 provide a clear morphological visualization of the individual brain areas such as cerebral hemispheres (cbh), cerebellum (cbl), diencephalon (dch), medulla oblongata (mob), optic lobes (opl), and spinal cord (sp), in the tadpole and adult frog brains, as well as to follow how these structures are developing in time course. For more information about the X. laevis' brain and its detailed description, including its regeneration in developing tadpoles, we refer readers to the recent publication (Ishii, Yoshida et al. 2023).
Page 15: Acknowledging the significance of our micro-CT data, it is also possible to address the common limitations linked to the absence of three-dimensional structural information about organs, such as the brain. The potential application of micro-CT, particularly in the study of mutants or diseased frogs, can offer valuable insights into the physical characteristics associated with developmental or neurological disorders. This not only holds true for Xenopus but also presents potential implications for humans. Notably, similar micro-CT-based studies involving, for instance, rat brains further underscore the relevance of our assumption (Kastner, Kharazia et al. 2020). Alternatively, the integration of micro-CT data with the Crispr/Cas9 system can be employed for disease modeling, as demonstrated in the recent study involving Xenopus tadpoles (Abu-Daya and Godwin 2023).

- These analyses, while thorough accompanied by novel visuals, require statistical implementation of multiple tadpoles and frogs per NF stage to account for variation in samples and to bolster the claims stated in skull thickness, the head mass and eye distance changes, increased length of the long bones during maturation, and femural ossification cartilage to bone ratios. This may constitute a suggested major revision to perform these analyses.

Certainly, we appreciate the reviewer's attention to the statistical robustness of our analysis. We want to assure you that we have now included a sufficient number of samples to support our findings. In Fig 2B', 4C, and 5C+D, we present the relevant data to underscore the trends observed in characteristics like skull thickness, increased length of long bones during maturation, and ossification cartilage to bone ratios. Moreover, to streamline the presentation, Graph 2D has been relocated to the Supplementary material. We trust that this addresses the concern raised by Reviewer #2.

Reviewer #3:
Laznovsky et al. present a nice compendium of micro-CT-based digital volumes of several stages of Xenopus development. Given the prominence of this important model animal in studies of developmental biology and physiology, this dataset is quite useful and will be of interest to the community. That said, the study has some key limitations that will limit its utility for the research community, though these do not reduce the dataset's impact in the education and popular science realms, which is also a stated goal for the paper. Overall, I recommend publication after an effort has been made to address the following concerns.
We also want to convey our gratitude to Reviewer #3 for the valuable time and effort in reviewing our manuscript.

1. The atlas adequately samples developmental stages from late tadpole through metamorphosis. However, as far as I can tell only a single sample has been imaged at

Powered by Editorial Manager® and ProduXion Manager® from Aries Systems Corporation

each stage. Thus, the quantifications of inter-stage differences shown here (Fig. 2, 4, 5) are at best very rough estimates and also provide no information about intra-stage variability in these metrics. This is not a fatal weakness, but it is an important caveat that I believe should be very explicitly stated in the text and in the figure legend of relevant figures.

We conducted all requisite experiments to reproduce the data from Fig. 2, 4, and 5, as per your request. Please refer to the respective figures for detailed information. We trust that this addresses the concern raised by Reviewer #3.

2. I am very disappointed that the rich history of microCT on Xenopus seems to have been entirely ignored by these authors. MicroCT has already been used to describe the skull, the brain, liver, blood vessels, etc. during Xenopus development. (Just a few papers the authors should read are: Slater et al., PLoS One 2009; Senevirathnea et al., PNAS, 2019; Ishii et al., Dev. Growth, Diff. 2023; Zhu et al., Front. Zool 2020.) It has also been used for comparative studies of other frogs (Kondo et al., Dev. Growth, Diff. 2022; Kraus, Anat. Rec. 2021; Jandausch et al., Zool. Anz. 2022; Paluh, et al., Evolution 2021, Paluh et al., eLife 2021). None of these -or the many other relevant papers- are discussed or cited here. The research community would be much better served if authors make a serious effort to integrate their methods and their results into this existing literature.

We regret any disappointment caused to the reviewer and would like to clarify the rationale behind this apparent misunderstanding. While we acknowledge the publications mentioned by the reviewer, our original intent in the manuscript was to emphasize that the micro-CT technique had not been applied at the whole-organism level in Xenopus, except for a single instance (Porro and Richards, J Anat, 2017). However, we now concur with the reviewer's perspective and have made a concerted effort to align our methods and results more closely with the existing literature (p. 4):
In response to these limitations, a detailed description of an adult male frog's anatomy (as a complete organism) using a non-destructive micro-CT method was recently provided (Porro and Richards 2017). Micro-CT, to be candid, has already been employed to some extent as a valuable tool in selected stages of X. laevis' embryos, tadpoles, and frogs to explore localized or specific occurrences, such as gastrulation (Moosmann, Ershov et al. 2014), craniofacial (Slater, Liu et al. 2009) and urostyle (Senevirathne, Baumgart et al. 2020) development, skeletal morphology (Vasquez, Hansen et al. 2008, Matthews and du Plessis 2016), limb (skeletal) regeneration (Feng, Milner et al. 2011, Chen, Lin et al. 2012, Golding, Guay et al. 2016), along with brain regeneration (Ishii, Yoshida et al. 2023), and the anatomy of cranial and anterior spinal nerves (Naumann and Olsson 2018), the head (Descamps, Sochacka et al. 2020, Murugan, Vigran et al. 2022), and several internal organs (Descamps, Sochacka et al. 2014). Besides X. laevis itself, micro-CT has been utilized in closely related frogs to investigate, for instance, chondrocranium organization in A. obstetricans (Krings, Muller et al. 2017), skeleton (Herrel and Bonneaud 2012) and digit (Zhang, Wilson et al. 2021) morphology in X. tropicalis, cortical bone morphology in various anuran amphibians (Kondo, Iwamoto et al. 2023), re-evolution of lost mandibular teeth in G. guentheri (Paluh, Dillard et al. 2021, Paluh, Riddell et al. 2021), together with cranial musculoskeletal structures in P. fuscus (Jandausch, Schwarz et al. 2022), lungs rearrangements during metamorphosis in M. fissipes (Chang, Zhang et al. 2022), gill formation during metamorphosis in B. bufo (Kraus and Metscher 2022), and metabolic reorganization in metamorphic R. omeimontis tadpoles (Zhu, Chang et al. 2020). Nevertheless, the spatiotemporal dynamics of X. laevis' late development on the level of a whole organism, along with the comparison between adult male and female frogs, remain largely unexplored in sufficient detail.

3. An opportunity may have been missed here to provide some truly new biological insights: The gut remodels substantially during metamorphosis, but to my knowledge that has NOT been previously examined by microCT. It may not work, as the gut may simply be too soft to visualize, but then again, it may be worth trying.

We appreciate the reviewer's suggestion and have exerted considerable effort to provide further insights into the development of the Xenopus gut using our samples (refer to p. 14 and Figure 7).

Powered by Editorial Manager® and ProduXion Manager® from Aries Systems Corporation

	EDITOR'S COMMENT:<br>In addition, please register any new software application in the bio.tools and SciCrunch.org databases to receive RRID (Research Resource Identification Initiative ID) and biotoolsID identifiers, and include these in your manuscript. Computational workflows should be registered in workflowhub.eu and the DOIs cited in the relevant places in the manuscript. These will facilitate tracking, reproducibility and re-use of your tool.<br><br>As for RRIDs, we implemented this sentence into the manuscript (p. 7):<br>The reconstructed data were imported into VG Studio MAX 2023.4 software (Volume Graphics GmbH, Heidelberg, Germany), in which the measured data were segmented, analyzed, and visualized using VG Studio MAX, RRID: SCR_017997. The segmentation of Xenopus guts was done manually by an operator using the software Avizo 2020.2 (Thermo Fisher Scientific, Waltham, MA, USA) (Avizo 3D Software, RRID: SCR_014431).
**Additional Information:**	
**Question**	**Response**
Are you submitting this manuscript to a special series or article collection?	No
**Experimental design and statistics**<br><br>Full details of the experimental design and statistical methods used should be given in the Methods section, as detailed in our Minimum Standards Reporting Checklist. Information essential to interpreting the data presented should be made available in the figure legends.<br><br>Have you included all the information requested in your manuscript?	Yes
**Resources**<br><br>A description of all resources used, including antibodies, cell lines, animals and software tools, with enough information to allow them to be uniquely identified, should be included in the Methods section. Authors are strongly encouraged to cite Research Resource Identifiers (RRIDs) for antibodies, model organisms and tools, where possible.	Yes

Powered by Editorial Manager® and ProduXion Manager® from Aries Systems Corporation

Have you included the information requested as detailed in our Minimum Standards Reporting Checklist?	
**Availability of data and materials**<br><br>All datasets and code on which the conclusions of the paper rely must be either included in your submission or deposited in publicly available repositories (where available and ethically appropriate), referencing such data using a unique identifier in the references and in the "Availability of Data and Materials" section of your manuscript.<br><br>Have you have met the above requirement as detailed in our Minimum Standards Reporting Checklist?	Yes

Powered by Editorial Manager® and ProduXion Manager® from Aries Systems Corporation

Manuscript

Click here to access/download;Manuscript;Manuscript_Xenopus_microCT_1

Unveiling Vertebrate Development Dynamics in Frog *Xenopus laevis* using Micro-CT Imaging

Laznovsky Jakub[1] (jakub.laznovsky@ceitec.vutbr.cz), Kavkova Michaela[1] (michaela.kavkova@med.muni.cz), Reis Alice Helena[2] (alicehreis@gmail.com), Robovska-Havelkova Pavla[3] (pavla.robovska@email.cz), Maia Lorena Agostini[4] (lorena.maia@sci.muni.cz), Krivanek Jan[5] (jan.krivanek@med.muni.cz), Zikmund Tomas[1] (tomas.zikmund@ceitec.vutbr.cz), Kaiser Jozef[1,6] (jozef.kaiser@ceitec.vutbr.cz), Buchtova Marcela[4,7] (buchtova@iach.cz), and Harnos Jakub[4*] (harnos@sci.muni.cz).

[1]Central European Institute of Technology, Brno University of Technology, 612 00 Brno, Czech Republic.

[2]Department of Chemical Engineering, Columbia University, New York, NY 10025, USA; and Department of Genetics and Development, Columbia Stem Cell Initiative, Columbia University Irving Medical Center, New York, NY 10032, USA.

[3]Department of Zoology, Faculty of Science, University of South Bohemia, 370 05 Ceske Budejovice, Czech Republic.

[4]Department of Experimental Biology, Faculty of Science, Masaryk University, 625 00 Brno, Czech Republic.

[5]Department of Histology and Embryology, Faculty of Medicine, Masaryk University, 625 00 Brno, Czech Republic.

[6]Institute of Physical Engineering, Faculty of Mechanical Engineering, Brno University of Technology, 616 69 Brno, Czech Republic.

[7]Laboratory of Molecular Morphogenesis, Institute of Animal Physiology and Genetics, v.v.i., Czech Academy of Sciences, 602 00 Brno, Czech Republic.

[*]Corresponding author (ORCID: 0000-0002-0752-9260): harnos@sci.muni.cz

Jakub Laznovsky [0000-0002-1323-5269]; Michaela Kavkova [0000-0001-7435-9292]; Alice Reis [0000-0002-7431-5502]; Pavla Robovska-Havelkova [0000-0002-5734-9890]; Lorena Agostini Maia [0009-0008-7730-9002]; Jan Krivanek [0000-0002-7590-187X]; Tomas Zikmund [0000-0003-2948-5198]; Jozef Kaiser [0000-0002-7397-125X]; Marcela Buchtova [0000-0002-0262-6774]; and Jakub Harnos [0000-0002-0752-9260].

Abstract

Background:

Xenopus laevis, the African clawed frog, is a versatile vertebrate model organism in various biological disciplines, prominently in developmental biology to study body plan reorganization during metamorphosis. However, a notable gap exists in the availability of comprehensive datasets encompassing *Xenopus'* late developmental stages.

Findings:

This study utilized micro-computed tomography (micro-CT), a non-invasive 3D imaging technique with micrometer-scale resolution, to explore the developmental dynamics and morphological changes in *Xenopus laevis*. Our approach involved generating high-resolution images and computed 3D models of developing *Xenopus* specimens, spanning from premetamorphosis tadpoles to fully mature adults. This dataset enhances our understanding of vertebrate development and supports various analyses. We conducted a careful examination, analyzing body size, shape, and morphological features, focusing on skeletogenesis, teeth, and organs like the brain and gut at different stages. Our analysis yielded valuable insights into 3D morphological changes during *Xenopus'* development, documenting details previously unrecorded. These datasets hold the solid potential for further morphological and morphometric analyses, including segmentation of hard and soft tissues.

Conclusions:

Our repository of micro-CT scans represents a significant resource that can enhance our understanding of *Xenopus'* development and the associated morphological changes in the future. The widespread utility of this amphibian species, coupled with the exceptional quality of our scans, which encompass a comprehensive series of developmental stages, opens up extensive opportunities for their broader research application. Moreover, these scans can be used in virtual reality, 3D printing, and educational contexts, further expanding their value and impact.

Graphical abstract & lay summary

3D computed images of selected developmental stages of *X. laevis*.

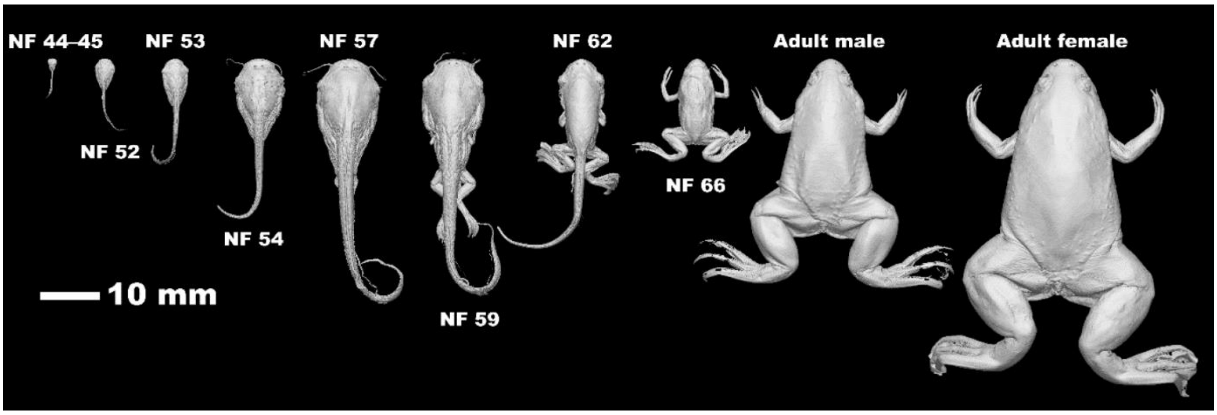

Lay summary: X-ray tomography was used to examine the African clawed frog (*Xenopus laevis*). This extensive data set of specimens from tadpoles to adult frogs opens avenues to novel insights into the changes and developmental dynamics of selected structures, leading eventually to an improved understanding of this crucial animal model.

Keywords

Xenopus laevis, development, vertebrates, micro-computed tomography, morphological changes.

Background

Xenopus laevis (NCBI:txid8355), commonly known as the African clawed frog, serves as a fundamental model organism in the field of life sciences. Its widespread adoption particularly in biological research can be attributed to the ease of breeding and housing, as well as the substantial size and manipulability of its eggs and embryos [1, 2]. Over the years, this species has been extensively investigated across various disciplines, including genetics, embryology, and developmental, cell, and regenerative biology [3-5]. While initial attempts at anatomical descriptions of *X. laevis* date back a century [6], subsequent studies have, to varying degrees, concentrated only on specific anatomical regions [7-25]. Despite these valuable contributions, many past approaches to describing frog anatomy failed to preserve the intricate morphological details, making them unsuitable for detailed and comparative studies. In response to these limitations, a detailed description of an adult male frog's anatomy as a complete organism using a non-destructive micro-CT method was recently provided [7]. Micro-CT, to be candid, has already been employed to some extent as a valuable tool in selected stages of *X. laevis'* embryos, tadpoles, and frogs to explore localized or specific occurrences, such as gastrulation [26], craniofacial [27] and urostyle [28] development, skeletal morphology [29, 30], limb (skeletal) regeneration [31-33], along with brain regeneration [34], and the anatomy of cranial and anterior spinal nerves [35], the head [36, 37], and several internal organs [38]. Besides *X. laevis* itself, micro-CT has also been utilized in closely related frogs to investigate, for instance, chondrocranium organization in *A. obstetricans* [39], skeleton [40] and digit [41] morphology in *X. tropicalis*, cortical bone morphology in various anuran amphibians [42], re-evolution of lost mandibular teeth in *G. guentheri* [43, 44], together with cranial musculoskeletal structures in *P. fuscus* [45], lungs rearrangements during metamorphosis in *M. fissipes* [46], gill formation during metamorphosis in *B. bufo* [47], and metabolic reorganization in metamorphic *R. omeimontis* tadpoles [48]. Nevertheless, the spatiotemporal dynamics of *X. laevis'* late development at the level of a whole organism, along with the comparison between adult male and female frogs, remain largely unexplored in sufficient detail.

To fill this knowledge gap and gain a more comprehensive understanding of *X. laevis'* late development at the level of a whole organism, we employed micro-CT, a powerful imaging technology

for examining small objects at the micrometer scale [49, 50]. Micro-CT utilizes X-rays to generate high-resolution 3D images, making it an ideal tool for investigating developmental and morphological changes, particularly in embryos, tadpoles, and small animals [49, 50]. In principle, micro-CT imaging is based on capturing a series of 2D X-ray radiographs from various angles and then mathematically processing them through tomographic reconstruction, resulting in a 3D matrix representing volume density. An advantage of micro-CT lies in its ability to image bones and, when combined with various contrast methods, also soft tissues and blood vessels within the same sample [49, 50].

Through micro-CT, we present high-resolution images and computed 3D models of developing *X. laevis* tadpoles, froglets, and adult frogs, which offer great potential for research fields like developmental and comparative biology. Additionally, we conducted preliminary analyses of this dataset to illustrate its versatility. Specifically, our preliminary examination covered morphological changes in body size, shape, and skeletogenesis from selected premetamorphosis, prometamorphosis, and climax metamorphosis stages through the froglet stage up to adulthood. Our findings unveil promising and opening insights into the intricate morphological dynamics of late *X. laevis's* development. To sum up, this research contributes a unique dataset concerning the developmental and morphological changes in *X. laevis*, including the adult stages, shedding light on the dynamics of vertebrate development, and bearing broader implications for future analyses of *Xenopus*.

Data Description/Sampling Strategy

In our micro-CT study of *Xenopus,* we employed a careful sampling strategy to encompass the entire developmental spectrum of this amphibian species. The selection of developmental stages was based on specific morphological features and markers that serve as critical indicators of *X. laevis'* development (see below). By strategically choosing nine distinct developmental stages, we aimed to capture the comprehensive process of morphogenesis, spanning from premetamorphosis tadpoles to fully mature adult frogs. These stages were carefully chosen with reference to specific criteria, such as the presence and dimensions of legs and the tail, or the rearrangements of the head, as outlined in the tables of Nieuwkoop and Faber [51], and Zahn and colleagues [52].

Our sampling included the following crucial stages of *X. laevis'* development:

Premetamorphosis (stages 44-45, 52, and 53, according to Nieuwkoop and Faber, NF): These early stages provide insight into the initial stages of development on metamorphosis, as *Xenopus* transforms from tadpole to froglets.

Prometamorphosis (NF stages 54 and 57): This phase represents an intermediate stage where significant changes in the head, limb buds, and tail are occurring, leading slowly towards the climax metamorphosis.

Climax Metamorphosis (NF stages 59 and 62): These stages are characterized by the most profound changes, marking the peak of metamorphosis.

Froglets (NF stage 66): Froglets represent a transitional stage, bridging the gap between tadpoles and fully mature adult frogs.

Adult Male and **Female Frogs**: These adult stages represent the endpoint of *Xenopus* development, allowing us to observe fully mature individuals and to compare differences between sexes. We chose organisms that were approximately one year old for this micro-CT study.

Our selection of these key developmental stages was performed with regard to their significance in the overall developmental process of *X. laevis*. By examining these specific stages, we aimed to gain a solid data set, enabling a comprehensive understanding of the morphological transformations and developmental milestones that occur during *X. laevis'* development, from its primary aquatic tadpole phase to its secondary aquatic adult frog stage [53]. This sampling strategy provided a detailed and holistic view of *X. laevis'* late development, enabling us to perform valuable analyses leading to preliminary insights and conclusions about this species' morphological changes throughout its life cycle.

Methods/Source of Samples

Xenopus embryos were generated and cultivated following standard protocols. Briefly, testes were surgically removed from anesthetized males (20% MS-222, Sigma-Aldrich, A5040) and transferred to cold 1x Marc's Modified Ringers (MMR; 100mM NaCl, 2mM KCl, 1mM $MgSO_4$, 2mM $CaCl_2$, 5mM HEPES, buffered to pH 7.4), supplemented with 50 µg/mL of gentamycin (Sigma-Aldrich,

G3632). To induce egg laying, fully mature *Xenopus* females were injected with 260 U of human chorionic gonadotropin (Merck, Ovitrelle 250G) into the dorsal lymph sac approximately 12-16 hours before use and were kept overnight at 18°C. For fertilization, eggs were extracted from induced females directly into a Petri dish and mixed with a piece of testes in 0.1x MMR at 18-21°C. The subsequent development of *Xenopus* tadpoles, froglets, and adult frogs adhered to the approved cultivation protocols (see above). The *Xenopus* froglets and adult frogs were reared in XenopLus (catalog number RE18001301, Tecniplast, Italy) that is an advanced, fully automated system tailored for housing amphibians, guaranteeing excellent animal care and housing conditions. At a designated time point, *Xenopus* specimens were anesthetized and fixed in a buffered 4% paraformaldehyde solution (1004965000, Merck) for 3 hours (tadpoles) or overnight (froglets and adult frogs), and staged according to the tables of Nieuwkoop and Faber [51], and Zahn and colleagues [52]. Photographs of selected *Xenopus* specimens used for micro-CT analysis are presented in **Suppl. Fig. 1**.

Methods/Micro-CT Scanning

Prior to scanning, samples were placed in either a 2 ml Eppendorf tube, a 15 ml/50 ml Falcon tube (depending on sample size), or in the case of the adult frogs, a 500 ml plastic container. To prevent the motion and drying of the sample during the micro-CT scan, all samples were mounted in a 1% agarose gel (Top-BIO, P045). Micro-CT measurements were conducted using the GE Phoenix v|tome|x L 240 laboratory system, which is equipped with a 180kV/15W nanofocus X-ray tube and a 4000 x 4000-pixel flat panel detector with a 100 μm pixel size. Scan conditions for each stage are summarized in **Suppl. Table 1**. It is important to note that the X-ray source target utilized in this study was tungsten. The subsequent tomographic reconstruction was performed using the GE Phoenix datos|x 2.0 software. The reconstructed data were imported into VG Studio MAX 2023.4 software (Volume Graphics GmbH, Heidelberg, Germany), in which the measured data were segmented, analyzed, and visualized using VG Studio MAX, RRID: SCR_017997. The segmentation of *Xenopus* guts was done manually by an operator using the software Avizo 2020.2 (Thermo Fisher Scientific, Waltham, MA, USA) (Avizo 3D Software, RRID: SCR_014431).

All *Xenopus* samples were initially scanned in their native state to visualize bone structures. Subsequently, specimens were stained with 1% iodine (Penta, 21210-11000) in a 90% methanol (Penta, 17570-30250) solution to enhance the contrast and visualize soft tissues. As for teeth (Fig. 3), the separated jaws were scanned and stained. The dehydration and staining times for each *Xenopus* developmental stage are detailed in **Suppl. Table 2**.

Data Quality Control and Limitations

All analyzed samples, comprising *X. laevis* tadpoles, froglets, and adult frogs, were uniformly preserved and handled before scanning. The primary variable affecting data quality was the varied voxel size of each dataset, arising from different sample sizes. The smallest sample, a tadpole stage (NF 44-45), featured a voxel size of 5 µm, while the largest sample, an adult female frog, was scanned with a voxel size of 40 µm (for a voxel size of all scanned NF stages, see the appropriate column in **Suppl. Table 1** or **Fig. 1**).

The voxel size difference was directly correlated with sample dimensions. The GE L240 X-ray source's cone beam geometry and the detector's field of view allowed smaller samples to be placed closer to the X-ray source, resulting in a smaller voxel size (higher resolution) due to cone beam magnification. Although samples exhibited variable voxel sizes, this variability does not necessarily limit data analysis. Larger samples, despite having larger voxel sizes, still enabled the recognition of structures analyzed, as these structures were proportionately larger in larger animals scanned with larger voxel sizes.

All *Xenopus* specimens underwent two sequential scans. Initially, native scans were performed, following the principle of micro-CT imaging of samples in their native form without staining, enabling the visualization of dense mineralized structures like bones and teeth. Subsequently, all scans were stained as described in **Suppl. Table 2**. To prevent movement and drying of samples during data acquisition, all samples were fixed in polypropylene conical tubes with 1% agarose gel. Throughout data acquisition, no observable sample shrinkage occurred. If any negligible shrinkage or drying did occur, we anticipate the volume reduction to be proportionally consistent across all *Xenopus* samples.

Image data inherently exhibits limitations in terms of useful resolution and contrast among components. These constraints are integral to the imaging process and are not indicative of weaknesses in our study, but rather intrinsic characteristics of the imaging modality employed. It is essential to acknowledge that, in any imaging technique, there are practical boundaries to the level of detail and contrast achievable.

Atlas of *X. laevis'* Late Development

In order to provide a more comprehensive understanding of *X. laevis'* development during metamorphosis and organogenesis, we first collected specimens representing the key frog stages, including premetamorphosis, prometamorphosis, climax metamorphosis, as well as froglets, and adult male and female frogs (see Sampling Strategy). Initially, we performed native sample scanning for hard tissue visualization and subsequently stained and scanned the samples for soft tissue visualization (see Micro-CT Scanning). As a result, we were able to collect and analyze all major stages of *X. laevis'* late development. The entire collection of the *Xenopus* atlas is presented in **Fig. 1**, and all raw data can be found in the GigaDB repository (see the Data availability section)[54]. Subsequently, with this atlas, we provide here several examples of specific analyses of *X. laevis'* late development, including an analysis of head development, teeth, long bone growth dynamics, ossification, brain development with its associated nerves, and gut morphology.

Head Development Analysis

First, we delved into the intriguing process of head development and skull metamorphosis in climax metamorphosis tadpoles, froglets, and adult frogs (refer to **Fig. 2A**; **Suppl. Video 1**). Our initial investigation employed morphometric analysis to shed light on the developmental changes in the skull. All skulls were oriented in the same direction (the anterior skull part heading to the top of a page; A: anterior; P: posterior; R: right; and L: left) in a top-bottom view. Such images can be further used, for example, to evaluate how the individual calvarian bones are being rearranged during metamorphosis or, for example, to shed light on facial region development.

Next, we performed the examination of skull thickness (**Fig. 2B-B'**; **Suppl. Video 2**). This analysis revealed a non-linear relationship, with adult frogs possessing significantly thicker skulls compared to their younger counterparts. This investigation also suggests a positive correlation between skull mass and skull thickness.

Moving on, we made an introductory analysis regarding the eye distance during development, as illustrated in **Fig. 2C**. Our morphometric analysis (for an example of the measurement, see **Suppl. Fig. 2A**) revealed a progressive decrease in eye distance during developmental stages (roughly estimated in **Suppl. Fig. 2B-B'**). These changes actually occurred in *Xenopus* in two phases: a gradual increase in the pre-metamorphic stages (data not shown), followed by a peak at the onset of climax metamorphosis (NF stages 59 and 62), and finally, a decrease to a compact head configuration in froglets (NF stage 66) and adult frogs (**Fig. 2C**; and very roughly estimated in **Suppl. Fig. 2B-B'**). This adaptation aligns well with the frog's life strategy, transitioning from a water-dwelling tadpole with lateral eyes to an adult with eyes positioned on top of the head for a submerged lifestyle [55], reminiscent of crocodilians [56].

Based on the previous paragraphs, one can also explore the conservation analysis of the eye-head ratio with respect to frog sex. Findings of ours (and others [57]) indicate that *Xenopus* female heads exhibit an increase in volume compared to males **(Fig. 3A,B)**. However, though our effort was in no way directive, the eye distance in females does not remain at 100% of the head volume in males, but it is relatively less (for a preliminary estimate, see **Suppl. Table 3**). This observation, which can be even assessed also from the macroscopic scale, may be linked to visual perception requirements, as the proximity of the eyes is still crucial for certain aspects of vision [58]. However, our intention here was just to demonstrate that similar and precise analyses are feasible with micro-CT data. In summary, our micro-CT dataset can be further employed for the study of the head and its associated organs such as the eyes in *X. laevis*. For more information about the *X. laevis'* head morphology in general, we also refer to the handmade drawings by Zahn and colleagues [52].

Tooth Analysis

To enhance the depth of our analysis and increase its practical applicability, we conducted a comprehensive examination of the frog teeth, with an individual data set of an adult female jaw. Upon initial observation, the presence of teeth in *X. laevis* may not be readily apparent. This is due to the fact that teeth were exclusively found in the maxilla, referred to as maxillary teeth (**Fig. 4A-B**) and behind the maxillary arch on the vomeral bone – so-called vomeral teeth (not shown). Additionally, only a small portion of the maxillary tooth protruded into the oral cavity [59] (**Fig. 4C**). In contrast, the mandible contains no teeth (**Fig. 4D**). The shape of maxillary teeth was generally uniform (i.e., homodont dentition), typically resembling simple conical structures (see Fig. 4B). *Xenopus'* teeth exhibit an acrodont type of attachment, where they form an ankylotic attachment with the adjacent bone and are characteristic by regular renewal, a condition known as polyphyodont dentition. Notably, the phenomenon of tooth renewal in *Xenopus* is akin to what has been previously documented in other species, such as geckos [60, 61]. In agreement with this, we also noticed the evidence of the replacement in the maxillary teeth (**Fig. 4E**). These analyses revealed the progression of ankylosis, from the early development of teeth located closer to the gingiva to their full fusion with the adjacent bone (see Fig. 4E). Our *Xenopus* micro-CT data set thus unveils the previously concealed dental structures of *X. laevis*, provides a high-resolution revelation of their "hidden" teeth patterning, and offers new perspectives for studying teeth and their growth in *Xenopus*.

Micro-CT Data Analysis of Long Bone Growth Dynamics

Next, we assessed the growth dynamics of several long bones (shown in adult frogs in **Fig. 5A, B**, and **Suppl. Video 3**; refer to **Suppl. Fig. 3** to see how the analysis was performed). Based on our micro-CT analysis, in terms of sex, we found that adult females can be viewed as essentially enlarged males, indicating a proportional growth pattern (**Fig. 5C**). We also investigated whether the development of the left and right sides of *X. laevis* is uniform or divergent (**Suppl. Fig. 4**), and consistently, we observed L-R length symmetry in the length of all long bones. Therefore, we next focused our detailed examination only on one side of the frog, specifically the left side (L).

Our findings revealed that certain limb bones, particularly those in the hind legs, such as the femur and tibiofibular, exhibited relatively rapid growth, while other bones such as fore limb ones grew at a slower rate (**Fig. 5C**). Importantly, this difference was not attributed to absolute bone measurements but rather in relation to the animal's overall length (data not shown). This phenomenon may be linked to the frogs' utilization of their hind limbs for swimming, whereas even their forelimbs are primarily employed for food handling [62, 63]. Moreover, the length of the tarsal bone such as astragalus and calcaneum appears to be less critical for escape or startle responses, such as swim kicking, compared to the lengths of the femur and tibiofibular, which are correlated with the muscle mass of the thigh and the calf [7]. It is also intriguing to note that most bones initiated their growth from roughly the same size but terminated it at different dimensions. For example, metatarsals develop at a faster rate than metacarpals (**Fig. 5C**), which aligns with functional considerations, as outlined elsewhere [37]. Together, based on our micro-CT data, one can make new predictions for further experimental testing of (long) bone growth.

Ossification Analysis

Besides bone length, it is also feasible to analyze the bone mass using our *Xenopus* data set. Within the frog skeleton, two distinct types of bones can be generally distinguished in terms of their developmental origin. Dermal ossification, originating in the dermis, is evident in certain skull elements and in two bones of the pectoral girdle, namely the cleithrum and clavicle. Conversely, the remaining components of the postcranial skeleton consist of either cartilage or bones replacing the cartilage, achieved through endochondral ossification [64].

As the bones of the limbs undergo development in both tadpoles and adults, they typically comprise an ossified section alongside cartilage. Notably, the ossification process always commences at the central regions of long bones, as shown on the example of the femur (**Fig. 6A**). By assessment of the mass ratio between cartilage and bone (**Fig. 6B-B'**; **Suppl. Fig. 5**), we observed that, with a few exceptions, all long bones share relatively similar proportions (data not shown). Furthermore, in both adult males and females, there is a lack of dynamic growth akin to the developmental stages, with only

a noticeable increase in mass (**Fig. 6C**). After climax metamorphosis, no proliferative ossification occurs, and the process is limited to the calcification of cartilage, irrespective of the frog's sex (**Fig. 6C**). Moreover, the relative ratio of cartilage to an ossified bone diminishes as development progresses, exemplified by the femur, humerus, and radioulna (**Fig. 6C** and **6D**). Thus, researchers can further take advantage of these indications to compare the growth of different bones in various aged animals.

Segmentation of Selected Internal Soft Organs

Our dataset offers significant potential not only for the evaluation of hard bones but also for soft tissue such as internal organs. Precise segmentation of structures is essential because once the structure in focus is clearly distinguished, further assessment of its morphology and intraspecies differences becomes considerably easier and more accurate. This tool also provides the flexibility to describe each internal structure in detail either separately or in the context of its individual surrounding elements. In addition to studying differences during *X. laevis'* development, segmented structures can be utilized e.g., for investigating intersex differences. For an illustration of the possible application of our micro-CT data, here we further selected two key vertebrate organs, such as the brain and gut.

The brain. It is noteworthy that our micro-CT data allows for detailed observation of the developmental segmentation of various regions of the brain, which is very hard to dissect, especially from *Xenopus* adults (personal observation). Specifically, **Fig. 7A-E**, **A'-E'**, and **Suppl. Video 4** provide a clear morphological visualization of the individual brain areas such as cerebral hemispheres (cbh), cerebellum (cbl), diencephalon (dch), medulla oblongata (mob), optic lobes (opl), and spinal cord (sp), in the tadpole and adult frog brains, as well as to follow how these structures are developing in time course. For more information about the *X. laevis'* brain and its detailed description, including its regeneration in developing tadpoles, we refer readers to the recent publication [34]. Subsequently, we also asked whether we could analyze not only the brain but also the brain-associated nerves like *nervus opticus* using the micro-CT data set (**Fig. 7F**). Indeed, we could see that the nerves attached to eyes slowly elongated and then modified, as a specimen size changed during development (**Fig. 7F**).

Together, the quality of our micro-CT allows us to investigate not only the individual brain parts but also its associated structures.

The gut. During the metamorphosis of *X. laevis*, the gut undergoes significant remodeling, with the intestine shortening by approximately 75% over an 8-day period. The coiling pattern changes, with the outer loops initially coiling counterclockwise and the inner loops coiling clockwise, reversing at the ileum's switchback point [65, 66]. This remodeling is characterized by a stage-dependent sequential organization of nascent smooth muscle cells, which plays a crucial role in gut coiling morphogenesis [67].

To our best knowledge, this process has not been studied using micro-CT so far. Thus, we selected the gut as a second internal organ to dissect by the micro-CT technique, with a focus on the whole *X. laevis* atlas (**Fig. 8**). It is evident from the micro-CT images that it successfully depicted the gut structure with high quality and detail (**Fig. 8** and **Suppl. Video 5**). For more information about the *X. laevis* gut and its anatomy in general, we refer a reader to the *Xenopus* illustrations by Zahn and colleagues [52] and supporting literature [65, 66].

In sum, researchers will be able to use our micro-CT dataset to segment and explore several internal organs, including the brain or gut, as well as associated structures such as nerves, for further analytical and practical purposes, as mentioned in *Further Biological Potential* below.

Discussion

This study used micro-computed tomography (micro-CT) to create high-resolution 3D models of *Xenopus laevis (X. laevis)* from tadpoles to adults, revealing detailed developmental and morphological changes. The micro-CT scan repository enhances understanding of *X. laevis'* development and has applications in research, virtual reality, 3D printing, and education.

The *X. laevis'* development dataset not only offers valuable insights into this amphibian species but also presents a wealth of biological potential as it serves as a starting point for comparative, regenerative, and evolutionary biology as well as morphological study. The dataset's diverse specimen's representation and the high quality of the scans open up a realm of possibilities for advanced subsequent

analyses. One intriguing avenue of study lies in the examination of hard tissue growth. The dataset provides an ideal platform to investigate especially the long bones of *Xenopus* but also vertebrae, or a skull. Furthermore, the dataset facilitates the analysis of teeth or cartilage, which we have addressed using dedicated tools in VG Studio Max.

Next, this dataset lends itself to in-depth exploration by researchers interested in the anatomy of *Xenopus* including internal organs. The dataset's segmentation capabilities enable the precise segmentation of individual structures, which can then be further processed to obtain detailed information regarding their shape, volume, and relationships with surrounding structures in three dimensions (3D).

To enrich our understanding of adult *Xenopus* anatomy, morphology, and development, the dataset also includes scans of multiple *Xenopus* specimens. By combining these various analyses, the dataset offers comprehensive and detailed information on both hard and soft tissue morphology, making it a valuable resource for researchers studying this amphibian in the context of development.

Acknowledging the significance of our micro-CT data, it is also necessary to address the common limitations linked to the absence of 3D structural information about organs, such as the brain. The potential application of micro-CT, particularly in the study of mutants or diseased frogs, can offer valuable insights into the physical characteristics associated with developmental or neurological disorders. This is valid not only for *Xenopus* but also represents potential implications for humans. Notably, similar micro-CT-based studies involving, for instance, rat brains further underscore the relevance of our assumption [68]. Alternatively, the integration of the micro-CT technique with the Crispr/Cas9 approach can be even employed for disease modeling, as demonstrated in the recent study involving *Xenopus* tadpoles [69].

In conclusion, this extensive dataset serves as a cornerstone for advancing our knowledge of amphibian morphological structures and evolutionary adaptations.

Potential implications

Our *Xenopus* micro-CT dataset can be processed to create 3D representations such as views and videos, showcasing various developmental stages, including frog adulthood. This presents significant

educational and popularization potential, offering an innovative approach to understanding the complex the intricate process of *Xenopus* development.

In addition to providing 3D views and videos, the micro-CT dataset opens up exciting possibilities for 3D printing applications. Through micro-CT utilization, one can generate precise digital models of *Xenopus* tadpoles, froglets, adult frogs, and/or selected structures (see **Suppl. Fig. 6** for an example of 3D print). These 3D-printed replicas offer interactive learning experiences, enabling both students and the general public to physically engage with the developmental stages of *X. laevis*. By handling a 3D-printed animal sample or examining the detailed structures of its internal organs, learners of all ages can deepen their understanding in a tangible and captivating manner.

Moreover, these 3D models can serve as valuable resources for engaging the general public, particularly in museum settings, without requiring original specimens or samples (or their organs) preserved in fixatives. This immersive approach not only enhances understanding of *Xenopus's* development but also makes it accessible to a wider audience, fostering an appreciation for the intricacies of this fascinating organism.

Data Availability

All volumetric data of the scanned *Xenopus* specimens can be accessed in the *GigaScience* repository GigaDB [54]. These datasets are presented as already-reconstructed 8-bit TIFF stacks. To enhance accessibility, the data was converted from its original 16-bit format to 8 bits, making it more compact for downloading and easier to open and analyze. This reduction in data size is particularly beneficial given the computational demands associated with working with large data sets.

The provided videos showcase individual animals and highlight analyses conducted using the scanned data, as detailed in the supplementary files. Additionally, .STL files accompany the image stacks, enabling users to visually inspect the scanned animals in three dimensions. These .STL models are also accessible via the GigaDB repository [54].

While the deposited .TIFF format data can be viewed in any basic image viewer, for a comprehensive exploration of the 3D nature of the images and the analyses performed, it is

recommended to use a dedicated 3D data viewer. VG Studio MAX, a commercial software package from Volume Graphics GmbH, Germany, offers a wide range of tools for visualizing, manipulating, and analyzing 3D micro-CT image data. Its freeware version, MyVGL, is a suitable alternative for visualizing all datasets and their corresponding analyses. Another free software option is the Fiji ImageJ distribution from the National Institutes of Health, USA, or Avizo software (ThermoFisher, USA), both capable of opening individual image stacks and creating 3D renders of all scanned *Xenopus* specimens.

In summary, the volumetric data of scanned animals, available on the GigaDB repository [54], provides efficient accessibility through 8-bit TIFF stacks, complemented by .STL models, with recommended 3D exploration using software like VG Studio MAX, MyVGL, Avizo, or Fiji ImageJ. Printable 3D models are additionally available from SketchFab and Thingiverse. The SketchFab collections are as follows:

Xenopus development: https://sketchfab.com/GigaDB/collections/xenopus-laevis-development-3922d82fde27407ea1e7cc4622376178

Xenopus adult male - https://sketchfab.com/GigaDB/collections/xenopus-laevis-male-7eb069e65d2f49749ce4b4144dd5fc81

Xenopus adult female - https://sketchfab.com/GigaDB/collections/xenopus-laevis-female-b33299ca19604663a0cbdeb915f683e9

The Thingiverse links for the collection of printable models is as follows: https://www.thingiverse.com/thing:6620040

Additional Files

Supplementary Video 1: The skull of an adult *Xenopus* female in 3D view.

Supplementary Video 2: The skull of an adult *Xenopus* female in 3D view while using wall thickness analyses.

Supplementary Video 3: The skeleton of an adult *Xenopus* female in 3D view.

Supplementary Video 4: The brain of an adult *Xenopus* female in 3D view.

Supplementary Video 5: The gut of an adult *Xenopus* female in 3D view.

List of abbreviations

A: anterior; AS: astragalus; CA: calcaneum; cbh: cerebral hemispheres; cbl: cerebellum; dch: diencephalon; F: femur; H: humerus; L: left; MC: metacarpals; micro-CT: micro-computed tomography; MMR: Marc's Modified Ringers; MT: metatarsals; mob: medulla oblongata; NF stage: Nieuwkoop and Faber stage; opl: optic lobes; P: posterior; R: right; RU: radioulna; sp: spinal cord; SS: suprascapular; TF: tibiofibular.

Declarations

Our work with *X. laevis* adhered to Czech animal use and care laws and received approval from local authorities and committees (MSMT-30784/2022-1; Animal Care and Housing Approval: 45055/2020-MZE-18134, Ministry of Agriculture of the Czech Republic; and Animal Experiments Approval: CZ 62760214, State Veterinary Administration/Section for South Moravian Region).

Following the ARRIVE guidelines, frogs *X. laevis* were utilized in this study as a vertebrate model organism in accordance with ethical standards. The experimental procedures involving frogs were conducted with careful consideration of minimizing suffering and distress, adhering to approved protocols (see above). Furthermore, efforts were made to ensure transparency and reproducibility by clearly documenting the housing conditions, experimental procedures, and welfare measures implemented for the frogs throughout the study.

Consent for publication

Not applicable.

Competing interests

The authors declare no competing financial interests.

Funding

We are grateful for the funding provided by the CzechNanoLab Research Infrastructure supported by MEYS CR (grant no. LM2023051), the Faculty of Mechanical Engineering, Brno University of Technology, under grant no. FSI-S-23-8389 (awarded to J.K.), and the Czech Science Foundation, grants no. GA22-02794S (awarded to J.Kr. and M.B.) and GA22-06405S (awarded to J.H.). Additionally, we appreciate the support from the Grant Agency of Masaryk University, project no. MUNI/J/0004/2021 (awarded to J.H.). Together, these contributions enabled us to conduct this research. The funders had no role in study design, data collection and analysis, decision to publish, or preparation of the manuscript.

Authors' contributions

J.H. conceived the research idea, manipulated with *Xenopus*, wrote the original draft, created a preliminary figure layout, and supervised the overall work.

J.L. contributed to the original draft writing, data visualization, and figure creation.

J.L. and M.K. conducted micro-CT experiments and analyzed the data.

A.R., P.R.H., L.A.M., J.Kr., M.B., and J.H. provided expertise in the biological interpretation of data.

J.Kr., T.Z., J.Ka., M.B., and J.H. secured funding for the project.

M.K., M.B., and J.H. developed the initial concept of the *Xenopus* atlas.

All authors edited and approved the original manuscript and the revised version.

Acknowledgements

We thank Lenka Doubkova and Eva Slabakova for their administrative support and assistance.

Figure legend

Figure 1. The atlas of *Xenopus laevis's* late development.
A) 3D renders of soft tissues of selected *Xenopus* developmental NF stages.
A') Cross-sections of soft tissues. The section level was selected based on capturing the brain area of *Xenopus* specimens.
A'') 3D renders of the skeleton. In the first three conditions (NF 44-53), the bones are zoomed in and shown in the red rectangle on the left.
The relevant scale bar and voxel size are depicted on bottom or top of each picture.
All views are from dorsal view with the cranial side pointing to the right.
A: anterior; P: posterior; R: right; L: left.

Figure 2. The analyses of the head development. A) The series of *Xenopus* skulls of selected stages. The scale bar with values in mm is shown on the left bottom. **B)** The skull thickness of selected stages is displayed. The scale bar with values in mm is shown on the left side. B') The relative distribution of bone thickness for each skull is shown. The experiment involved conducting three repetitions for each developmental stage, with the results depicted as means accompanied by standard deviations (± SD). **C)** The overview of developing heads with highlighted eyes is shown.

Figure 3. Demonstrative interactive SketchFab visualisations of 3D reconstructions highlighting that *Xenopus* female heads exhibit an increase in volume compared to males. a) Skull of *Xenopus laevis* adult female (Link: https://sketchfab.com/3d-models/skull-of-xenopus-laevis-female-321240df5a5741d39937f65d457c1594). b) Skull of *Xenopus laevis* adult male (Link: https://sketchfab.com/3d-models/skull-of-xenopus-laevis-male-192dbbceb71f4b73a1097a7cdf67e5ae).

Figure 4 The analysis of teeth in an adult frog. A) The frontal view of an adult female frog skull is depicted with an upper maxillary arch with maxillary teeth visualized in yellow. **B)** The lateral and top view on the right half of the maxillary arch with visualized teeth from an adult female frog. Three different developmental stages of teeth are highlighted by different colors (yellow, cyan, orange). **C)** The stained micro-CT scan shows that only a small portion of the tooth extends into the oral cavity. The arrows point to teeth rows which do not penetrate the oral cavity. **D)** The lateral view of the mandible of an adult female frog confirms the absence of teeth in this area. **E)** The lateral view of the rostral part of the maxilla displays different stages of teeth during the replacement of tooth rows in detail.

Figure 5. The analysis of frog skeletogenesis. A) The adult male frog with analyzed bones is depicted. **B)** The adult female frog with analyzed bones is displayed. **C)** The graph demonstrating the analyzed bones throughout the *Xenopus laevis's* late development. The experiment involved conducting three repetitions for each long bone measurement, with the results depicted as means accompanied by standard deviations (± SD). However, error bars are depicted only for cases demonstrating statistically significant differences, while they are not displayed for insignificant variations. Statistical analysis was performed using a two-way analysis of variance (ANOVA) followed by Tukey post hoc test for multiple comparisons; ****, p <0,0001.A: anterior; AS: astragalus; CA: calcaneum; F: femur; H: humerus; L: left; MC: metacarpals; MT: metatarsals; P: posterior; R: right; RU: radioulna; TF: tibiofibular.

Figure 6. The analysis of long bones including their cartilage and ossification. A) The collection of femur bone throughout the late development. **B-B')** The femur from different developmental stages such as NF 59 (B) and an adult female (B') with a focus on cartilage (in red) and bone (yellow). The view of bones is maximized and not in real proportions. For size proportions, see Fig. 5A. The absolute (in **C**) and relative (in **D**) quantification of a bone and cartilage mass for selected long bones such as the femur, humerus, and radioulna for each developmental stage, with the results depicted as means accompanied by standard deviations (± SD). Statistical analysis was performed using a two-way analysis of variance (ANOVA) followed by Tukey post hoc test for multiple comparisons; **, p <0,01; ns, p>0,05. Additionally, it should be noted that in A), the cartilage is not visible, while in B), the stained data allows for a clear separation of cartilage from bones. The merged data in Fig. 5B) emphasizes the isolated cartilage.

Figure 7. The analysis of brain development. A-E) Individual stages of *Xenopus* brain and their details in (A'-E'), the rostrum pointing to the right, and **F)** nervus opticus attached to eyes (in cyan), the rostrum is pointing up. cbh: cerebral hemispheres; cbl: cerebellum; dch: diencephalon; mob: medulla oblongata; opl: optic lobes; sp: spinal cord.

Figure 8. The analysis of gut development. A-A') Ventral and dorsal views of individual stages of *Xenopus* gut and their details for each developmental stage (**B-I**, **B'-I'**), except guts from frog adults.

References

1. Wheeler, GN, Brandli, AW. Simple vertebrate models for chemical genetics and drug discovery screens: lessons from zebrafish and *Xenopus*. *Dev Dyn*. 2009;238, 1287-1308, doi:10.1002/dvdy.21967.
2. Matsuda, M, Sokol, SY. *Xenopus* neural tube closure: A vertebrate model linking planar cell polarity to actomyosin contractions. *Curr Top Dev Biol*. 2021;145, 41-60, doi:10.1016/bs.ctdb.2021.04.001.
3. Gurdon, JB, Hopwood, N. The introduction of *Xenopus laevis* into developmental biology: of empire, pregnancy testing and ribosomal genes. *Int J Dev Biol*. 2000;44, 43-50.
4. Harland, RM, Grainger, RM. *Xenopus* research: metamorphosed by genetics and genomics. *Trends Genet*. 2011;27, 507-515, doi:10.1016/j.tig.2011.08.003.
5. Phipps, LS, Marshall, L, Dorey, K, Amaya, E. Model systems for regeneration: *Xenopus*. *Development*. 2020;147, doi:10.1242/dev.180844.
6. Grobbelaar, CS. Beiträge zu einer anatomischen Monographie von *Xenopus laevis* (Daud.). *Zeitschrift für Anatomie und Entwicklungsgeschichte*. 1924;72, 131-168, doi:10.1007/BF02117712.
7. Porro, LB, Richards, CT. Digital dissection of the model organism *Xenopus laevis* using contrast-enhanced computed tomography. *J Anat*. 2017;231, 169-191, doi:10.1111/joa.12625.
8. Exner, CRT, Willsey, HR. *Xenopus* leads the way: Frogs as a pioneering model to understand the human brain. *Genesis*. 2021;59, e23405, doi:10.1002/dvg.23405.
9. Rockova, H, Rocek, Z. Development of the pelvis and posterior part of the vertebral column in the Anura. *J Anat*. 2005;206, 17-35, doi:10.1111/j.0021-8782.2005.00366.x.
10. Green, T. On the Pelvis of the Anura: a Study in Adaptation and Recapitulation. *Proc Zool Soc Lond.* 1931;1259-1290 (Wiley Online Library).
11. Dunlap, DG. The comparative myology of the pelvic appendage in the Salientia. *J Morphol*. 1960;106, 1-76,doi:10.1002/jmor.1051060102.
12. Palmer, M. Expanded ilio-sacral joint in the toad *Xenopus laevis*. *Nature*. 1960;187, 797-798. doi:10.1038/187797a0.
13. Emerson, SB. Frog postcranial morphology: identification of a functional complex. *Copeia*. 1982, 603-613,doi:10.2307/1444660.
14. Van Dijk, DE. Longitudinal sliding articulations in pipid frogs: research in action. *S Afr J Sci*. 2002;98, 555-556,doi:10.10520/EJC97426.
15. Prikryl, T, Aerts, P, Havelkova, P, Herrel, A, Rocek, Z. Pelvic and thigh musculature in frogs (Anura) and origin of anuran jumping locomotion. *J Anat*. 2009;214, 100-139, doi:10.1111/j.1469-7580.2008.01006.x.
16. Robovska-Havelkova, P. How can ontogeny help us to understand the morphology of anuran pectoral girdle? *Zoomorphology*. 2010;129, 121-132, doi:10.1007/s00435-010-0105-9.
17. Ryke, PAJ. The ontogenetic development of the somatic musculature of the trunk of the aglossal anuran *Xenopus laevis* (Daudin). 1953;34, 1-70, doi:10.1111/j.1463-6395.1953.tb00367.x.
18. Paterson, NF. The Head of *Xenopus laevis*. *J Cell Sci*. 1939;2, 161-232, doi:10.1242/jcs.s2-81.322.161.
19. Trueb, L, Hanken, J. Skeletal development in *Xenopus laevis* (Anura: pipidae). *J Morphol*. 1992;214, 1-41, doi:10.1002/jmor.1052140102.
20. Rocek, Z. Origin and evolution of the anuran postnasal wall and adjacent parts of the palatoquadrate. *Ethol Ecol Evol*. 1991;5, 247-265, doi:10.1080/08927014.1993.9523108.
21. Smirnov, SV. Postmaturation skull development in *Xenopus laevis* (Anura, Pipidae): late-appearing bones and their bearing on the pipid ancestral morphology. *Russ J Herpetol*. 1994;1, 21-29.
22. Haas, A. Mandibular arch musculature of anuran tadpoles, with comments on homologies of amphibian jaw muscles. *J Morphol*. 2001;247, 1-33, doi:10.1002/1097-4687(200101)247:1<1::AID-JMOR1000>3.0.CO;2-3.

23. Ziermann, JM, Olsson, L. Patterns of spatial and temporal cranial muscle development in the African clawed frog, *Xenopus laevis* (Anura: Pipidae). *J Morphol*. 2007;268, 791-804, doi:10.1002/jmor.10552.
24. Gross, JB, Hanken, J. Segmentation of the vertebrate skull: neural-crest derivation of adult cartilages in the clawed frog, *Xenopus laevis*. *Integr Comp Biol*. 2008;48, 681-696, doi:10.1093/icb/icn077.
25. Ziermann, JM, Diogo, R. Cranial muscle development in frogs with different developmental modes: direct development versus biphasic development. *J Morphol*. 2014;275, 398-413, doi:10.1002/jmor.20223.
26. Moosmann, J, Ershov, A, Weinhardt, V et al. Time-lapse X-ray phase-contrast microtomography for in vivo imaging and analysis of morphogenesis. *Nat Protoc*. 2014;9, 294-304, doi:10.1038/nprot.2014.033
27. Slater, BJ, Liu, KJ, Kwan, MD, Quarto, N, Longaker, MT. Cranial osteogenesis and suture morphology in *Xenopus laevis*: a unique model system for studying craniofacial development. *Plos One*. 2009;4, e3914, doi:10.1371/journal.pone.0003914.
28. Senevirathne, G, Baumgart, S, Shubin, N, Hanken, J, Shubin, NH. Ontogeny of the anuran urostyle and the developmental context of evolutionary novelty. *Proc Natl Acad Sci U S A*. 2020;117, 3034-3044, doi:10.1073/pnas.1917506117.
29. Matthews, T, Du Plessis, A. Using X-ray computed tomography analysis tools to compare the skeletal element morphology of fossil and modern frog (Anura) species. *Palaeontol Electron*. 2016;19, 1-46, doi:10.26879/557.
30. Vasquez, SX, Hansen, MS, Bahadur, AN et al. Optimization of volumetric computed tomography for skeletal analysis of model genetic organisms. *Anat Rec (Hoboken)*. 2008;291, 475-487, doi:10.1002/ar.20670.
31. Chen, Y, Lin, G, Chen, Y, Fok, A, Slack, JM. Micro-computed tomography for visualizing limb skeletal regeneration in young *Xenopus* frogs. *Anat Rec (Hoboken)*. 2012;295, 1562-1565, doi:10.1002/ar.22496.
32. Golding, A, Guay, JA, Herrera-Rincon, C, Levin, M, Kaplan, DL. A Tunable Silk Hydrogel Device for Studying Limb Regeneration in Adult *Xenopus Laevis*. *Plos One*. 2016;11, e0155618, doi:10.1371/journal.pone.0155618.
33. Feng, L, Milner, DJ, Xia, C et al. *Xenopus laevis* as a novel model to study long bone critical-size defect repair by growth factor-mediated regeneration. *Tissue Eng Part A*. 2011;17, 691-701, doi:10.1089/ten.TEA.2010.0123.
34. Ishii, R, Yoshida, M, Suzuki, N, Ogino, H, Suzuki, M. X-ray micro-computed tomography of *Xenopus* tadpole reveals changes in brain ventricular morphology during telencephalon regeneration. *Dev Growth Differ*. 2023;65, 300-310, doi:10.1111/dgd.12881.
35. Naumann, B, Olsson, L. Three-dimensional reconstruction of the cranial and anterior spinal nerves in early tadpoles of *Xenopus laevis* (Pipidae, Anura). *J Comp Neurol*. 2018;526, 836-857, doi:10.1002/cne.24370.
36. Descamps, E, Sochacka, A, Kegel, BD et al. Soft tissue discrimination with contrast agents using micro-CT scanning. 2020;144, 20-40, doi: 10.26496/bjz.2014.63.
37. Murugan, NJ, Vigran, HJ, Miller, KA et al. Acute multidrug delivery via a wearable bioreactor facilitates long-term limb regeneration and functional recovery in adult *Xenopus laevis*. *Sci Adv*. 2022;8, eabj2164, doi:10.1126/sciadv.abj2164.
38. Descamps, E, Sochacka, A, De Kegel, B et al. Soft tissue discrimination with contrast agents using micro-CT scanning. *Belg J Zool*. 2014;144.
39. Krings, M, Muller, H, Heneka, MJ, Rodder, D. Modern morphological methods for tadpole studies. A comparison of micro-CT, and clearing and staining protocols modified for frog larvae. *Biotech Histochem*. 2017;92, 595-605, doi:10.1080/10520295.2017.1369162.
40. Herrel, A, Bonneaud, C. Temperature dependence of locomotor performance in the tropical clawed frog, *Xenopus tropicalis*. *J Exp Biol*. 2012;215, 2465-2470, doi:10.1242/jeb.069765.

41. Zhang, M, Wilson, SS, Casey, KM et al. Degenerative Osteoarthropathy in Laboratory Housed *Xenopus* (Silurana) tropicalis. *Comp med*. 2021;71, 512-520, doi:10.30802/aalas-cm-21-000061.
42. Kondo, Y, Iwamoto, R, Takahashi, T et al. Diversity of cortical bone morphology in anuran amphibians. *Dev Growth Differ*. 2023;65, 16-22, doi:10.1111/dgd.12831.
43. Paluh, DJ, Riddell, K, Early, CM et al. Rampant tooth loss across 200 million years of frog evolution. *Elife*. 2021;10, doi:10.7554/eLife.66926.
44. Paluh, DJ, Dillard, WA, Stanley, EL, Fraser, GJ, Blackburn, DC. Re-evaluating the morphological evidence for the re-evolution of lost mandibular teeth in frogs. *Evolution*. 2021;75, 3203-3213, doi:10.1111/evo.14379.
45. Jandausch, K, Schwarz, D, Bock, BL, Lukas, P. A decharming metamorphosis: The larval and adult morphology of the common spadefoot toad, *Pelobates fuscus*. *Zoologischer Anzeiger*. 2022;296, 37-49, doi:10.1016/j.jcz.2021.11.005.
46. Chang, L, Zhang, M, Chen, Q et al. From Water to Land: The Structural Construction and Molecular Switches in Lungs during Metamorphosis of *Microhyla fissipes*. *Biology (Basel)*. 2022;11, doi:10.3390/biology11040528.
47. Kraus, N, Metscher, B. Anuran development: A reinvestigation of the conus arteriosus and gill formation in Bufo bufo throughout metamorphosis using micro-CT. *Anat Rec (Hoboken)*. 2022;305, 1100-1111, doi:10.1002/ar.24766.
48. Zhu, W, Chang, L, Zhao, T, Wang, B, Jiang, J. Remarkable metabolic reorganization and altered metabolic requirements in frog metamorphic climax. *Front Zool*. 2020;17, 30, doi:10.1186/s12983-020-00378-6.
49. Zikmund, T, Novotna, M, Kavkova, M et al. High-contrast differentiation resolution 3D imaging of rodent brain by X-ray computed microtomography. *J Instrum*. 2018;13, doi:10.1088/1748-0221/13/02/C02039.
50. Metscher, BD. MicroCT for comparative morphology: simple staining methods allow high-contrast 3D imaging of diverse non-mineralized animal tissues. *BMC Physiol*. 2009;9, 11, doi:10.1186/1472-6793-9-11.
51. Nieuwkoop, PD, Faber, J. *Normal Table of Xenopus Laevis (Daudin): A Systematical and Chronological Survey of the Development from the Fertilized Egg Till the End of Metamorphosis*. (Garland Pub., 1994). https://doi.org/10.1201/9781003064565
52. Zahn, N, James-Zorn, C, Ponferrada, VG et al. Normal Table of *Xenopus* development: a new graphical resource. *Development*. 2022;149, doi:10.1242/dev.200356.
53. Gurdon, JB, Woodland, HR. in *Handbook of Genetics: Volume 4 Vertebrates of Genetic Interest* (ed Robert C. King) 35-50 (Springer US, 1975). https://doi.org/10.1007/978-1-4613-4470-4
54. Laznovsky J, Kavkova M, Reis A, Robovska-Havelkova P, Maia La, Krivanek J, Zikmund T, Kaiser J, Buchtova M, Harnos J. Supporting data for "Unveiling Vertebrate Development Dynamics in Frog *Xenopus laevis* using Micro-CT Imaging" *GigaScience Database*. 2024, https://doi.org/10.5524/102534.
55. Grant, S, Keating, MJ. Ocular migration and the metamorphic and postmetamorphic maturation of the retinotectal system in *Xenopus laevis*: an autoradiographic and morphometric study. *Development*. 1986;92, 43-69, doi:10.1242/dev.92.1.43.
56. Heath, JE, Northcutt, RG, Barber, RP. Rotational optokinesis in reptiles and its bearing on pupillary shape. *Zeitschrift für vergleichende Physiologie*. 1969;62, 75-85, doi:10.1007/BF00298043.
57. Herrel, A, Gonwouo, LN, Fokam, EB, Ngundu, WI, Bonneaud, C. Intersexual differences in body shape and locomotor performance in the aquatic frog, *Xenopus tropicalis*. 2012;287, 311-316, doi:10.1111/j.1469-7998.2012.00919.x.
58. Udin, SB. The instructive role of binocular vision in the *Xenopus* tectum. *Biological Cybernetics*. 2007;97, 493-503, doi:10.1007/s00422-007-0188-7.
59. Davit-Beal, T, Chisaka, H, Delgado, S, Sire, JY. Amphibian teeth: current knowledge, unanswered questions, and some directions for future research. *Biol Rev Camb Philos Soc*. 2007;82, 49-81, doi:10.1111/j.1469-185X.2006.00003.x.

60. Gonzalez Lopez, M, Huteckova, B, Lavicky, J et al. Spatiotemporal monitoring of hard tissue development reveals unknown features of tooth and bone development. *Sci Adv*. 2023;9, eadi0482, doi:10.1126/sciadv.adi0482.
61. Brink, KS, Henriquez, JI, Grieco, TM et al. Tooth Removal in the Leopard Gecko and the de novo Formation of Replacement Teeth. *Front Physiol*. 2021;12, 576816, doi:10.3389/fphys.2021.576816.
62. Nauwelaerts, S, Aerts, P. Two distinct gait types in swimming frogs. *J Zool*. 2002;258, 183-188, doi:10.1017/S0952836902001292.
63. Li, M, Gao, Z, Wang, J et al. Cooperation behavior of fore- And hindlimbs during jumping in *Rana dybowskii* and *Xenopus laevis*. *Ecology and evolution*. 2021;11, 7569-7578, doi:10.1002/ece3.7589.
64. Trueb, L, Hanken, J. Skeletal development in *Xenopus laevis* (Anura: Pipidae). *J Morphol*. 1992;214, 1-41, doi:10.1002/jmor.1052140102.
65. Chalmers, AD, Slack, JM. Development of the gut in *Xenopus laevis*. *Dev Dyn*. 1998;212, 509-521, doi: 10.1002/(SICI)1097-0177(199808)212:4<509::AID-AJA4>3.0.CO;2-L
66. Schreiber, AM, Cai, L, Brown, DD. Remodeling of the intestine during metamorphosis of *Xenopus laevis*. *Proc Natl Acad Sci U S A*. 2005;102, 3720-3725, doi:10.1073/pnas.0409868102.
67. Akinaga, K, Azumi, Y, Mogi, K, Toyoizumi, R. Stage-dependent sequential organization of nascent smooth muscle cells and its implications for the gut coiling morphogenesis in *Xenopus* larva. *Zoology (Jena, Germany)*. 2021;146, 125905, doi:10.1016/j.zool.2021.125905.
68. Kastner, DB, Kharazia, V, Nevers, R et al. Scalable method for micro-CT analysis enables large scale quantitative characterization of brain lesions and implants. *Sci Rep*. 2020;10, 20851, doi:10.1038/s41598-020-77796-3.
69 Abu-Daya, A, Godwin, A. CRISPR/Cas9 Gene Disruption Studies in F(0) *Xenopus* Tadpoles: Understanding Development and Disease in the Frog. *Methods in molecular biology*. 2023;2633, 111-130, doi:10.1007/978-1-0716-3004-4_10.

Figure 1

Click here to access/download;Figure;Figure_1.bmp

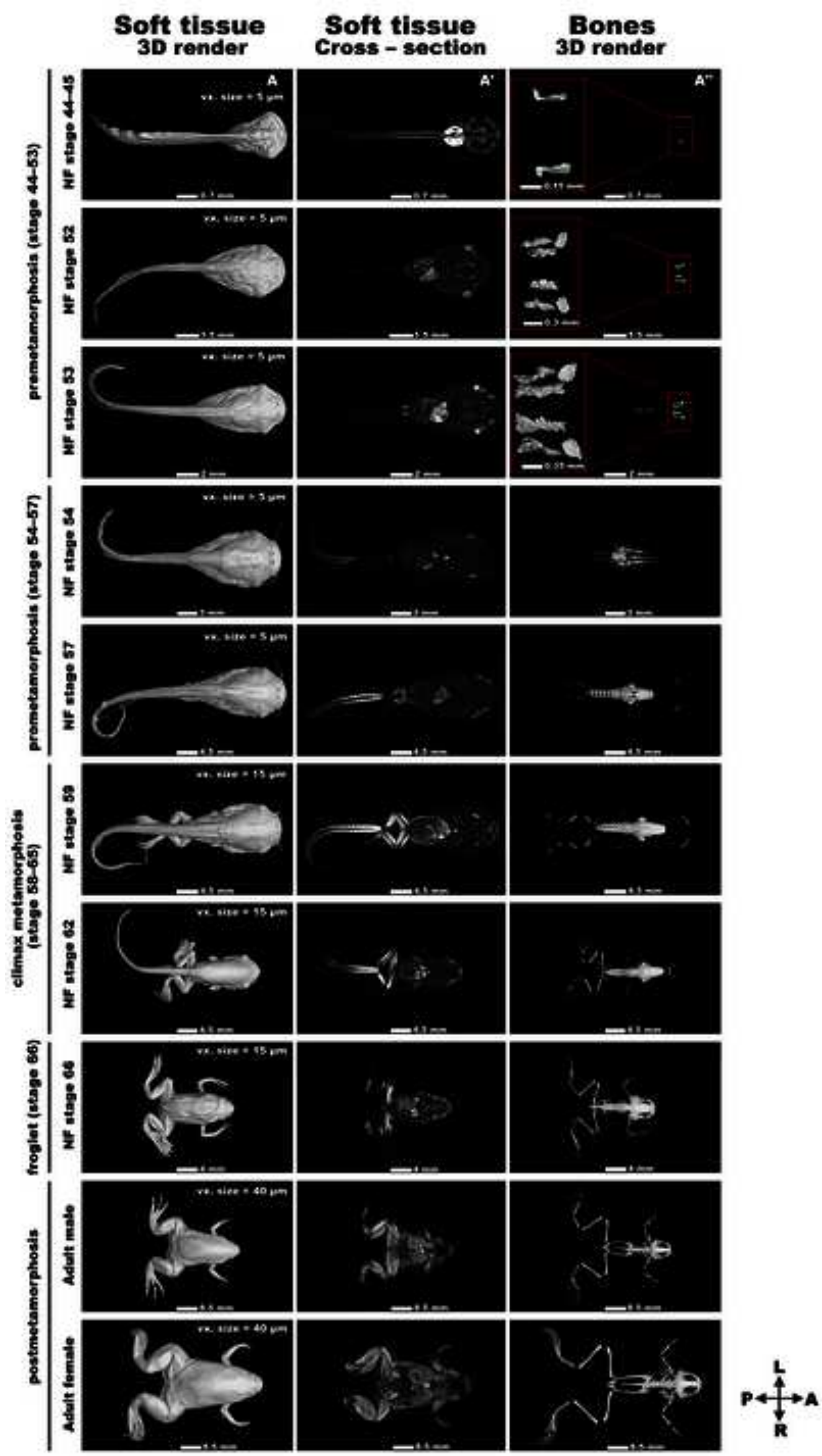

Figure 2

Click here to access/download;Figure;Figure_2.bmp

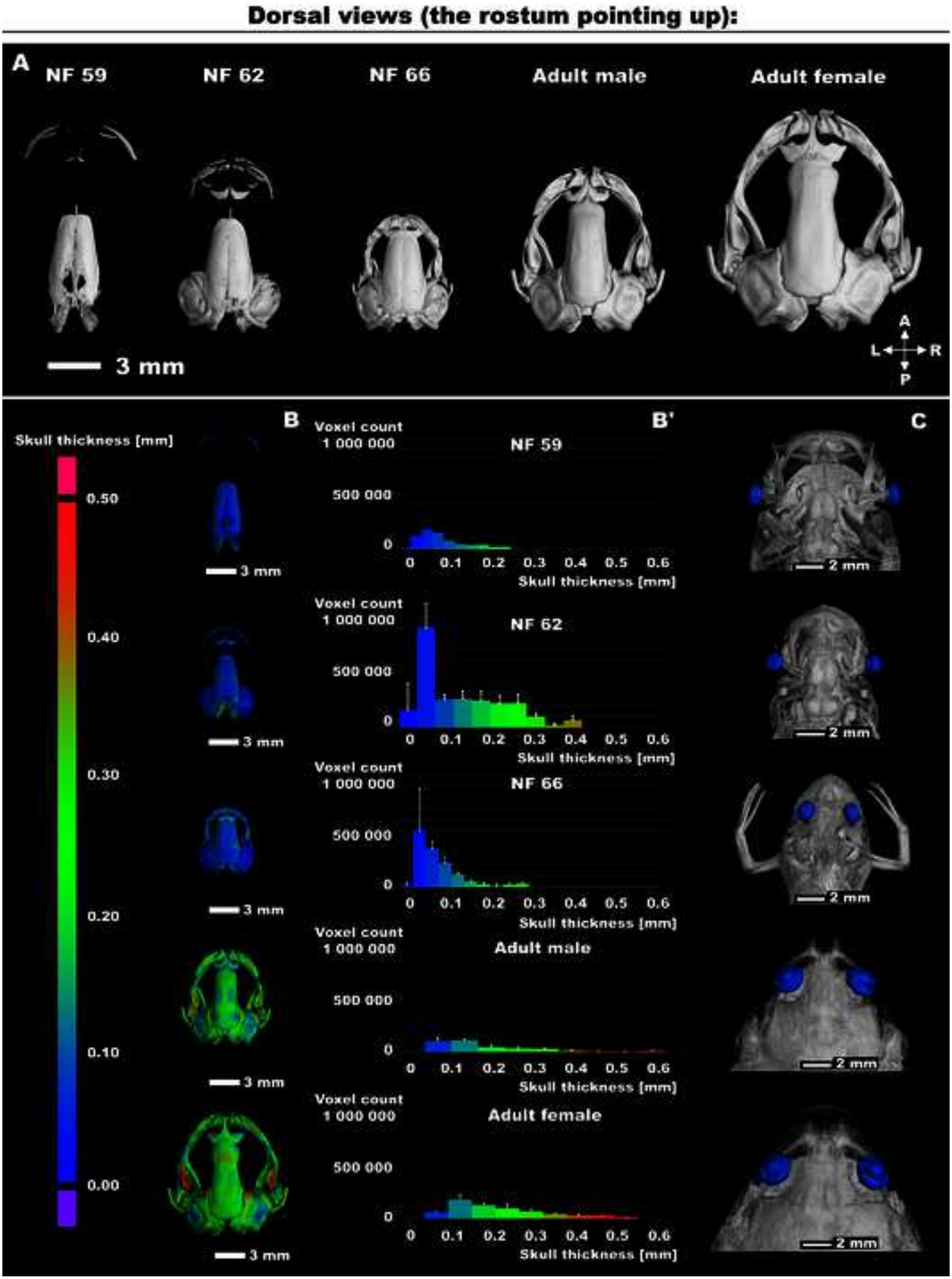

Figure 3

Click here to access/download;Figure;Figure_3.bmp

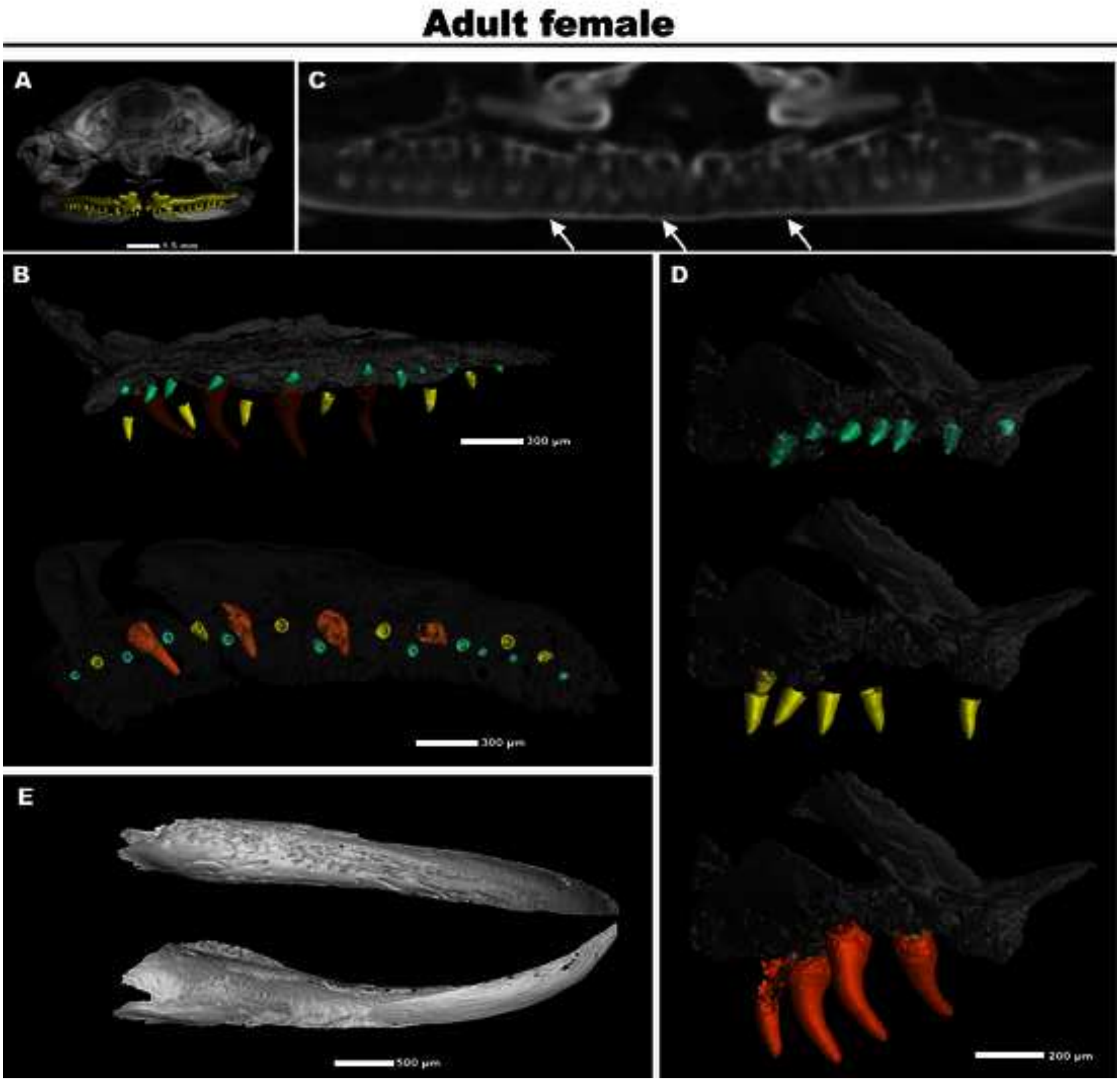

Figure 4

Click here to access/download;Figure;Figure_4.bmp

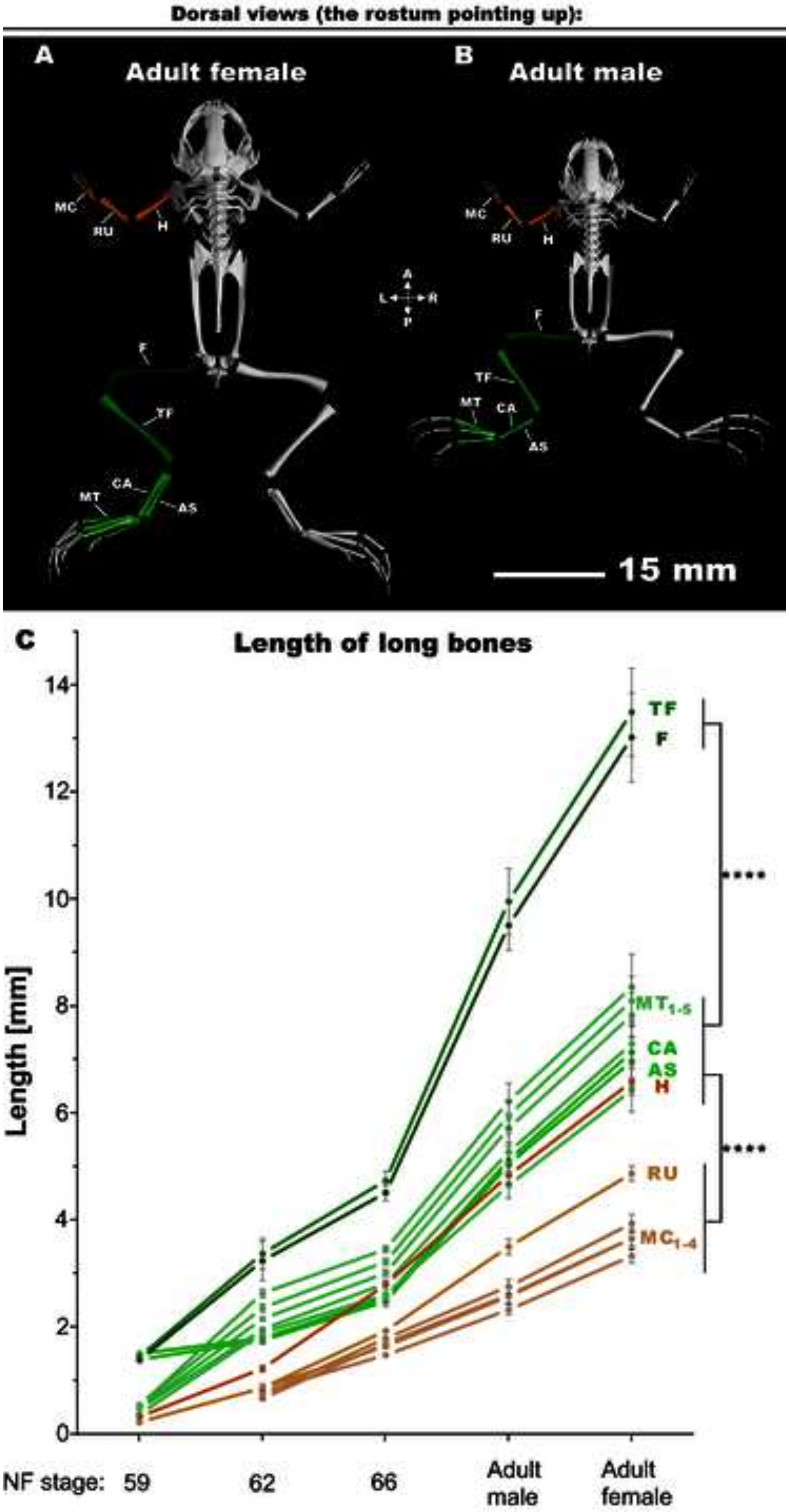

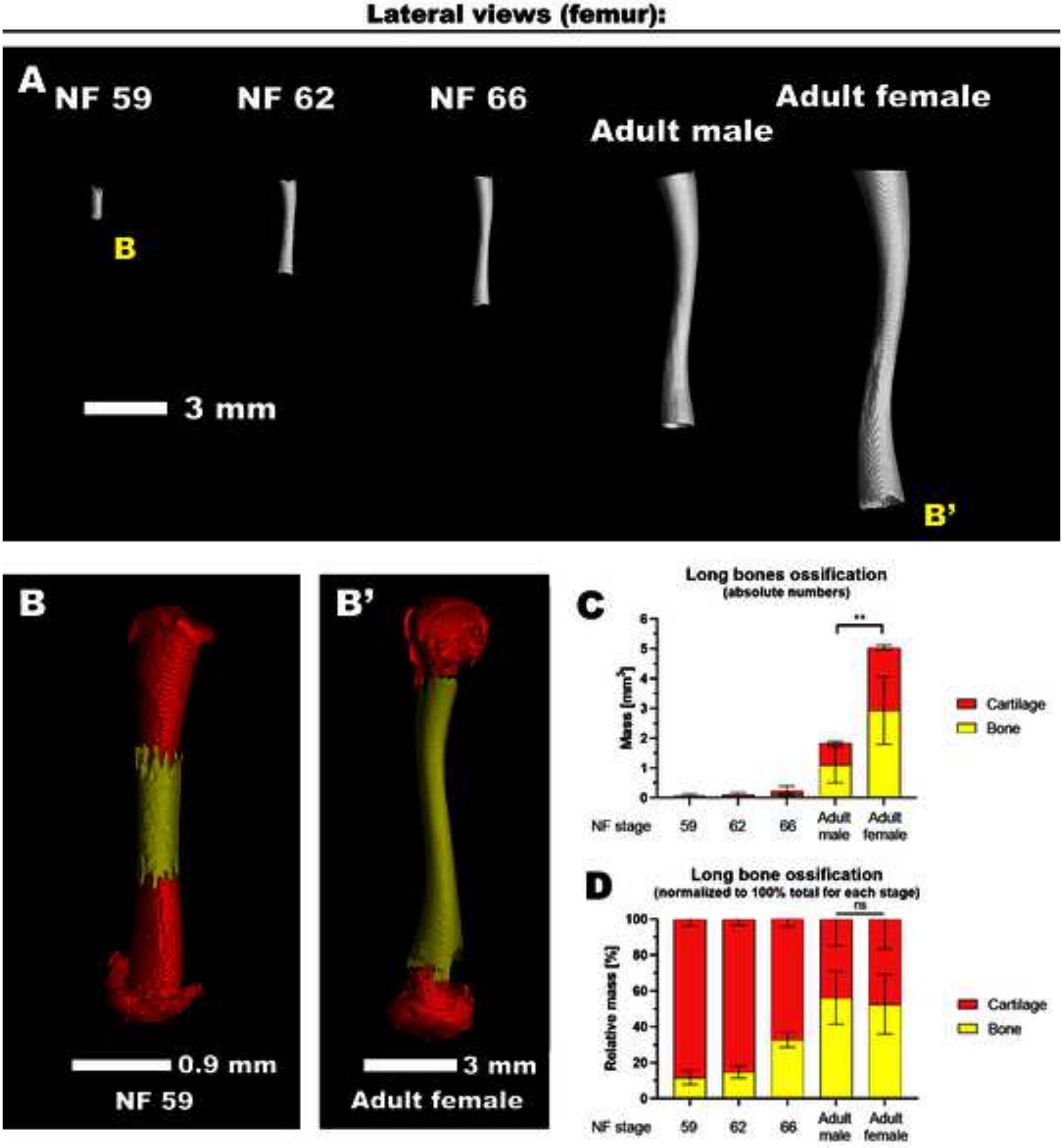
Lateral views (femur):
A
NF 59
NF 62
NF 66
Adult male
Adult female
B
B'
3 mm
B
0.9 mm
NF 59
B'
3 mm
Adult female
C
Long bones ossification
(absolute numbers)
Mass [mm³]
**
Cartilage
Bone
NF stage
59
62
66
Adult male
Adult female
D
Long bone ossification
(normalized to 100% total for each stage)
Relative mass [%]
ns
Cartilage
Bone
NF stage
59
62
66
Adult male
Adult female

Figure 6

Click here to access/download;Figure;Figure_6.bmp

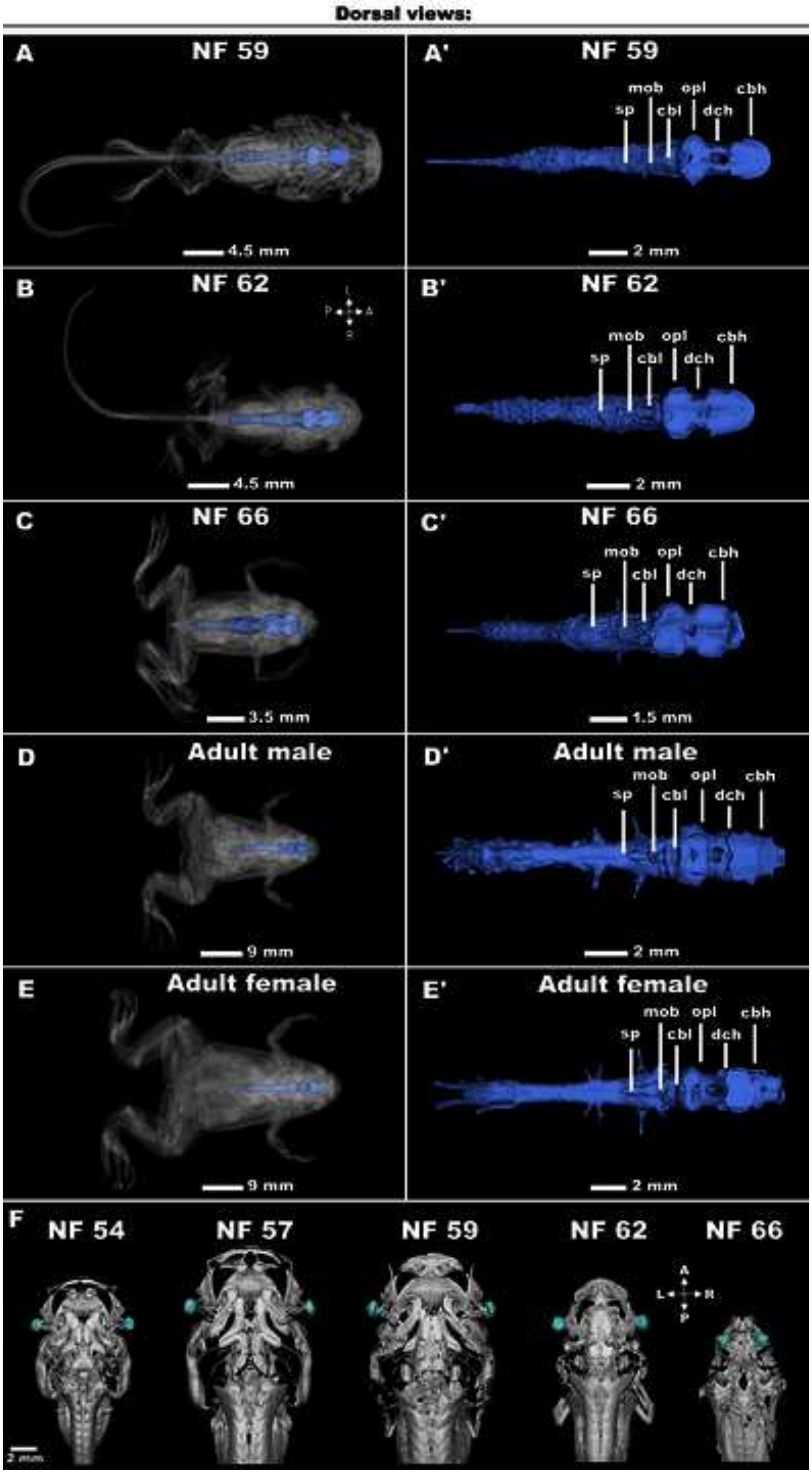

Figure 7

Click here to access/download;Figure;Figure_7.bmp

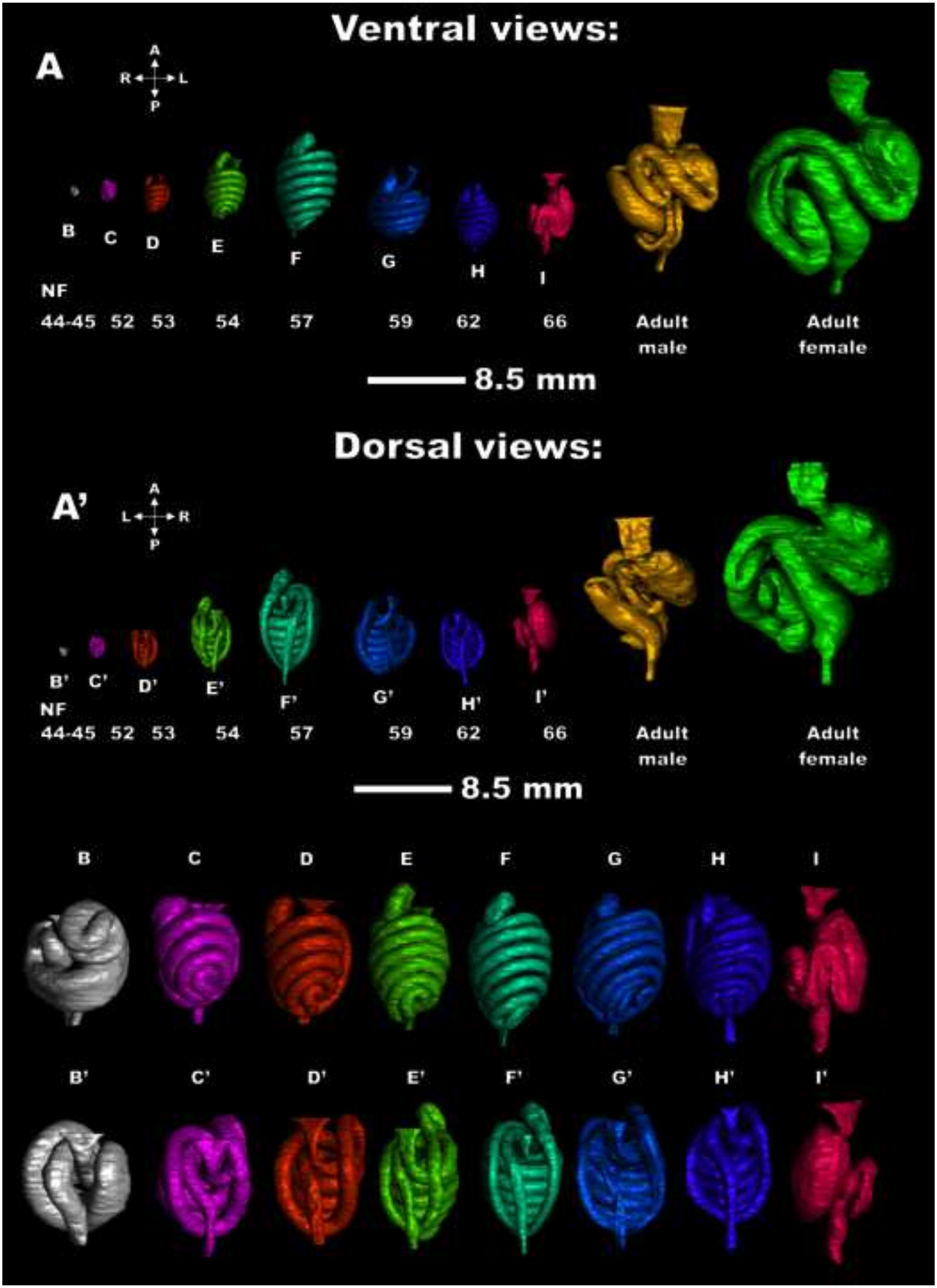

Supplementary Video 1

Click here to access/download
Supplementary Material
Supplementary Video 1.wmv

Supplementary Video 2

Click here to access/download
Supplementary Material
Supplementary Video 2.wmv

Supplementary Video 3

Click here to access/download

Supplementary Material

Supplementary Video 3.wmv

Supplementary Video 4

Click here to access/download
Supplementary Material
Supplementary Video 4.wmv

Supplementary Video 5

Click here to access/download
Supplementary Material
Supplementary_Video_5.wmv

Jakub Harnos, Ph.D.
Masaryk University, Faculty of Science, Kamenice 5,
Brno, Czech Republic, 62500
harnos@sci.muni.cz

April 9, 2024

GigaScience Editorial Office GigaScience Journal

Dear Editors of GigaScience,

I am resubmitting the revised manuscript titled "Unveiling Vertebrate Development Dynamics in Frog *Xenopus laevis* using Micro-CT Imaging" for your consideration. We appreciate the feedback provided by the reviewers, which has significantly improved the quality of our study.

In response to reviewer comments, we have enhanced the manuscript by increasing the number of repeats for each *Xenopus* sample and segmenting *Xenopus* guts, improving the rigor and comprehensiveness of our study.

Additionally, the Zenodo database containing the *Xenopus* atlas has been updated, and you can now access it via the DOI 10.5281/zenodo.10214561 or this link: https://zenodo.org/records/10951411?token=eyJhbGciOiJIUzUxMiJ9.eyJpZCI6IjI4MWU4ZGM2LTZiNWYtNDNhYi1hMmRhLTY4Y2I0YzVhM2I1MCIsImRhdGEiOnt9LCJyYW5kb20iOiI0ZTlmMDU0ZmI0N2ZkOTRhOGIxNjYyZmVlZDU5NDQ4NSJ9.DXIxirYhLz75m3KA_ydixIxWLur9PZPlfGBijC-Lj6uGAdNZfO8YaK3lgXhWWS0YaAndPHb17HIGeO36KSoW8Q

We have adhered to GigaScience's submission guidelines, and the manuscript has not been published elsewhere nor is it under consideration for publication elsewhere. All authors have reviewed and approved the revised manuscript, and we have no competing interests to declare.

Please feel free to contact me at harnos@sci.muni.cz for any further information or requirements for the submission process.

Thank you for considering our manuscript for publication in GigaScience. We look forward to contributing to your esteemed journal.

Sincerely,

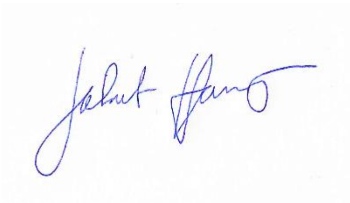

Assistant Professor Jakub Harnos, Ph.D.
Principal Investigator at Masaryk University
ORCID: 0000-0002-0752-9260

Response to reviewers
Click here to access/download;Personal Cover;Micro_CT_reviewer_reports_4.docx

Our responses to Reviewers (and the Editor at the end of this file) are highlighted in grey color.

Reviewer #1:

The authors present a set of 3D images of selected developmental stages of the widely-used laboratory model Xenopus laevis along with some examples of how the data might be used in developmental analyses. The dataset covers stages from mid-larva through metamorphosis to adult, which should provide a starting point for various studies of morphological development. Some studies will undoubtedly require other stages or more detailed images, but the presented data were collected with straightforward methods that will allow compatibility with future work.

The data appear to be sound in the collection and curation. Data availability is made clear in the article, and the complete set will be publicly available in standard formats on the Zenodo repository. This should ensure full accessibility to anyone interested.

The article is well-organized and clearly written.
We'd like to express our gratitude to Reviewer #1 for dedicating the time and effort to reviewing our manuscript.

A few points about the methods could be clarified:
Was only one specimen per stage scanned?
Initially, we conducted a single repetition merely to demonstrate the possible analyses without intending to infer any significant biological relevance from it. However, based on the feedback from other reviewers and the editor, we increased the sample size to n=3, thereby reinforcing the validity of our initial suggestions. We trust that this addresses the concerns raised by Reviewer #1.

Specimens were dehydrated through an ethanol series and then stained with free iodine in 90% methanol, and then rehydrated back through ethanol. Why was methanol used for staining and not dehydration? It seems odd to switch alcohols back and forth without intermediate steps. This could have some effect on tissue shrinkage.
We appreciate the feedback regarding our choice of ethanol for dehydration and methanol as a solvent for iodine in our protocol. The decision to use ethanol was based on its non-toxic nature, allowing for safer handling on the bench and also, we have adapted this dehydration from commonly used histologic methods used for the processing of animal tissue samples. The reason behind using methanol as a solvent for the iodine staining solution is that methanol has a smaller molecule than ethanol and thus the staining solution based on methanol better permeates the tissue. This method is commonly used in our collaborating research groups with great results (see the DOIs below this comment). We hope this clarification addresses any concerns and is satisfactory for the review process.
doi.org/10.1038/s41598-021-84579-x
doi.org/10.1088/1748-0221/13/02/C02039
doi.org/10.26508/lsa.202302073
doi.org/10.1038/s41467-023-39373-w

It should be indicated that the X-ray source target is tungsten (even though it is unlikely to be anything else in this machine).
Yes, we added this information into the manuscript, as requested (p. 8):

It is important to note that the X-ray source target utilized in this study was tungsten.

The "real images" (p. 7) in Suppl. Fig. 1 should simply be called photographs - microCT images are real too.
Yes, we changed this information in the manuscript, as suggested (p. 7).

For the measurements of bone mass, is the cartilage itself actually visible in the microCT images?
We only observed the cartilage in stained data. It's worth noting that Fig. 5A is derived from non-stained data, while Fig. 5B is from stained data. Furthermore, in Fig. 5B, we separated the cartilage from bones, and this represents the merged data. We have included this description in the figure legend:

Additionally, it should be noted that in A), the cartilage is not visible, while in B), the stained data allows for clear separation of cartilage from bones. The merged data in Fig. 5B) emphasizes the isolated cartilage.

Also, we put some additional image regarding this matter into the Supplementary material, specifically Suppl. Fig. 5.

p. 13: "The dataset's diverse species representation..." What does this mean? It is only one species.
We thank reviewer for this note. We apologize for this typo and correct the sentence to: "The dataset's diverse specimens' representation..."(p. 14); ("diverse" in this context means tadpoles, froglet, adult frogs)

The limitations on the image data are not discussed. All images have limits to their useful resolution and contrast among components; this is not a weakness, just a reality of imaging.
We acknowledge the reviewer's insightful comment regarding the limitations on the image data. In order to answer this point, we added the following paragraph to our manuscript (p. 9):

Image data inherently exhibits limitations in terms of useful resolution and contrast among components. These constraints are integral to the imaging process and are not indicative of weaknesses in our study, but rather intrinsic characteristics of the imaging modality employed. It is essential to acknowledge that, in any imaging technique, there are practical boundaries to the level of detail and contrast achievable.

The different reconstructed voxel sizes for different size specimens are mentioned, but it might be helpful to indicate the voxel sizes in Figure 1 as well as in the relevant table.
The table containing voxel size details for each NF stage is provided in the manuscript as Supplementary Table 1. Furthermore, we acknowledge the omission of the link in the text. To address this, we have added the link to Suppl. Table 1 into the text (p. 7). Additionally, we have included the voxel size values in Figure 1 for further clarity.

And if the middle column of Figure 1 could be published with full resolution of the snapshots it would help show the actual quality of the images.
As part of the revision process, all figures have been uploaded as individual high-quality files. Consequently, all figures now have sufficient resolution.

Reviewer #2:

The manuscript is well written and easy to understand. It will be a good contribution to the Xenopus research community as well as a useful reference for the field of developmental and amphibian biology.

We want to extend our appreciation to Reviewer #2 for the time and effort invested in reviewing our manuscript.

I suggest the following revisions:

- For the graphical abstract try alternating NF stage numbers above and below samples for a cleaner look, adult male and adult female can both remain at the top.

We appreciate the reviewer's suggestion and have incorporated this comment into the graphical abstract.

- Appreciate the rationale for providing the microCT analysis presented in this manuscript and choices of late-stage tadpoles, pre- and prometamorphosis, through metamporphosis to the adult male and female frog.

Thank you, we're grateful for it.

- For the head development section authors can make reference to the Xenhead drawings, Zahn et al. Development 2017.

In the mentioned section, we included the following statement (p. 11):

For more information about the X. laevis' head morphology, we also refer to the handmade drawings by Zahn and colleagues (Zahn, James-Zorn et al. 2022).

- Head Development section paragraph 4, change word from "gender" to "sex."

Certainly, we've updated this information in the manuscript as advised (page 9).

- Supplementary Table 3. Change "gender-related" to "sex-related."

Yes, we've made the suggested change in the manuscript (p. 28).

- Micro-CT Data Analysis of Long Bone Growth Dynamics section paragraph 1 change "in terms of gender" to "in terms of sex."

We have made the necessary adjustment in the manuscript as advised (p. 10).

- Figure 4 panels A and B don't reflect the observation that adult females are enlarged males. While the authors state that the view of the male and female skeletons are maximized and not proportional as stated in the caption, suggest that scale bars be employed and the images adjusted to show the size relationship difference between the sexes as in Figure 1. On first glance and perhaps to those not as familiar with the difference in sex size in Xenopus that in this particular example of the adult male image being more spread out compared to the image of the female, it feels misleading.

We acknowledge the concern regarding the representation of adult male and female skeletons in Figure 4, panels A and B. To address this, we incorporated one scale bar and adjusted the images to better reflect the actual size contrast.

- Ossification Analysis section paragraph 2 change "frog's gender" to "frog's sex."

Yes, we changed this information in the manuscript, as suggested (p. 11).

- Figure 5 panel A, the label is overlapping "NF 59." For panels B and B' scale bars on these panels would help the reader understand the proportions. Yes, there is the 3mm scale bar from panel A and as stated in the caption, but including them in the B panels could help even if panel B had a scale bar labeled at 0.25 mm and panel B' was 3 mm.

We have implemented all the necessary adjustments in Fig. 5A and B, as per your request.

- Segmentation of Selected Internal Soft Organ section, perhaps more commentary on the ability to observe the development of the segmentation of the brain regions: cbh: cerebral hemispheres; cbl: cerebellum; dch: diencephalon; mob: medulla oblongata; opl: optic lobes; sp: spinal cord while clearly shown in Figure 6, some accompanying description in the text would help readers in general or give the implication that microCT analysis of mutant or diseased frogs could help identify physical characteristics of frogs with developmental or neurological disorders. This would help transition from the analysis of a specific organ to the next section Further Biological Potential of Xenopus's Data.

We implemented these paragraphs into the main text:
Page 13: It is noteworthy that our micro-CT data allows for detailed observation of the developmental segmentation of various regions of the brain, which is very hard to dissect, especially from Xenopus adults (personal observation). Specifically, ***Fig. 6A-E**, **A'-E'**, and **Suppl. Video 4*** *provide a clear morphological visualization of the individual brain areas such as cerebral hemispheres (cbh), cerebellum (cbl), diencephalon (dch), medulla oblongata (mob), optic lobes (opl), and spinal cord (sp), in the tadpole and adult frog brains, as well as to follow how these structures are developing in time course. For more information about the X. laevis' brain and its detailed description, including its regeneration in developing tadpoles, we refer readers to the recent publication (Ishii, Yoshida et al. 2023).*

Page 15: Acknowledging the significance of our micro-CT data, it is also possible to address the common limitations linked to the absence of three-dimensional structural information about organs, such as the brain. The potential application of micro-CT, particularly in the study of mutants or diseased frogs, can offer valuable insights into the physical characteristics associated with developmental or neurological disorders. This not only holds true for Xenopus but also presents potential implications for humans. Notably, similar micro-CT-based studies involving, for instance, rat brains further underscore the relevance of our assumption (Kastner, Kharazia et al. 2020). Alternatively, the integration of micro-CT data with the Crispr/Cas9 system can be employed for disease modeling, as demonstrated in the recent study involving Xenopus tadpoles (Abu-Daya and Godwin 2023).

- These analyses, while thorough accompanied by novel visuals, require statistical implementation of multiple tadpoles and frogs per NF stage to account for variation in samples and to bolster the claims stated in skull thickness, the head mass and eye distance changes, increased length of the long bones during maturation, and femural ossification cartilage to bone ratios. This may constitute a suggested major revision to perform these analyses.

Certainly, we appreciate the reviewer's attention to the statistical robustness of our analysis. We want to assure you that we have now included a sufficient number of samples to support our findings. In Fig 2B', 4C, and 5C+D, we present the relevant data to underscore the trends observed in characteristics like skull thickness, increased length of long bones during maturation, and ossification cartilage to bone ratios. Moreover, to streamline the presentation, Graph 2D has been relocated to the Supplementary material. We trust that this addresses the concern raised by Reviewer #2.

Reviewer #3:

Laznovsky et al. present a nice compendium of micro-CT-based digital volumes of several stages of Xenopus development. Given the prominence of this important model animal in studies of developmental biology and physiology, this dataset is quite useful and will be of interest to the community. That said, the study has some key limitations that will limit its utility for the research community, though these do not reduce the dataset's impact in the education and popular science realms, which is also a stated goal for the paper. Overall, I recommend publication after an effort has been made to address the following concerns.
We also want to convey our gratitude to Reviewer #3 for the valuable time and effort in reviewing our manuscript.

1. The atlas adequately samples developmental stages from late tadpole through metamorphosis. However, as far as I can tell only a single sample has been imaged at each stage. Thus, the quantifications of inter-stage differences shown here (Fig. 2, 4, 5) are at best very rough estimates and also provide no information about intra-stage variability in these metrics. This is not a fatal weakness, but it is an important caveat that I believe should be very explicitly stated in the text and in the figure legend of relevant figures.
We conducted all requisite experiments to reproduce the data from Fig. 2, 4, and 5, as per your request. Please refer to the respective figures for detailed information. We trust that this addresses the concern raised by Reviewer #3.

2. I am very disappointed that the rich history of microCT on Xenopus seems to have been entirely ignored by these authors. MicroCT has already been used to describe the skull, the brain, liver, blood vessels, etc. during Xenopus development. (Just a few papers the authors should read are: Slater et al., PLoS One 2009; Senevirathnea et al., PNAS, 2019; Ishii et al., Dev. Growth, Diff. 2023; Zhu et al., Front. Zool 2020.) It has also been used for comparative studies of other frogs (Kondo et al., Dev. Growth, Diff. 2022; Kraus, Anat. Rec. 2021; Jandausch et al., Zool. Anz. 2022; Paluh, et al., Evolution 2021, Paluh et al., eLife 2021). None of these -or the many other relevant papers- are discussed or cited here. The research community would be much better served if authors make a serious effort to integrate their methods and their results into this existing literature.

We regret any disappointment caused to the reviewer and would like to clarify the rationale behind this apparent misunderstanding. While we acknowledge the publications mentioned by the reviewer, our original intent in the manuscript was to emphasize that the micro-CT technique had not been applied at the whole-organism level in Xenopus, except for a single instance (Porro and Richards, J Anat, 2017). However, we now concur with the reviewer's perspective and have made a concerted effort to align our methods and results more closely with the existing literature (p. 4):

In response to these limitations, a detailed description of an adult male frog's anatomy (as a complete organism) using a non-destructive micro-CT method was recently provided (Porro and Richards 2017). Micro-CT, to be candid, has already been employed to some extent as a valuable tool in selected stages of X. laevis' embryos, tadpoles, and frogs to explore localized or specific occurrences, such as gastrulation (Moosmann, Ershov et al. 2014), craniofacial (Slater, Liu et al. 2009) and urostyle (Senevirathne, Baumgart et al. 2020) development, skeletal morphology (Vasquez, Hansen et al. 2008, Matthews and du Plessis 2016), limb (skeletal) regeneration (Feng, Milner et al. 2011, Chen, Lin et al. 2012, Golding, Guay et al. 2016), along with brain regeneration (Ishii, Yoshida et al. 2023), and the anatomy of cranial and anterior spinal nerves (Naumann and Olsson 2018), the head *(Descamps, Sochacka et al. 2020, Murugan, Vigran et al. 2022), and several internal organs (Descamps, Sochacka et al. 2014). Besides X. laevis itself,*

micro-CT has been utilized in closely related frogs to investigate, for instance, chondrocranium organization in A. obstetricans (Krings, Muller et al. 2017), skeleton (Herrel and Bonneaud 2012) and digit (Zhang, Wilson et al. 2021) morphology in X. tropicalis, cortical bone morphology in various anuran amphibians (Kondo, Iwamoto et al. 2023), re-evolution of lost mandibular teeth in G. guentheri *(Paluh, Dillard et al. 2021, Paluh, Riddell et al. 2021), together with cranial musculoskeletal structures in P. fuscus (Jandausch, Schwarz et al. 2022), lungs rearrangements during metamorphosis in M. fissipes (Chang, Zhang et al. 2022), gill formation during metamorphosis in B. bufo (Kraus and Metscher 2022), and metabolic reorganization in metamorphic R. omeimontis tadpoles (Zhu, Chang et al. 2020). Nevertheless, the spatiotemporal dynamics of X. laevis' late development on the level of a whole organism, along with the comparison between adult male and female frogs, remain largely unexplored in sufficient detail.*

3. An opportunity may have been missed here to provide some truly new biological insights: The gut remodels substantially during metamorphosis, but to my knowledge that has NOT been previously examined by microCT. It may not work, as the gut may simply be too soft to visualize, but then again, it may be worth trying.

We appreciate the reviewer's suggestion and have exerted considerable effort to provide further insights into the development of the Xenopus gut using our samples (refer to p. 14 and Figure 7).

EDITOR'S COMMENT:

In addition, please register any new software application in the bio.tools and SciCrunch.org databases to receive RRID (Research Resource Identification Initiative ID) and biotoolsID identifiers, and include these in your manuscript. Computational workflows should be registered in workflowhub.eu and the DOIs cited in the relevant places in the manuscript. These will facilitate tracking, reproducibility and re-use of your tool.

As for RRIDs, we implemented this sentence into the manuscript (p. 7):

The reconstructed data were imported into VG Studio MAX 2023.4 software (Volume Graphics GmbH, Heidelberg, Germany), in which the measured data were segmented, analyzed, and visualized using VG Studio MAX, RRID: SCR_017997. The segmentation of Xenopus guts was done manually by an operator using the software Avizo 2020.2 (Thermo Fisher Scientific, Waltham, MA, USA) (Avizo 3D Software, RRID: SCR_014431).

List of publications used in this file:

Abu-Daya, A. and A. Godwin (2023). "CRISPR/Cas9 Gene Disruption Studies in F(0) Xenopus Tadpoles: Understanding Development and Disease in the Frog." Methods Mol Biol **2633**: 111-130.

Chang, L., M. Zhang, Q. Chen, J. Liu, W. Zhu and J. Jiang (2022). "From Water to Land: The Structural Construction and Molecular Switches in Lungs during Metamorphosis of Microhyla fissipes." Biology (Basel) **11**(4).

Chen, Y., G. Lin, Y. Chen, A. Fok and J. M. Slack (2012). "Micro-computed tomography for visualizing limb skeletal regeneration in young Xenopus frogs." Anat Rec (Hoboken) **295**(10): 1562-1565.

Descamps, E., A. Sochacka, B. De Kegel, D. Van Loo, L. Van Hoorebeke and D. J. B. J. o. Z. Adriaens (2014). "Soft tissue discrimination with contrast agents using micro-CT scanning." **144**(1).

Descamps, E., A. Sochacka, B. D. Kegel, D. V. Loo, L. V. Hoorebeke and D. J. B. J. o. Z. Adriaens (2020). "Soft tissue discrimination with contrast agents using micro-CT scanning." **144**: 20-40.

Feng, L., D. J. Milner, C. Xia, H. L. Nye, P. Redwood, J. A. Cameron, D. L. Stocum, N. Fang and I. Jasiuk (2011). "Xenopus laevis as a novel model to study long bone critical-size defect repair by growth factor-mediated regeneration." Tissue Eng Part A **17**(5-6): 691-701.

Golding, A., J. A. Guay, C. Herrera-Rincon, M. Levin and D. L. Kaplan (2016). "A Tunable Silk Hydrogel Device for Studying Limb Regeneration in Adult Xenopus Laevis." PLoS One **11**(6): e0155618.

Herrel, A. and C. Bonneaud (2012). "Temperature dependence of locomotor performance in the tropical clawed frog, Xenopus tropicalis." Journal of Experimental Biology **215**(14): 2465-2470.

Ishii, R., M. Yoshida, N. Suzuki, H. Ogino and M. Suzuki (2023). "X-ray micro-computed tomography of Xenopus tadpole reveals changes in brain ventricular morphology during telencephalon regeneration." Dev Growth Differ **65**(6): 300-310.

Jandausch, K., D. Schwarz, B. L. Bock and P. Lukas (2022). "A decharming metamorphosis: The larval and adult morphology of the common spadefoot toad, Pelobates fuscus." Zoologischer Anzeiger **296**: 37-49.

Kastner, D. B., V. Kharazia, R. Nevers, C. Smyth, D. A. Astudillo-Maya, G. M. Williams, Z. Yang, C. M. Holobetz, L. D. Santina, D. Y. Parkinson and L. M. Frank (2020). "Scalable method for micro-CT analysis enables large scale quantitative characterization of brain lesions and implants." Sci Rep **10**(1): 20851.

Kondo, Y., R. Iwamoto, T. Takahashi, K. Suganuma, H. Kato, H. Nakamura and A. Yukita (2023). "Diversity of cortical bone morphology in anuran amphibians." Dev Growth Differ **65**(1): 16-22.

Kraus, N. and B. Metscher (2022). "Anuran development: A reinvestigation of the conus arteriosus and gill formation in Bufo bufo throughout metamorphosis using micro-CT." Anat Rec (Hoboken) **305**(5): 1100-1111.

Krings, M., H. Muller, M. J. Heneka and D. Rodder (2017). "Modern morphological methods for tadpole studies. A comparison of micro-CT, and clearing and staining protocols modified for frog larvae." Biotech Histochem **92**(8): 595-605.

Matthews, T. and A. J. P. E. du Plessis (2016). "Using X-ray computed tomography analysis tools to compare the skeletal element morphology of fossil and modern frog (Anura) species." **19**(1): 1-46.

Moosmann, J., A. Ershov, V. Weinhardt, T. Baumbach, M. S. Prasad, C. LaBonne, X. Xiao, J. Kashef and R. J. N. P. Hofmann (2014). "Time-lapse X-ray phase-contrast microtomography for in vivo imaging and analysis of morphogenesis." **9**(2): 294-304.

Murugan, N. J., H. J. Vigran, K. A. Miller, A. Golding, Q. L. Pham, M. M. Sperry, C. Rasmussen-Ivey, A. W. Kane, D. L. Kaplan and M. Levin (2022). "Acute multidrug delivery via a wearable bioreactor facilitates long-term limb regeneration and functional recovery in adult Xenopus laevis." Sci Adv **8**(4): eabj2164.

Naumann, B. and L. Olsson (2018). "Three-dimensional reconstruction of the cranial and anterior spinal nerves in early tadpoles of Xenopus laevis (Pipidae, Anura)." J Comp Neurol **526**(5): 836-857.

Paluh, D. J., W. A. Dillard, E. L. Stanley, G. J. Fraser and D. C. Blackburn (2021). "Re-evaluating the morphological evidence for the re-evolution of lost mandibular teeth in frogs." Evolution **75**(12): 3203-3213.

Paluh, D. J., K. Riddell, C. M. Early, M. M. Hantak, G. F. Jongsma, R. M. Keeffe, F. Magalhaes Silva, S. V. Nielsen, M. C. Vallejo-Pareja, E. L. Stanley and D. C. Blackburn (2021). "Rampant tooth loss across 200 million years of frog evolution." Elife **10**.

Porro, L. B. and C. T. Richards (2017). "Digital dissection of the model organism Xenopus laevis using contrast-enhanced computed tomography." J Anat **231**(2): 169-191.

Senevirathne, G., S. Baumgart, N. Shubin, J. Hanken and N. H. Shubin (2020). "Ontogeny of the anuran urostyle and the developmental context of evolutionary novelty." Proc Natl Acad Sci U S A **117**(6): 3034-3044.

Slater, B. J., K. J. Liu, M. D. Kwan, N. Quarto and M. T. Longaker (2009). "Cranial osteogenesis and suture morphology in Xenopus laevis: a unique model system for studying craniofacial development." PLoS One **4**(1): e3914.

Vasquez, S. X., M. S. Hansen, A. N. Bahadur, M. F. Hockin, G. L. Kindlmann, L. Nevell, I. Q. Wu, D. J. Grunwald, D. M. Weinstein, G. M. J. T. A. R. A. i. I. A. Jones, E. B. A. i. I. Anatomy and E. Biology (2008). "Optimization of volumetric computed tomography for skeletal analysis of model genetic organisms." **291**(5): 475-487.

Zahn, N., C. James-Zorn, V. G. Ponferrada, D. S. Adams, J. Grzymkowski, D. R. Buchholz, N. M. Nascone-Yoder, M. Horb, S. A. Moody, P. D. Vize and A. M. Zorn (2022). "Normal Table of Xenopus development: a new graphical resource." Development **149**(14).

Zhang, M., S. S. Wilson, K. M. Casey, P. E. Thomson, A. L. Zlatow, V. S. Langlois and S. L. Green (2021). "Degenerative Osteoarthropathy in Laboratory Housed Xenopus (Silurana) tropicalis." Comp Med **71**(6): 512-520.

Zhu, W., L. Chang, T. Zhao, B. Wang and J. Jiang (2020). "Remarkable metabolic reorganization and altered metabolic requirements in frog metamorphic climax." Front Zool **17**: 30.
