# Supplementary material for: Unveiling vertebrate development dynamics in frog Xenopus laevis using micro-CT imaging
Source: Gigascience. 2024 Jul 17;13:giae037. doi: 10.1093/gigascience/giae037 (PMC12558079; doi:10.1093/gigascience/giae037)
Supplement: giae037_Response_to_Reviewer_Comments_Original_Submission [file giae037_Response_to_Reviewer_Comments_Original_Submission.pdf]

# Author's Response To Reviewer Comments

Our responses have been upload as a separate document. We are enclosing the text here:

Reviewer #1:
The authors present a set of 3D images of selected developmental stages of the widely-used laboratory model Xenopus laevis along with some examples of how the data might be used in developmental analyses. The dataset covers stages from mid-larva through metamorphosis to adult, which should provide a starting point for various studies of morphological development. Some studies will undoubtedly require other stages or more detailed images, but the presented data were collected with straightforward methods that will allow compatibility with future work.

The data appear to be sound in the collection and curation. Data availability is made clear in the article, and the complete set will be publicly available in standard formats on the Zenodo repository. This should ensure full accessibility to anyone interested.

The article is well-organized and clearly written.
We'd like to express our gratitude to Reviewer #1 for dedicating the time and effort to reviewing our manuscript.

A few points about the methods could be clarified:
Was only one specimen per stage scanned?
Initially, we conducted a single repetition merely to demonstrate the possible analyses without intending to infer any significant biological relevance from it. However, based on the feedback from other reviewers and the editor, we increased the sample size to n=3, thereby reinforcing the validity of our initial suggestions. We trust that this addresses the concerns raised by Reviewer #1.

Specimens were dehydrated through an ethanol series and then stained with free iodine in 90% methanol, and then rehydrated back through ethanol. Why was methanol used for staining and not dehydration? It seems odd to switch alcohols back and forth without intermediate steps. This could have some effect on tissue shrinkage.

We appreciate the feedback regarding our choice of ethanol for dehydration and methanol as a solvent for iodine in our protocol. The decision to use ethanol was based on its non-toxic nature, allowing for safer handling on the bench and also, we have adapted this dehydration from commonly used histologic methods used for the processing of animal tissue samples. The reason behind using methanol as a solvent for the iodine staining solution is that methanol has a smaller molecule than ethanol and thus the staining solution based on methanol better permeates the tissue. This method is commonly used in our collaborating research groups with great results (see the DOIs below this comment). We hope this clarification addresses any concerns and is satisfactory for the review process.
doi.org/10.1038/s41598-021-84579-x
doi.org/10.1088/1748-0221/13/02/C02039
doi.org/10.26508/lsa.202302073
doi.org/10.1038/s41467-023-39373-w

It should be indicated that the X-ray source target is tungsten (even though it is unlikely to be anything else in this machine).

Yes, we added this information into the manuscript, as requested (p. 8):
It is important to note that the X-ray source target utilized in this study was tungsten.

The "real images" (p. 7) in Suppl. Fig. 1 should simply be called photographs - microCT images are real too.

Yes, we changed this information in the manuscript, as suggested (p. 7).

For the measurements of bone mass, is the cartilage itself actually visible in the microCT images?

We only observed the cartilage in stained data. It's worth noting that Fig. 5A is derived from non-stained data, while Fig. 5B is from stained data. Furthermore, in Fig. 5B, we separated the cartilage from bones, and this represents the merged data. We have included this description in the figure legend:
Additionally, it should be noted that in A), the cartilage is not visible, while in B), the stained data allows for clear separation of cartilage from bones. The merged data in Fig. 5B) emphasizes the isolated cartilage.
Also, we put some additional image regarding this matter into the Supplementary material, specifically Suppl. Fig. 5.

p. 13: "The dataset's diverse species representation…" What does this mean? It is only one species.

We thank the reviewer for this note. We apologize for this typo and correct the sentence to: "The dataset's diverse specimens' representation…"(p. 14); ("diverse" in this context means tadpoles, froglet, adult frogs)

The limitations on the image data are not discussed. All images have limits to their useful resolution and contrast among components; this is not a weakness, just a reality of imaging.

We acknowledge the reviewer's insightful comment regarding the limitations on the image data. In order to answer this point, we added the following paragraph to our manuscript (p. 9):
Image data inherently exhibits limitations in terms of useful resolution and contrast among components. These constraints are integral to the imaging process and are not indicative of weaknesses in our study, but rather intrinsic characteristics of the imaging modality employed. It is essential to acknowledge that, in any imaging technique, there are practical boundaries to the level of detail and contrast achievable.

The different reconstructed voxel sizes for different size specimens are mentioned, but it might be helpful to indicate the voxel sizes in Figure 1 as well as in the relevant table.
The table containing voxel size details for each NF stage is provided in the manuscript as Supplementary Table 1. Furthermore, we acknowledge the omission of the link in the text. To address this, we have added the link to Suppl. Table 1 into the text (p. 7). Additionally, we have included the voxel size values in Figure 1 for further clarity.

And if the middle column of Figure 1 could be published with full resolution of the snapshots it would help show the actual quality of the images.
As part of the revision process, all figures have been uploaded as individual high-quality files.
Consequently, all figures now have sufficient resolution.

Reviewer #2:
The manuscript is well written and easy to understand. It will be a good contribution to the Xenopus research community as well as a useful reference for the field of developmental and amphibian biology.
We want to extend our appreciation to Reviewer #2 for the time and effort invested in reviewing our manuscript.

I suggest the following revisions:
- For the graphical abstract try alternating NF stage numbers above and below samples for a cleaner look, adult male and adult female can both remain at the top.
We appreciate the reviewer's suggestion and have incorporated this comment into the graphical abstract.

- Appreciate the rationale for providing the microCT analysis presented in this manuscript and choices of late-stage tadpoles, pre- and prometamorphosis, through metamporphosis to the adult male and female frog.
Thank you, we're grateful for it.

- For the head development section authors can make reference to the Xenhead drawings, Zahn et al.

Development 2017.
In the mentioned section, we included the following statement (p. 11):
For more information about the X. laevis' head morphology, we also refer to the handmade drawings by Zahn and colleagues (Zahn, James-Zorn et al. 2022).

- Head Development section paragraph 4, change word from "gender" to "sex."
Certainly, we've updated this information in the manuscript as advised (page 9).

- Supplementary Table 3. Change "gender-related" to "sex-related."
Yes, we've made the suggested change in the manuscript (p. 28).
- Micro-CT Data Analysis of Long Bone Growth Dynamics section paragraph 1 change "in terms of gender" to "in terms of sex."
We have made the necessary adjustment in the manuscript as advised (p. 10).

- Figure 4 panels A and B don't reflect the observation that adult females are enlarged males. While the authors state that the view of the male and female skeletons are maximized and not proportional as stated in the caption, suggest that scale bars be employed and the images adjusted to show the size relationship difference between the sexes as in Figure 1. On first glance and perhaps to those not as familiar with the difference in sex size in Xenopus that in this particular example of the adult male image being more spread out compared to the image of the female, it feels misleading.

We acknowledge the concern regarding the representation of adult male and female skeletons in Figure 4, panels A and B. To address this, we incorporated one scale bar and adjusted the images to better reflect the actual size contrast.

- Ossification Analysis section paragraph 2 change "frog's gender" to "frog's sex."
Yes, we changed this information in the manuscript, as suggested (p. 11).

- Figure 5 panel A, the label is overlapping "NF 59." For panels B and B' scale bars on these panels would help the reader understand the proportions. Yes, there is the 3mm scale bar from panel A and as stated in the caption, but including them in the B panels could help even if panel B had a scale bar labeled at 0.25 mm and panel B' was 3 mm.
We have implemented all the necessary adjustments in Fig. 5A and B, as per your request.

- Segmentation of Selected Internal Soft Organ section, perhaps more commentary on the ability to observe the development of the segmentation of the brain regions: cbh:
cerebral hemispheres; cbl: cerebellum; dch: diencephalon; mob: medulla oblongata; opl: optic lobes; sp: spinal cord while clearly shown in Figure 6, some accompanying description in the text would help readers in general or give the implication that microCT analysis of mutant or diseased frogs could help identify physical characteristics of frogs with developmental or neurological disorders. This would help transition from the analysis of a specific organ to the next section Further Biological Potential of Xenopus's Data.

We implemented these paragraphs into the main text:
Page 13: It is noteworthy that our micro-CT data allows for detailed observation of the developmental segmentation of various regions of the brain, which is very hard to dissect, especially from Xenopus adults (personal observation). Specifically, Fig. 6A-E, A'-E', and Suppl. Video 4 provide a clear morphological visualization of the individual brain areas such as cerebral hemispheres (cbh), cerebellum (cbl), diencephalon (dch), medulla oblongata (mob), optic lobes (opl), and spinal cord (sp), in the tadpole and adult frog brains, as well as to follow how these structures are developing in time course. For more information about the X. laevis' brain and its detailed description, including its regeneration in developing tadpoles, we refer readers to the recent publication (Ishii, Yoshida et al. 2023).
Page 15: Acknowledging the significance of our micro-CT data, it is also possible to address the common limitations linked to the absence of three-dimensional structural information about organs, such as the brain. The potential application of micro-CT, particularly in the study of mutants or diseased frogs, can offer valuable insights into the physical characteristics associated with developmental or neurological disorders. This not only holds true for Xenopus but also presents potential implications for humans. Notably, similar micro-CT-based studies involving, for instance, rat brains further underscore the relevance

of our assumption (Kastner, Kharazia et al. 2020). Alternatively, the integration of micro-CT data with the Crispr/Cas9 system can be employed for disease modeling, as demonstrated in the recent study involving Xenopus tadpoles (Abu-Daya and Godwin 2023).

- These analyses, while thorough accompanied by novel visuals, require statistical implementation of multiple tadpoles and frogs per NF stage to account for variation in samples and to bolster the claims stated in skull thickness, the head mass and eye distance changes, increased length of the long bones during maturation, and femural ossification cartilage to bone ratios. This may constitute a suggested major revision to perform these analyses.

Certainly, we appreciate the reviewer's attention to the statistical robustness of our analysis. We want to assure you that we have now included a sufficient number of samples to support our findings. In Fig 2B', 4C, and 5C+D, we present the relevant data to underscore the trends observed in characteristics like skull thickness, increased length of long bones during maturation, and ossification cartilage to bone ratios. Moreover, to streamline the presentation, Graph 2D has been relocated to the Supplementary material. We trust that this addresses the concern raised by Reviewer #2.

Reviewer #3:
Laznovsky et al. present a nice compendium of micro-CT-based digital volumes of several stages of Xenopus development. Given the prominence of this important model animal in studies of developmental biology and physiology, this dataset is quite useful and will be of interest to the community. That said, the study has some key limitations that will limit its utility for the research community, though these do not reduce the dataset's impact in the education and popular science realms, which is also a stated goal for the paper. Overall, I recommend publication after an effort has been made to address the following concerns.
We also want to convey our gratitude to Reviewer #3 for the valuable time and effort in reviewing our manuscript.

1. The atlas adequately samples developmental stages from late tadpole through metamorphosis. However, as far as I can tell only a single sample has been imaged at each stage. Thus, the quantifications of inter-stage differences shown here (Fig. 2, 4, 5) are at best very rough estimates and also provide no information about intra-stage variability in these metrics. This is not a fatal weakness, but it is an important caveat that I believe should be very explicitly stated in the text and in the figure legend of relevant figures.

We conducted all requisite experiments to reproduce the data from Fig. 2, 4, and 5, as per your request. Please refer to the respective figures for detailed information. We trust that this addresses the concern raised by Reviewer #3.

2. I am very disappointed that the rich history of microCT on Xenopus seems to have been entirely ignored by these authors. MicroCT has already been used to describe the skull, the brain, liver, blood vessels, etc. during Xenopus development. (Just a few papers the authors should read are: Slater et al., PLoS One 2009; Senevirathnea et al., PNAS, 2019; Ishii et al., Dev. Growth, Diff. 2023; Zhu et al., Front. Zool 2020.) It has also been used for comparative studies of other frogs (Kondo et al., Dev. Growth, Diff. 2022; Kraus, Anat. Rec. 2021; Jandausch et al., Zool. Anz. 2022; Paluh, et al., Evolution 2021, Paluh et al., eLife 2021). None of these -or the many other relevant papers- are discussed or cited here. The research community would be much better served if authors make a serious effort to integrate their methods and their results into this existing literature.

We regret any disappointment caused to the reviewer and would like to clarify the rationale behind this apparent misunderstanding. While we acknowledge the publications mentioned by the reviewer, our original intent in the manuscript was to emphasize that the micro-CT technique had not been applied at the whole-organism level in Xenopus, except for a single instance (Porro and Richards, J Anat, 2017). However, we now concur with the reviewer's perspective and have made a concerted effort to align our methods and results more closely with the existing literature (p. 4):

In response to these limitations, a detailed description of an adult male frog's anatomy (as a complete organism) using a non-destructive micro-CT method was recently provided (Porro and Richards 2017). Micro-CT, to be candid, has already been employed to some extent as a valuable tool in selected stages of X. laevis' embryos, tadpoles, and frogs to explore localized or specific occurrences, such as gastrulation (Moosmann, Ershov et al. 2014), craniofacial (Slater, Liu et al. 2009) and urostyle (Senevirathne, Baumgart et al. 2020) development, skeletal morphology (Vasquez, Hansen et al. 2008, Matthews and du Plessis 2016), limb (skeletal) regeneration (Feng, Milner et al. 2011, Chen, Lin et al. 2012, Golding, Guay et al. 2016), along with brain regeneration (Ishii, Yoshida et al. 2023), and the anatomy of cranial and anterior spinal nerves (Naumann and Olsson 2018), the head (Descamps, Sochacka et al. 2020, Murugan, Vigran et al. 2022), and several internal organs (Descamps, Sochacka et al. 2014). Besides X. laevis itself, micro-CT has been utilized in closely related frogs to investigate, for instance, chondrocranium organization in A. obstetricans (Krings, Muller et al. 2017), skeleton (Herrel and Bonneaud 2012) and digit (Zhang, Wilson et al. 2021) morphology in X. tropicalis, cortical bone morphology in various anuran amphibians (Kondo, Iwamoto et al. 2023), re-evolution of lost mandibular teeth in G. guentheri (Paluh, Dillard et al. 2021, Paluh, Riddell et al. 2021), together with cranial musculoskeletal structures in P. fuscus (Jandausch, Schwarz et al. 2022), lungs rearrangements during metamorphosis in M. fissipes (Chang, Zhang et al. 2022), gill formation during metamorphosis in B. bufo (Kraus and Metscher 2022), and metabolic reorganization in metamorphic R. omeimontis tadpoles (Zhu, Chang et al. 2020). Nevertheless, the spatiotemporal dynamics of X. laevis' late development on the level of a whole organism, along with the comparison between adult male and female frogs, remain largely unexplored in sufficient detail.

3. An opportunity may have been missed here to provide some truly new biological insights: The gut remodels substantially during metamorphosis, but to my knowledge that has NOT been previously examined by microCT. It may not work, as the gut may simply be too soft to visualize, but then again, it may be worth trying.

We appreciate the reviewer's suggestion and have exerted considerable effort to provide further insights into the development of the Xenopus gut using our samples (refer to p. 14 and Figure 7).

EDITOR'S COMMENT:
In addition, please register any new software application in the bio.tools and SciCrunch.org databases to receive RRID (Research Resource Identification Initiative ID) and biotoolsID identifiers, and include these in your manuscript. Computational workflows should be registered in workflowhub.eu and the DOIs cited in the relevant places in the manuscript. These will facilitate tracking, reproducibility and re-use of your tool.

As for RRIDs, we implemented this sentence into the manuscript (p. 7):
The reconstructed data were imported into VG Studio MAX 2023.4 software (Volume Graphics GmbH, Heidelberg, Germany), in which the measured data were segmented, analyzed, and visualized using VG Studio MAX, RRID: SCR_017997. The segmentation of Xenopus guts was done manually by an operator using the software Avizo 2020.2 (Thermo Fisher Scientific, Waltham, MA, USA) (Avizo 3D Software, RRID: SCR_014431).
