# Supplementary material for: Unveiling vertebrate development dynamics in frog Xenopus laevis using micro-CT imaging
Source: Gigascience. 2024 Jul 17;13:giae037. doi: 10.1093/gigascience/giae037 (PMC12558079; doi:10.1093/gigascience/giae037)
Supplement: giae037_Reviewer_1_Report_Original_Submission — Brian Metscher -- 12/29/2023 Reviewed [file giae037_Reviewer_1_Report_Original_Submission.pdf]

Reviewer Report

Title: Unveiling Vertebrate Development Dynamics in Frog Xenopus laevis using Micro-CT Imaging

Version: Original Submission **Date:** 12/29/2023

Reviewer name: Brian Metscher

Reviewer Comments to Author:

Comments on GIGA-D-23-00370
Laznovsky et al.
The authors present a set of 3D images of selected developmental stages of the widely-used laboratory model Xenopus laevis along with some examples of how the data might be used in developmental analyses. The dataset covers stages from mid-larva through metamorphosis to adult, which should provide a starting point for various studies of morphological development. Some studies will undoubtedly require other stages or more detailed images, but the presented data were collected with straightforward methods that will allow compatibility with future work.
The data appear to be sound in the collection and curation. Data availability is made clear in the article, and the complete set will be publicly available in standard formats on the Zenodo repository. This should ensure full accessibility to anyone interested.
The article is well-organized and clearly written.
A few points about the methods could be clarified:
Was only one specimen per stage scanned?
Specimens were dehydrated through an ethanol series and then stained with free iodine in 90% methanol, and then rehydrated back through ethanol. Why was methanol used for the staining and not dehydration? It seems odd to switch alcohols back and forth without intermediate steps. This could have some effect on tissue shrinkage.
It should be indicated that the X-ray source target is tungsten (even though it is unlikely to be anything else in this machine).
The "real images" (p. 7) in Suppl. Fig. 1 should simply be called photographs - microCT images are real too.
For the measurements of bone mass, is the cartilage itself actually visible in the microCT images?
p. 13: "The dataset's diverse species representation…" What does this mean? It is only one species.
The limitations on the image data are not discussed. All images have limits to their useful resolution and contrast among components; this is not a weakness, just a reality of imaging.
The different reconstructed voxel sizes for different size specimens are mentioned, but it might be helpful to indicate the voxel sizes in Figure 1 as well as in the relevant table. And if the middle column of Figure 1 could be published with full resolution of the snapshots it would help show the actual quality of the images.
Brian Metscher, University of Vienna

Methods

Are the methods appropriate to the aims of the study, are they well described, and are necessary controls included? Choose an item.

Conclusions

Are the conclusions adequately supported by the data shown? Choose an item.

Reporting Standards

Does the manuscript adhere to the journal's guidelines on minimum standards of reporting? Choose an item.

Choose an item.

Statistics

Are you able to assess all statistics in the manuscript, including the appropriateness of statistical tests used? Choose an item.

Quality of Written English

Please indicate the quality of language in the manuscript: Choose an item.

Declaration of Competing Interests

Please complete a declaration of competing interests, considering the following questions:

- Have you in the past five years received reimbursements, fees, funding, or salary from an organisation that may in any way gain or lose financially from the publication of this manuscript, either now or in the future?
- Do you hold any stocks or shares in an organisation that may in any way gain or lose financially from the publication of this manuscript, either now or in the future?
- Do you hold or are you currently applying for any patents relating to the content of the manuscript?
- Have you received reimbursements, fees, funding, or salary from an organization that holds or has applied for patents relating to the content of the manuscript?
- Do you have any other financial competing interests?
- Do you have any non-financial competing interests in relation to this paper?

If you can answer no to all of the above, write 'I declare that I have no competing interests' below. If your reply is yes to any, please give details below.

I declare that I have no competing interests.

I agree to the open peer review policy of the journal. I understand that my name will be included on my report to the authors and, if the manuscript is accepted for publication, my named report including any attachments I upload will be posted on the website along with the authors' responses. I agree for my report to be made available under an Open Access Creative Commons CC-BY license (http://creativecommons.org/licenses/by/4.0/). I understand that any comments which I do not wish to be included in my named report can be included as confidential comments to the editors, which will not be published.

Choose an item.

To further support our reviewers, we have joined with Publons, where you can gain additional credit to further highlight your hard work (see: https://publons.com/journal/530/gigascience). On publication of this paper, your review will be automatically added to Publons, you can then choose whether or not to claim your Publons credit. I understand this statement.

Yes Choose an item.
