# Supplementary material for: Unveiling vertebrate development dynamics in frog Xenopus laevis using micro-CT imaging
Source: Gigascience. 2024 Jul 17;13:giae037. doi: 10.1093/gigascience/giae037 (PMC12558079; doi:10.1093/gigascience/giae037)
Supplement: giae037_Reviewer_2_Report_Original_Submission — Virgilio Gail Ponferrada, Ph.D. -- 1/5/2024 Reviewed [file giae037_Reviewer_2_Report_Original_Submission.pdf]

Reviewer Report

Title: Unveiling Vertebrate Development Dynamics in Frog Xenopus laevis using Micro-CT Imaging

Version: Original Submission **Date:** 1/5/2024

Reviewer name: Virgilio Gail Ponferrada, Ph.D.

Reviewer Comments to Author:

Reviewer's comments for Laznovsky et al. manuscript GIGA-D-23-00370:
The manuscript is well written and easy to understand. It will be a good contribution to the Xenopus research community as well as a useful reference for the field of developmental and amphibian biology.
I suggest the following revisions:
- For the graphical abstract try alternating NF stage numbers above and below samples for a cleaner look, adult male and adult female can both remain at the top.
- Appreciate the rationale for providing the microCT analysis presented in this manuscript and choices of late stage tadpoles, pre- and prometamorphosis, through metamporphosis to the adult male and female frog.
- For the head development section authors can make reference to the Xenhead drawings, Zahn et al. Development 2017.
- Head Development section paragraph 4, change word from "gender" to "sex."
- Supplementary Table 3. Change "gender-related" to "sex-related."
- Micro-CT Data Analysis of Long Bone Growth Dynamics section paragraph 1 change "in terms of gender" to "in terms of sex."
- Figure 4 panels A and B don't reflect the observation that adult females are enlarged males. While the authors state that the view of the male and female skeletons are maximized and not proportional as stated in the caption, suggest that scale bars be employed and the images adjusted to show the size relationship difference between the sexes as in Figure 1. On first glance and perhaps to those not as familiar with the difference in sex size in Xenopus that in this particular example of the adult male image being more spread out compared to the image of the female, it feels misleading.
- Ossification Analysis section paragraph 2 change "frog's gender" to "frog's sex."
- Figure 5 panel A, the label is overlapping "NF 59." For panels B and B' scale bars on these panels would help the reader understand the proportions. Yes, there is the 3mm scale bar from panel A and as stated in the caption, but including them in the B panels could help even if panel B had a scale bar labeled at 0.25 mm and panel B' was 3 mm.
- Segmentation of Selected Internal Soft Organ section, perhaps more commentary on the ability to observe the development of the segmentation of the brain regions: cbh:
cerebral hemispheres; cbl: cerebellum; dch: diencephalon; mob: medulla oblongata; opl: optic lobes; sp: spinal cord while clearly shown in Figure 6, some accompanying description in the text would help readers in general or give the implication that microCT analysis of mutant or diseased frogs could help identify physical characteristics of frogs with developmental or neurological disorders. This would help transition from the analysis of a specific organ to the next section Further Biological Potential of

Xenopus's Data.
- These analyses, while thorough accompanied by novel visuals, require statistical implementation of multiple tadpoles and frogs per NF stage to account for variation in samples and to bolster the claims stated in skull thickness, the head mass and eye distance changes, increased length of the long bones during maturation, and femural ossification cartilage to bone ratios. This may constitute a suggested major revision to perform these analyses.

Methods

Are the methods appropriate to the aims of the study, are they well described, and are necessary controls included? Choose an item.

Conclusions

Are the conclusions adequately supported by the data shown? Choose an item.

Reporting Standards

Does the manuscript adhere to the journal's guidelines on minimum standards of reporting? Choose an item.

Choose an item.

Statistics

Are you able to assess all statistics in the manuscript, including the appropriateness of statistical tests used? Choose an item.

Quality of Written English

Please indicate the quality of language in the manuscript: Choose an item.

Declaration of Competing Interests

Please complete a declaration of competing interests, considering the following questions:

- Have you in the past five years received reimbursements, fees, funding, or salary from an organisation that may in any way gain or lose financially from the publication of this manuscript, either now or in the future?
- Do you hold any stocks or shares in an organisation that may in any way gain or lose financially from the publication of this manuscript, either now or in the future?
- Do you hold or are you currently applying for any patents relating to the content of the manuscript?
- Have you received reimbursements, fees, funding, or salary from an organization that holds or has applied for patents relating to the content of the manuscript?

- Do you have any other financial competing interests?
- Do you have any non-financial competing interests in relation to this paper?

If you can answer no to all of the above, write 'I declare that I have no competing interests' below. If your reply is yes to any, please give details below.

I declare that I have no competing interests

I agree to the open peer review policy of the journal. I understand that my name will be included on my report to the authors and, if the manuscript is accepted for publication, my named report including any attachments I upload will be posted on the website along with the authors' responses. I agree for my report to be made available under an Open Access Creative Commons CC-BY license (http://creativecommons.org/licenses/by/4.0/). I understand that any comments which I do not wish to be included in my named report can be included as confidential comments to the editors, which will not be published.

Choose an item.

To further support our reviewers, we have joined with Publons, where you can gain additional credit to further highlight your hard work (see: https://publons.com/journal/530/gigascience). On publication of this paper, your review will be automatically added to Publons, you can then choose whether or not to claim your Publons credit. I understand this statement.

Yes Choose an item.
