# Supplementary material for: Unveiling vertebrate development dynamics in frog Xenopus laevis using micro-CT imaging
Source: Gigascience. 2024 Jul 17;13:giae037. doi: 10.1093/gigascience/giae037 (PMC12558079; doi:10.1093/gigascience/giae037)
Supplement: giae037_Reviewer_3_Report_Original_Submission — John Wallingford -- 1/10/2024 Reviewed [file giae037_Reviewer_3_Report_Original_Submission.pdf]

Reviewer Report

Title: Unveiling Vertebrate Development Dynamics in Frog Xenopus laevis using Micro-CT Imaging

Version: Original Submission **Date:** 1/10/2024

Reviewer name: John Wallingford

Reviewer Comments to Author:

Laznovsky et al. present a nice compendium of micro-CT-based digital volumes of several stages of Xenopus development. Given the prominence of this important model animal in studies of developmental biology and physiology, this dataset is quite useful and will be of interest to the community. That said, the study has some key limitations that will limit its utility for the research community, though these do not reduce the dataset's impact in the education and popular science realms, which is also a stated goal for the paper. Overall, I recommend publication after an effort has been made to address the following concerns.

1. The atlas adequately samples developmental stages from late tadpole through metamorphosis. However, as far as I can tell only a single sample has been imaged at each stage. Thus, the quantifications of inter-stage differences shown here (Fig. 2, 4, 5) are at best very rough estimates and also provide no information about intra-stage variability in these metrics. This is not a fatal weakness, but it is an important caveat that I believe should be very explicitly stated in the text and in the figure legend of relevant figures.
2. I am very disappointed that the rich history of microCT on Xenopus seems to have been entirely ignored by these authors. MicroCT has already been used to describe the skull, the brain, liver, blood vessels, etc. during Xenopus development. (Just a few papers the authors should read are: Slater et al., PLoS One 2009; Senevirathnea et al., PNAS, 2019; Ishii et al., Dev. Growth, Diff. 2023; Zhu et al., Front. Zool 2020.) It has also been used for comparative studies of other frogs (Kondo et al., Dev. Growth, Diff. 2022; Kraus, Anat. Rec. 2021; Jandausch et al., Zool. Anz. 2022; Paluh, et al., Evolution 2021, Paluh et al., eLife 2021). None of these -or the many other relevant papers- are discussed or cited here. The research community would be much better served if authors make a serious effort to integrate their methods and their results into this existing literature.
3. An opportunity may have been missed here to provide some truly new biological insights: The gut remodels substantially during metamorphosis, but to my knowledge that has NOT be previously examined by microCT. It may not work, as the gut may simply be too soft to visualize, but then again, it may be worth trying.

Methods

Are the methods appropriate to the aims of the study, are they well described, and are necessary controls included? Choose an item.

Conclusions

Are the conclusions adequately supported by the data shown? Choose an item.

Reporting Standards

Does the manuscript adhere to the journal's guidelines on minimum standards of reporting? Choose an item.

Choose an item.

Statistics

Are you able to assess all statistics in the manuscript, including the appropriateness of statistical tests used? Choose an item.

Quality of Written English

Please indicate the quality of language in the manuscript: Choose an item.

Declaration of Competing Interests

Please complete a declaration of competing interests, considering the following questions:

- Have you in the past five years received reimbursements, fees, funding, or salary from an organisation that may in any way gain or lose financially from the publication of this manuscript, either now or in the future?
- Do you hold any stocks or shares in an organisation that may in any way gain or lose financially from the publication of this manuscript, either now or in the future?
- Do you hold or are you currently applying for any patents relating to the content of the manuscript?
- Have you received reimbursements, fees, funding, or salary from an organization that holds or has applied for patents relating to the content of the manuscript?
- Do you have any other financial competing interests?
- Do you have any non-financial competing interests in relation to this paper?

If you can answer no to all of the above, write 'I declare that I have no competing interests' below. If your reply is yes to any, please give details below.

None.

I agree to the open peer review policy of the journal. I understand that my name will be included on my report to the authors and, if the manuscript is accepted for publication, my named report including any attachments I upload will be posted on the website along with the authors' responses. I agree for my report to be made available under an Open Access Creative Commons CC-BY license (http://creativecommons.org/licenses/by/4.0/). I understand that any comments which I do not wish to

be included in my named report can be included as confidential comments to the editors, which will not be published.

Choose an item.

To further support our reviewers, we have joined with Publons, where you can gain additional credit to further highlight your hard work (see: https://publons.com/journal/530/gigascience). On publication of this paper, your review will be automatically added to Publons, you can then choose whether or not to claim your Publons credit. I understand this statement.

Yes Choose an item.
